# Synthesis of skeletally diverse alkaloid-like molecules: exploitation of metathesis substrates assembled from triplets of building blocks

**DOI:** 10.3762/bjoc.9.88

**Published:** 2013-04-22

**Authors:** Sushil K Maurya, Mark Dow, Stuart Warriner, Adam Nelson

**Affiliations:** 1 School of Chemistry, University of Leeds, Leeds, LS2 9JT, UK; 2 Astbury Centre for Structural Molecular Biology, University of Leeds, Leeds, LS2 9JT, UK

**Keywords:** alkaloids, cyclopropanes, diversity-oriented synthesis, metathesis

## Abstract

A range of metathesis substrates was assembled from triplets of unsaturated building blocks. The approach involved the iterative attachment of a propagating and a terminating building block to a fluorous-tagged initiating building block. Metathesis cascade chemistry was used to “reprogram” the molecular scaffolds. Remarkably, in one case, a cyclopropanation reaction competed with the expected metathesis cascade process. Finally, it was demonstrated that the metathesis products could be derivatised to yield the final products. At each stage, purification was facilitated by the presence of a fluorous-tagged protecting group.

## Introduction

Our collective understanding of the biological relevance of chemical space has been shaped, in large part, by the historic exploration of chemical space by chemical synthesis (and biosynthesis) [1]. The scaffolds of known bioactive small molecules, in particular, play a key role in guiding the navigation of chemical space [2–4]. The field of biology-oriented synthesis (BIOS) [5], for example, uses biologically validated scaffolds [6–8] to inspire library design.

Known organic molecules populate chemical space unevenly and unsystematically. Around half of all known organic compounds are based on only 0.25% of the known molecular scaffolds [9]! This uneven coverage of chemical space is also typical of small-molecule screening collections [7,10]. Consequently, the biological relevance of most known scaffolds has been poorly explored. The field of diversity-oriented synthesis [11–13] has emerged with the specific aim of populating screening collections with diverse and novel small molecules.

**Scheme 1:**
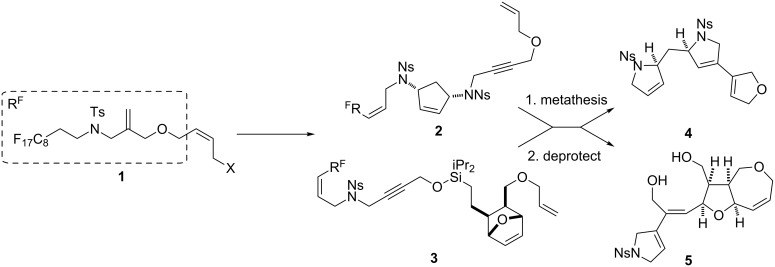
Illustrative examples of a synthetic approach to natural-product-like molecules with over eighty molecular scaffolds.

We have previously developed a robust approach for the synthesis of skeletally diverse small molecules (Scheme 1) [14]. The approach relied on the synthesis of metathesis substrates by iterative attachment of simple unsaturated building blocks to a fluorous-tagged linker **1** (e.g., → **2** or **3**). Subsequently, metathesis cascade reactions were used to “reprogram” the molecular scaffolds, concomitantly releasing the products from the linker (e.g., → **4** or **5**) [14–17]. The approach enabled the combinatorial variation of molecular scaffolds, and was exploited in the synthesis of natural-product-like small molecules with unprecedented scaffold diversity (over 80 distinct scaffolds).

Although powerful, this general approach to skeletally diverse molecules had only been exemplified by varying pairs of unsaturated building blocks [14]. Thus, by exploiting the linker **1**, which is an allyl alcohol or allyl amine equivalent, all of the products were inevitably allylic alcohols or cyclic allylic amines. Here, we demonstrate that the approach is considerably more general, and that it is feasible to exploit triplets of building blocks, extending the range of diverse molecular scaffolds that may be prepared.

## Results and Discussion

### Library design

**Scheme 2:**
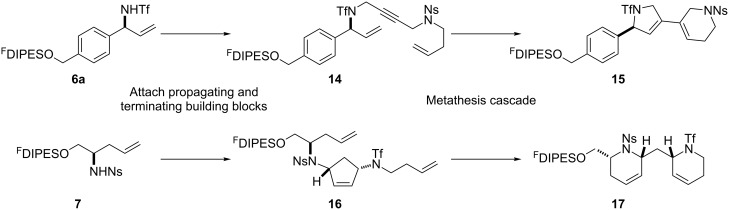
Overview of the proposed synthetic approach. ^F^DIPES = diisopropyl(3,3,4,4,5,5,6,6,7,7,8,8,9,9,10,10,10-heptadecafluorodecyl)silyl; Ns = *o*-nitrophenylsulfonyl.

**Figure 1:**
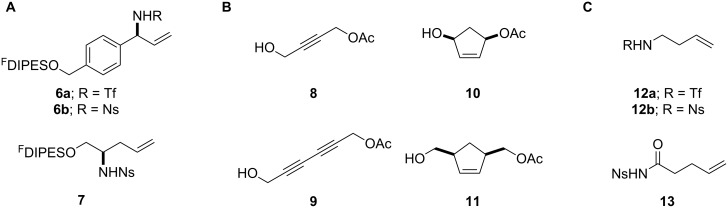
Structures of building blocks used in this study. Panel A: fluorous-tagged initiating building blocks. Panel B: propagating building blocks. Panel C: terminating building blocks.

An overview of the proposed approach to the synthesis of diverse scaffolds is shown in Scheme 2. The building blocks used in this study are shown in Figure 1. It was planned to start with an “initiating” building block (e.g., **6a** or **7**) bearing a fluorous tag to facilitate the purification of synthetic intermediates [18]. Iterative attachment of a propagating and a terminating building block would yield a metathesis substrate (such as **14** or **16**). Finally, a metathesis cascade reaction would yield a product scaffold (such as **15** and **17**). It was planned that many of the product scaffolds would bear an *o-*nitrophenylsulfonyl protecting group. The combinations of building blocks were carefully chosen to ensure that, after deprotection, selective derivatisation of the product scaffolds would be possible.

### Synthesis of building blocks

**Scheme 3:**
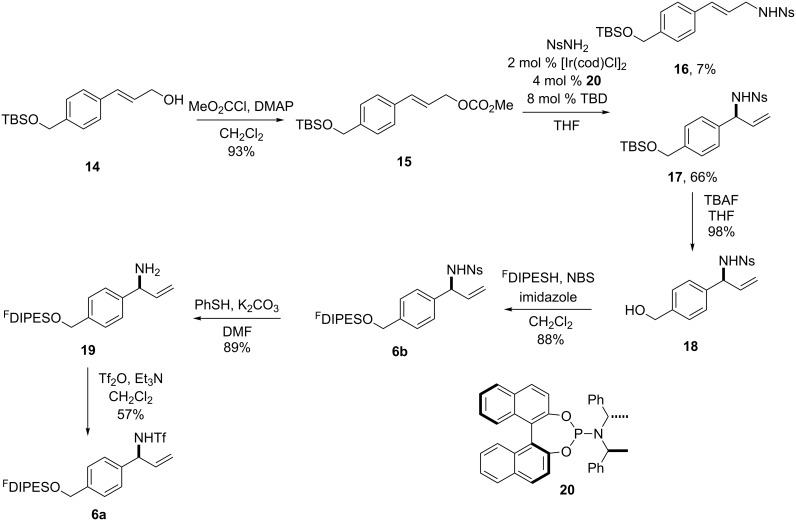
Synthesis of the initiating building blocks **6a** and **6b**. TBD = 1,5,7-triazabicyclo[4.4.0]dec-5-ene.

The initiating building blocks **6a** and **6b** were prepared by using the approach outlined in Scheme 3. The allylic alcohol **14** [19] was converted into the allylic carbonate **15** by treatment with methyl chloroformate and DMAP. The allylic carbonate **15** underwent efficient asymmetric allylic amination [20] with *o*-nitrophenylsulfonamide as the nucleophile to give the allylic sulfonamide **17** in 66% yield; in addition, the linear product **16** was also obtained in 7% yield. Desilylation of **17** (→ **18**) and reaction with diisopropyl(3,3,4,4,5,5,6,6,7,7,8,8,9,9,10,10,10-heptadecafluorodecyl)silyl (^F^DIPES) bromide, generated in situ from the corresponding silyl hydride, gave the fluorous-tagged building block **6b**. Finally, desulfonylation (→ **19**) and trifluoromethylsulfonylation yielded the alternative initiating building block **6a**.

**Scheme 4:**
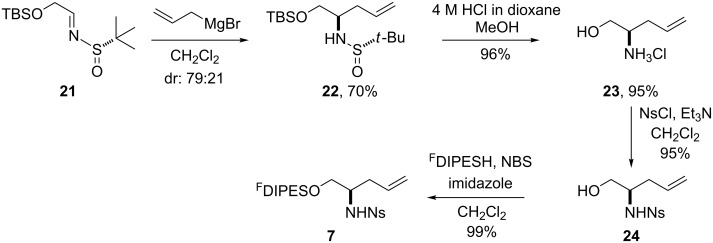
Synthesis of the initiating building block **7**.

The initiating building block **7** was prepared from the sulfinimine **21** by adapting a synthesis previously reported by Ellman (Scheme 4) [21]. Treatment of the sulfinimine **21** in dichloromethane with allylmagnesium bromide yielded the corresponding sulfinimides as a 79:21 mixture of diastereoiomers; following column chromatography, the major diastereomer **22** was obtained in 70% yield, and was converted into the corresponding amino alcohol **23**. The configuration of the amino alcohol **23** was determined by conversion into the corresponding benzamide and comparison with racemic and enantiomerically enriched samples (prepared from the commercially available amino acid). Analysis by chiral HPLC indicated that the amino alcohol **23** had (*R*)-configuration. It was concluded that the sense of diastereoselectivity in the addition **21** → **22** contrasted with that reported by Ellman [21]. However, the sense of diastereoselectivity was the same as that reported for the addition of allylmagnesium bromide in dichloromethane to a similar sulfinimine [22]. The amino alcohol **23** was converted into the corresponding *o*-nitrophenylsulfonamide **24** and, hence, the fluorous-tagged building block **7**.

The propagating building blocks **8**–**11**, and the terminating building block **12b**, were prepared by using established methods [14]. The enantiomeric excess (68% ee) of the hydroxy alcohol **11** was determined by conversion into the corresponding diastereomeric *O-*methyl mandelate esters. The terminating building blocks **12a** and **13** were prepared by straightforward derivatisation of commercially available starting materials (see Supporting Information File 1).

### Synthesis of metathesis substrates

Initially, the propagating building blocks **8**–**11** were attached to the fluorous-tagged initiating building blocks (**6a**, **6b** or **7**). In each case, an excess of the propagating building block, DEAD and triphenylphosphine was used. In general, the crude product was directly deacetylated. At each stage, the required fluorous-tagged product was isolated by fluorous-solid-phase extraction (F-SPE), and its purity determined by analysis by 500 MHz ^1^H NMR spectroscopy. These results are summarised in Table 1.

**Table 1:** Attachment of propagating building blocks to the fluorous-tagged initiating building blocks.

Building blocks	Attachment	Deacetylation^a^	Product

	Method^a^ (mass recovery / %) {Purity^b^ / %}	Mass recovery / % (Purity^b^ / %)	

**6a**, **8**	A1 (70) {>98}	92 (92)	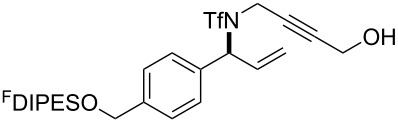 **25**
**6a**, **9**	A2 (97) {92}	87 (93)	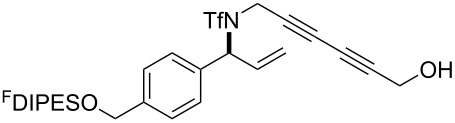 **26**
**6a**, **10**^c^	A3 (85) {>98}	87 (98)	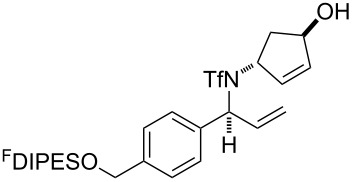 **27**
**6b**, **11^d^**	A3 (85) {76}	85^e^ (72)	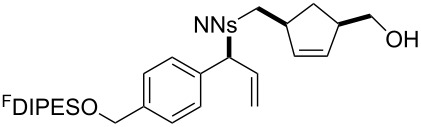 **28**
**7**, **8**	A3 (92) {91}	94^f^	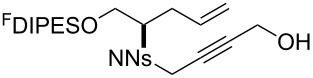 **29**
**7**, **9**	A3 (74^f^)	97 (98)	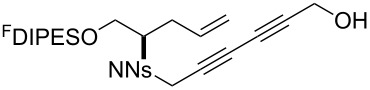 **30**
**7**, **10**	A3 (97) {91}	80^f^	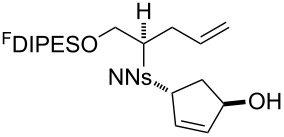 **31**

^a^Methods: A1: Initiating building block (1.0 equiv), propagating building block (4.0 equiv), DEAD (4.0 equiv), PPh_3_ (4.0 equiv), CH_2_Cl_2_, 0 °C → rt then F–SPE; A2: Initiating building block (1.0 equiv), propagating building block (4.0 equiv), DEAD (2.0 equiv), PPh_3_ (2.0 equiv), CH_2_Cl_2_, 0 °C → rt then F–SPE; A3: Initiating building block (1.0 equiv), propagating building block (4.0 equiv), DEAD (2.0 equiv), PPh_3_ (2.0 equiv), THF, 0 °C → rt then F–SPE; Deacetylation: 0.025 M NH_3_ in MeOH. ^b^Determined by analysis of the 500 MHz ^1^H NMR spectrum. ^c^The building block had >98% ee. ^d^The building block had 68% ee. ^e^Isolated as a ca. 75:25 mixture of diastereoisomers. ^f^Isolated yield of purified product (see Supporting Information File 1).

The metathesis substrates were prepared by subsequent attachment of a terminating building block (**12a**, **12b** or **13**) (see Table 2). In each case, an excess of the terminating building block, DEAD and triphenylphosphine was used; the required fluorous-tagged product was isolated by solid-fluorous phase extraction (F-SPE), and its purity was determined by analysis by 500 MHz ^1^H NMR spectroscopy.

**Table 2:** Attachment of propagating building blocks to the fluorous-tagged initiating building blocks.

Substrate	Terminating building block	Attachment	Product

		Method^a^ (mass recovery / %) {Purity^b^ / %}	

**25**	**12b**	A4 (89) {83}	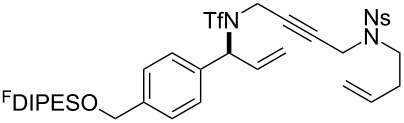 **32**
**25**	**13**	A4 (89) {86}	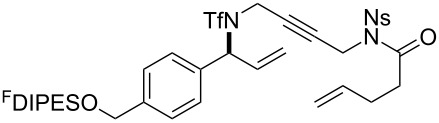 **33**
**26**	**12b**	A4 (76) {93}]	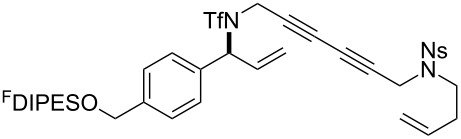 **34**
**26**	**13**	A4 (75) {97}	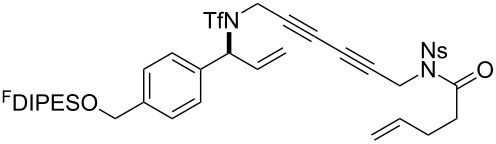 **35**
**27**	**12b**	A5 (62^c^)	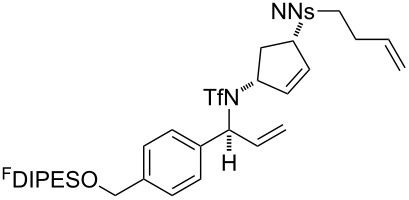 **36**
**27**	**13**	A5 (54^c^)	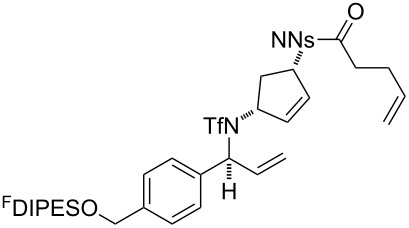 **37**
**28**^d^	**12a**	A5 (86^c,e^)	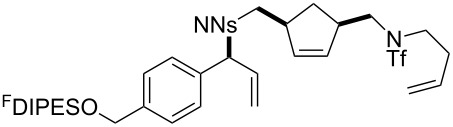 **38**
**28**^d^	**13**	A5 (77^c,e^)	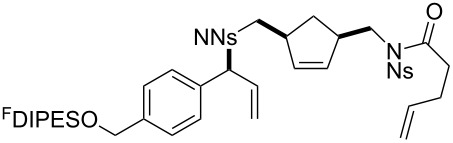 **39**
**29**	**12a**	A6 (86^c^)	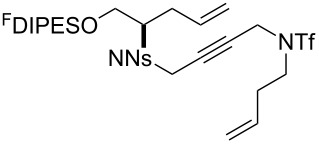 **40**
**29**	**13**	A6 (77^c^)	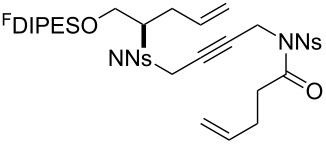 **41**
**30**	**12a**	A6 (92^c^)	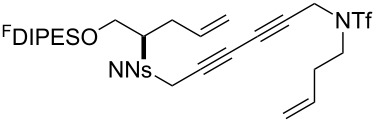 **42**
**30**	**13**	A6 (55^c^)	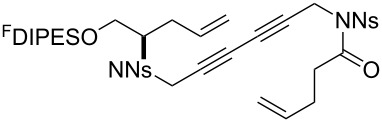 **43**
**31**	**12a**	A6 (85^c^)	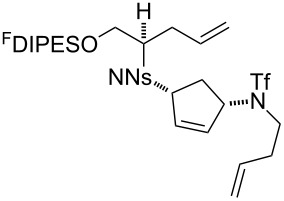 **44**

^a^Methods: A4: Substrate (1.0 equiv), propagating building block (4.0 equiv), DEAD (2.0 equiv), PPh_3_ (2.0 equiv), CH_2_Cl_2_, 0 °C → rt then F-SPE; A5: Substrate (1.0 equiv), propagating building block (4.0 equiv), DEAD (4.0 equiv), PPh_3_ (4.0 equiv), THF, 0 °C → rt then F-SPE; A6: Substrate (1.0 equiv), propagating building block (4.0 equiv), DEAD (2.0 equiv), PPh_3_ (2.0 equiv), THF, 0 °C → rt then F-SPE. ^b^Determined by analysis of the 500 MHz ^1^H NMR spectrum. ^c^Isolated yield of purified product. ^d^The starting material was a ca. 75:25 mixture of diastereoisomers. ^e^Isolated as a ca. 75:25 mixture of diastereomers.

### Metathesis cascade reactions

The scaffolds of the metathesis substrates were “reprogrammed” by treatment with Hoveyda–Grubbs second-generation catalyst in either dichloromethane or *tert*-butyl methyl ether [23] (TBME). Many of the metathesis reactions were rather sluggish, and the catalyst was added portionwise until the reactions were judged to be complete by TLC analysis. After removal [24] of the catalyst by using tris(hydroxymethyl)phosphine, the metathesis products were generally purified by flash column chromatography. Finally, the *o*-nitrophenylsulfonyl groups were removed from the products. The results are summarised in Table 3.

**Table 3:** Application of cascade metathesis reactions in the synthesis of diverse scaffolds and subsequent desulfonylation.

Substrate	Method^a^ (mol %; time)	Product	Yield / %

**32**	B1 (5 + 2.5; 3 d) then C1	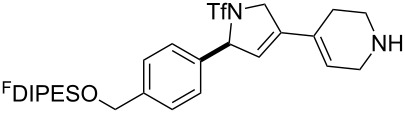 **45**	**45**, 37%^b^
		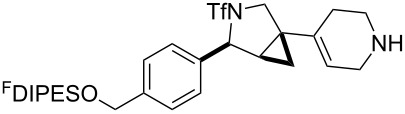 **46**	**46**, 11%^b^
		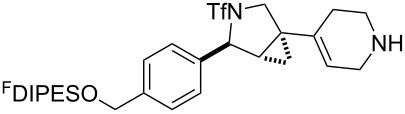 **47**	**47**, 5%^b^
**33**	B1 (2 × 5; 4 d) then C1 then D	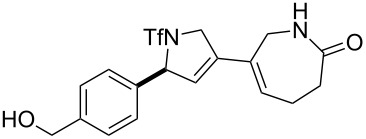 **48**	43%^b^
**34**	B1 (5 + 5 + 2.5; 10 d) then C1	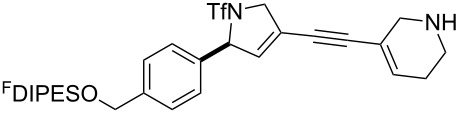 **49**	49%^b^ (86%^c^)
**36**	B1 (4 × 5; 20 d) then C1	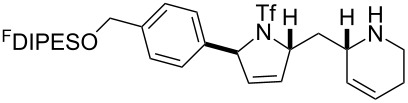 **50**	77 then 81 (93%^c^)
**38**^d^	B1 (3 × 5; 14 d) then C1	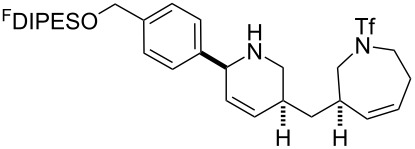 **51**	63 then 85 (**51**)
		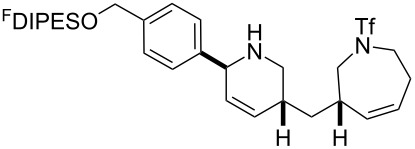 **52**	29 then 94 (**52**)
**39**	B1 (4 × 5; 20 d) then C2	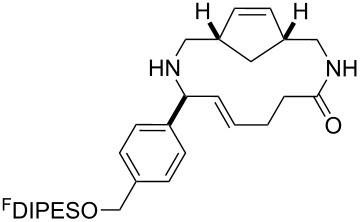 **53**	8%^b^
**40**	B2 (5; 24 h) then C1	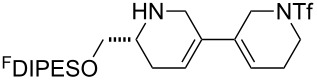 **54**	93 then 96 (87%^c^)
**41**	B2 (5; 24 h) then C2	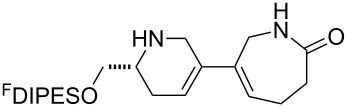 **55**	93 then 80 (93%^c^)
**42**	B2 (3 × 5; 7 d) then C1	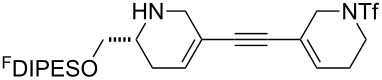 **56**	76 then 92 (92%^c^)
**43**	B2 (2 × 5; 3 d) then C2	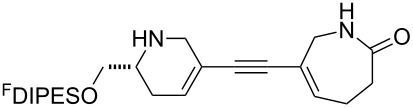 **57**	54 then 77 (86%^c^)
**44**	B2 (5; 24 h) then C1	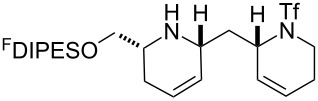 **58**	53 then 99 (98%^c^)

^a^Methods: B1: Hoveyda–Grubbs second-generation catalyst, CH_2_Cl_2_, 50 °C then Et_3_N (86 equiv), P(CH_2_OH)_3_ (86 equiv) then silica; B2: Hoveyda–Grubbs second-generation catalyst, MTBE, 50 °C then Et_3_N (86 equiv), P(CH_2_OH)_3_ (86 equiv) then silica; C1: PhSH (1.2 equiv), K_2_CO_3_ (3.0 equiv), DMF; C2: PhSH (2.4 equiv), K_2_CO_3_ (6.0 equiv), DMF; E: aq HF, MeCN–CH_2_Cl_2_. ^b^Yield over more than one step. ^c^Purity of the product determined by 500 MHz ^1^H NMR spectroscopy. ^d^The starting material was a ca. 75:25 mixture of diastereoisomers.

**Scheme 5:**
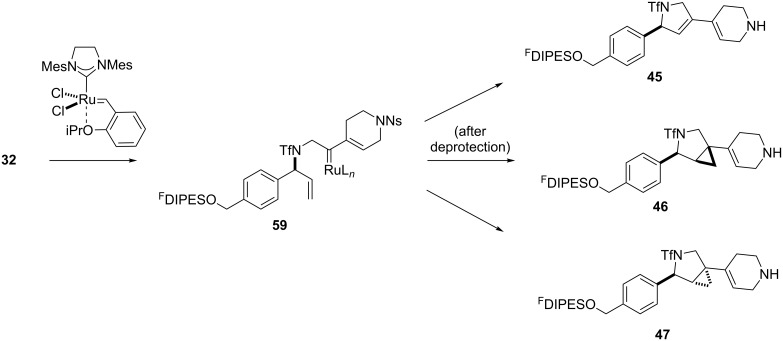
Fate of the metathesis reaction of the substrate **32**.

In general, the metathesis reactions proceeded smoothly to give the expected metathesis cascade products. In the case of **39**, however, the cyclopentene did not participate in the metathesis reaction, and the bridged macrocycle **53** was obtained in low yield. We have previously observed the formation of macrocyclic metathesis products in similar metathesis cascade reactions [14]. The formation of the cyclopropanes **46** and **47** as byproducts in the metathesis cascade reaction of **32** was remarkable [25]. Presumably, in this case, the metathesis cascade leads to the generation of the intermediate **59** (Scheme 5); the intermediate could then react to conclude the metathesis cascade (to give **45** after deprotection), or cyclopropanate [25] the terminal alkene (to give **46** or **47** after deprotection) (Scheme 5).

Finally, a selection of fluorous-tagged products was derivatised (typically on a 50 μmol scale) to yield a range of amides and ureas (Table 4). The fluorous tag facilitated the purification of the derivitised products by F-SPE. The final products **60**–**71** (Table 4) were obtained after removal of the fluorous tag by desilylation.

**Table 4:** Derivatisation and deprotection of final products.

Substrate (purity^a^ / %)	Product^b^		Method^c^	Yield / %

**45**	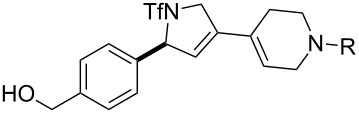	**60a**	D	51
		**60b**	E1 then D	81 then 60
		**60c**	E2 then D	67 then 98
		**60d**	E3 then D	94 then 81

**46**	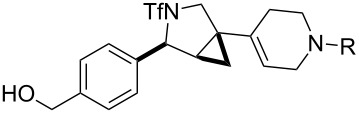	**61b**	E1 then D	39 then 70

**47**	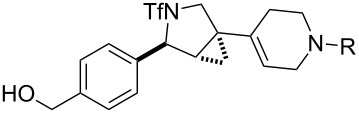	**62b**	E1 then D	43 then 63

**49** (86)	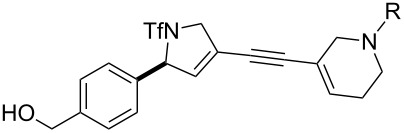	**63a**	D	83
		**63b**	E1 then D	32 then 64
		**63c**	E2 then D	83 then 58
		**63d**	E3 then D	84 then 79

**50** (93)	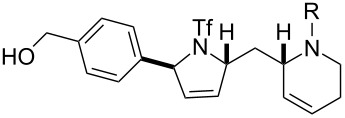	**64a**	D	87
		**64b**	E1 then D	29^d^
		**64c**	E2 then D	43^d^
		**64d**	E3 then D	34^d^

**51** (85)	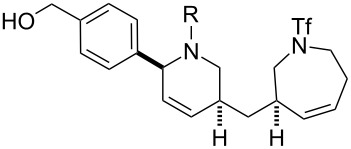	**65a**	D	70
		**65b**	E1 then D	40^d^
		**65c**	E2 then D	82^d^
		**65d**	E3 then D	74^d^

**53**	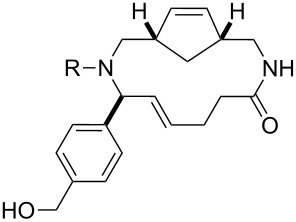	**66a**	D	52

**54** (87)	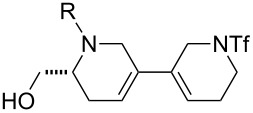	**67a**	D	91
		**67b**	E4 then D	77^d^
		**67c**	E2 then D	83^d^
		**67d**	E3 then D	42^d^

**55** (93)	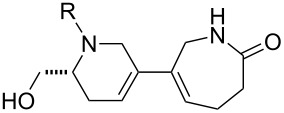	**68a**	D	94
		**68c**	E2 then D	67^d^
		**68d**	E3 then D	40^d^

**56** (92)	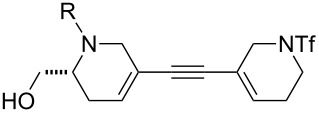	**69a**	D	91
		**69b**	E4 then D	67^d^
		**69c**	E2 then D	67^d^

**57** (86)	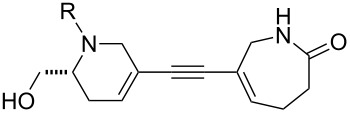	**70a**	D	47
		**70c**	E2 then D	59^d^

**58** (98)	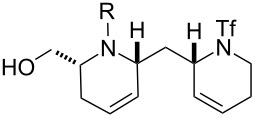	**71a**	D	91
		**71b**	E4 then D	64^d^
		**71c**	E2 then D	53^d^
		**71d**	E3 then D	29^d^

^a^Determined by analysis of the product by 500 MHz ^1^H NMR spectroscopy. ^b^The suffix refers to the identity of the R substituent: a, R = H; b, R = isoxazole-5-carbonyl; c, R = pyridine-3-carbonylamino; d, R = morpholine-4-carbonyl; ^c^Methods: D: aq HF, MeCN–CH_2_Cl_2_; E1: isoxazole-5-carbonyl chloride (2.0 equiv), Et_3_N (3.0 equiv), DMAP (1.0 equiv), CH_2_Cl_2_; E2: pyridine-3-isocyanate (2.0 equiv), Et_3_N (3.0 equiv), DMAP (1.0 equiv), CH_2_Cl_2_; E3: morpholine-4-carbonyl chloride (2.0 equiv), Et_3_N (3.0 equiv), DMAP (1.0 equiv), CH_2_Cl_2_; E4: isoxazole-5-carbonyl chloride (2.0 equiv), pyridine; ^d^Yield over two steps.

## Conclusion

Metathesis is an extremely powerful reaction for diversity-oriented synthesis. It was demonstrated that metathesis substrates could be assembled efficiently from triplets of building blocks. Thereafter, metathesis cascades yielded a diverse range of molecular scaffolds. The diversity of the products was increased through variation of all three of the building blocks used: the initiating, the propagating, and the terminating building block.

The overall approach was facilitated by fluorous tagging of the initiating building block, allowing easy purification (by F-SPE) of synthetic intermediates and metathesis products. The presence of a fluorous tag also facilitated the purification of the functionalised products. Evaluation of the biological activity of the final products will be reported in due course.

## Supporting Information

File 1 Experimental and compound characterisation.
