# Supplementary material for: Synthesis of skeletally diverse alkaloid-like molecules: exploitation of metathesis substrates assembled from triplets of building blocks
Source: Beilstein J Org Chem. 2013 Apr 22;9:775–85. doi: 10.3762/bjoc.9.88 (PMC3678641; doi:10.3762/bjoc.9.88)
Supplement: File 1 — Experimental and compound characterisation. [file Beilstein_J_Org_Chem-09-775-s001.pdf]

Supporting Information

for

Synthesis of skeletally diverse alkaloid-like molecules: exploitation of metathesis substrates assembled from triplets of building blocks

Sushil K. Maurya[1,2], Mark Dow[1,2], Stuart Warriner[1,2] and Adam Nelson*[1,2]

Address: [1]School of Chemistry, University of Leeds, Leeds, LS2 9JT, UK and [2]Astbury Centre for Structural Molecular Biology, University of Leeds, Leeds, LS2 9JT, UK

Email: Adam Nelson - a.s.nelson@leeds.ac.uk

* Corresponding author

Experimental and compound characterisation

1	General Experimental	S2
2	General Methods	S4
3	Synthesis of Building Blocks	S7
4	Synthesis of Metathesis Substrates	S17
5	Synthesis of Metathesis Products	S32
6	Synthesis of Final Products	S43
7	Chiral HPLC Analysis of the Amide **S5**	S64
8	References	S65

1 General Experimental

All nonaqueous reactions were carried out under nitrogen. Water-sensitive reactions were performed in oven- or flame-dried glassware cooled under nitrogen before use. Solvents were distilled before use when necessary and possible according to scale. Tetrahydrofuran was either freshly distilled from sodium, using benzophenone as a self-indicator or used as supplied from Sigma–Aldrich. CH_2Cl_2 was either freshly distilled from calcium hydride or used as supplied from Sigma–Aldrich. All other solvents and reagents were of analytical grade and used as supplied. Commercially available starting materials were obtained from Sigma–Aldrich, Lancaster or Alfa Aesar. HG-II refers to Hoveyda–Grubbs second generation catalyst. Ether refers to diethyl ether and petrol refers to petroleum spirit (b.p. 40–60 °C) unless otherwise stated. Solvents were removed under reduced pressure using a Büchi rotary evaporator and a Vacuubrand diaphragm pump.

Flash column chromatography was carried out using silica (35–70 µm particles). Thin layer chromatography was carried out on commercially available precoated plates (Merck silica Kieselgel $60F_{254}$). Analytical LC–MS was performed using either a Waters X-Terra chiral column (MS C18, 5 µm, 50 × 4.6 mm) with a Waters 2525 pump, Waters 2996 photodiode array detector and a Waters Micromass ZQ mass spectrometer as the detector; or an Agilent 1200 series LC system comprising of a Bruker HCT Ultra ion trap mass spectrometer, a high vacuum degasser, a binary pump, a high performance autosampler and micro well plate autosampler, an autosampler thermostat, a thermostated column compartment and a diode array detector. The system used two solvent systems: $MeCN/H_2O$ + 0.1% formic acid with a Phenomenex Luna C18 50 × 2 mm 5 micron column or $MeCN/H_2O$ with a Phenomenex Luna C18 50 × 2 mm 5 micron column. Chiral analytical HPLC was performed using a Daicel Chemical Industries LTD AD-H Chiralpak chiral column (5 µm, 150 × 4.6 mm) eluting with 5% IPA/*n*-hexane.

Proton and carbon NMR spectra were recorded on a Bruker Advance DPX 300, Advance 500 or DRX500 spectrophotometer using an internal deuterium lock. Carbon NMR spectra were recorded with composite pulse decoupling using the waltz 16 pulse sequence. DEPT, COSY, HMQC and HMBC pulse sequences were routinely used to aid the assignment of spectra. Chemical shifts are quoted in parts per million downfield of tetramethylsilane and values of coupling constants (J) are given in Hz. NMR spectra were recorded at 300 K unless otherwise stated.

Melting points were determined on a Reichert hot stage apparatus and are uncorrected. Infrared spectra were recorded on a Perkin Elmer spectrum one FT-IR infrared spectrophotometer and signals were referenced to the polystyrene 1601 cm^{-1} absorption. Nominal mass spectrometry was routinely performed on a Waters-Micromass ZMD spectrometer using electrospray (+) ionization. Nominal and accurate mass spectrometry using electrospray ionization was carried out by staff in the School of Chemistry at the University of Leeds, using either a Micromass LCT-KA111 or Bruker MicroTOF mass spectrometer. Field desorption ionisation mass spectra were acquired on a Waters-Micromass GCT premier spectrometer equipped with a Linden

LIFDI probe. Optical activity measurements were recorded at room temperature on an AA-1000 polarimeter; units for $[\alpha]_D$ are 10^{-1} deg cm^2 g^{-1} and are omitted.

The synthesis of the building blocks **8**, **9**, **10**, **11** and **12b** has previously been reported [S1].

2 General methods

General method for purification by fluorous-solid-phase extraction (F-SPE)

The crude reaction mixture was loaded onto the column with the minimal amount of CH_2Cl_2, MeOH or DMF. The non-fluorous-tagged compounds were eluted with 80:20 MeOH–water (80:20) until elution was deemed complete by TLC. The fluorous-tagged compounds were then eluted using MeOH. The purity of products that were not subsequently purified using another technique was determined by 500 MHz ^{1}H NMR spectroscopy.

A: Fukuyama–Mitsunobu reactions

Method A1: Diethyl azodicarboxylate (4 equiv) was added dropwise to a stirred solution of the fluorous-tagged sulfonamide (1 equiv), triphenylphosphine (4 equiv) and the alcohol (4 equiv) in CH_2Cl_2 (ca. 0.01 M) at 0 °C. The reaction mixture was allowed to warm to room temperature and was stirred for a specified time. The solvent was removed under reduced pressure to give a crude product which was purified by F-SPE.

Method A2: Diethyl azodicarboxylate (2 equiv) was added dropwise to a stirred solution of the fluorous-tagged sulfonamide (1 equiv), triphenylphosphine (2 equiv) and the alcohol (4 equiv) in CH_2Cl_2 (ca. 0.01 M) at 0 °C. The reaction mixture was allowed to warm to room temperature and was stirred for a specified time. The solvent was removed under reduced pressure to give a crude product, which was purified by F-SPE.

Method A3: Diethyl azodicarboxylate (2 equiv) was added dropwise to a stirred solution of the fluorous-tagged sulfonamide (1 equiv), triphenylphosphine (2 equiv) and the alcohol (4 equiv) in THF (ca. 0.01 M) at 0 °C. The reaction mixture was allowed to warm to room temperature and was stirred for a specified time. The solvent was removed under reduced pressure to give a crude product, which was purified by F-SPE.

Method A4: Diethyl azodicarboxylate (2 equiv) was added dropwise to a stirred solution of the fluorous-tagged alcohols (1 equiv), triphenylphosphine (2 equiv) and the sulfonamides/amines (4 equiv) in CH_2Cl_2 (ca. 0.01 M) at 0 °C. The reaction mixture was allowed to warm to room temperature and was stirred for a specified time. The solvent was removed under reduced pressure to give a crude product, which was purified by F-SPE.

Method A5: Diethyl azodicarboxylate (4 equiv) was added to a stirred solution of the fluorous-tagged alcohol (1equiv), triphenylphosphine (4 equiv) and the sulfonamide/amines (4 equiv) in THF (ca. 0.01 M) at 0 °C. The reaction mixture was allowed to warm to room temperature and was stirred for a specified time. The solvent was removed under reduced pressure to give a crude product, which was purified by F-SPE.

Method A6: Diethyl azodicarboxylate (2 equiv) was added to a stirred solution of the fluorous-tagged alcohol (1 equiv), triphenylphosphine (2 equiv) and the sulfonamide/amines (4 equiv) in THF (ca. 0.01 M) at

0 °C. The reaction mixture was allowed to warm to room temperature and was stirred for a specified time. The solvent was removed under reduced pressure to give a crude product, which was purified by F-SPE.

General procedure for the deacetylation of fluorous-tagged intermediates: The substrate was dissolved in a saturated solution of ammonia in methanol (ca. 0.025 M) and stirred at room temperature until the reaction was deemed complete by TLC analysis. The solvent was removed under reduced pressure to give a crude product, which was used without purification.

B: Metathesis reactions

Method B1: The catalyst HG-II (5 mol %) was added to a stirred solution of the metathesis precursor in dichloromethane (ca. 1 mM) under reflux at 50 °C, with addition of further 5 mol % portions of HG-II when the reaction of the starting material appeared to have stalled (by TLC analysis). The reaction mixture was allowed to cool to room temperature, and triethylamine (86 equiv) and tris(hydroxymethyl)phosphine (86 equiv) were added. The reaction mixture was stirred for 15 min, silica (~10 g per mmol of substrate) was added, and the suspension was stirred for 15 min. The suspension was filtered through Celite, washing with ethyl acetate, and the filtrate was concentrated under reduced pressure to give a crude product.

Method B2: The catalyst HG-II (5 mol %) was added to a stirred solution of the metathesis precursor in refluxing *tert*-butyl methyl ether (MTBE) (ca. 1 mM) at 50 °C with the addition of further 5 mol % portions of HG-II when the reaction of the starting material appeared to have stalled (by TLC analysis). The reaction mixture was allowed to cool to room temperature and triethylamine (86 equiv) and tris(hydroxymethyl)phosphine (86 equiv) were added. The reaction mixture was stirred for 15 min, silica (~10 g per mmol of substrate) was added, and the suspension was stirred for 15 min. The suspension was filtered through Celite, washing with ethyl acetate, and the filtrate was concentrated under reduced pressure to give a crude product.

C: Removal of *o*-nitrophenylsulfonyl protecting group

Method C1: The fluorous-tagged sulfonamide (1 equiv) and K_2CO_3 (3 equiv) were dissolved in anhydrous DMF (ca. 0.1 M) and cooled to 0 °C. Thiophenol (1.2 equiv) was added dropwise and the reaction mixture was stirred at room temperature for specified time. The mixture was purified directly by F–SPE.

Method C2: The fluorous-tagged sulfonamide (1 equiv) and K_2CO_3 (6 equiv) were dissolved in anhydrous DMF (ca. 0.1 M) and cooled to 0 °C. Thiophenol (2.4 equiv) was added dropwise and the reaction mixture was stirred at room temperature for specified time. The mixture was purified by F–SPE.

D: Desilylation

Method D: The fluorous-tagged silyl ether (0.030 mmol) was dissolved in CH_3CN (0.5 mL) and CH_2Cl_2 (0.4 mL), and HF (50% aq, 0.2 mL/ 50 mg of substrate) was added. The reaction mixture was stirred for 3 h, concentrated under a flow of N_2 to give a crude product.

E: Derivitisation reactions

Method E1: Isoxazole-5-carbonyl chloride (2 equiv) was added dropwise to a solution of the fluorous-tagged amine (1 equiv), Et_3N (5 equiv) and DMAP (1 equiv) in CH_2Cl_2 (0.1 M) at 0 °C. The reaction mixture was stirred for 3 h at room temperature, concentrated under a flow of N_2 and purified to give a crude product.

Method E2: Pyridine-3-isocyanate (2 equiv) was added to the fluorous-tagged amine (1 equiv) dissolved in CH_2Cl_2 (0.02 M) at 0 °C. The reaction mixture was stirred for 3 h at room temperature, and concentrated under a flow of N_2 to give a crude product.

Method E3: Morpholine 4-carbonyl chloride (2 equiv) was added dropwise to a solution of the fluorous-tagged amine (1 equiv), Et_3N (5 equiv) and DMAP (1 equiv) in CH_2Cl_2 (0.1 M) at 0 °C. The reaction mixture was stirred for 3 h at room temperature, concentrated under a flow of N_2 and purified to give a crude product.

Method E4: Isoxazole-5-carbonyl chloride (2 equiv) was added dropwise to a solution of the fluorous-tagged amine (1 equiv), in anhydrous pyridine (0.1 M) at 0 °C. The reaction mixture was stirred for 3 h at room temperature, and concentrated under a flow of N_2 to give a crude product.

3 Synthesis of Building blocks

4-(Hydroxymethyl)benzaldehyde (S1) [S2]

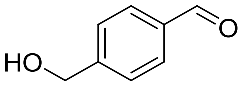

A solution of $NaBH_4$ (3.0 gm, 79.3 mmol) in anhydrous MeOH (5 mL) cooled at 0 °C was added at once to a solution of terephthaldehyde (15.0 gm, 112.0 mmol) in anhydrous THF (100 mL) cooled at 0 °C, and the reaction mixture was stirred at room temperature for 12 h. Solvent was removed and the residue was taken up in EtOAc (200 mL). The solution was washed with water (2 × 100 mL) and brine, dried ($MgSO_4$), and concentrated in vacuo to give a crude product, which was purified by flash column chromatography (gradient elution: 10:90 → 40:60, ethyl acetate–petrol) to give **S1** (9.9 g, 65%) as a amorphous colourless solid; R_f 0.38 (50:50 EtOAc–petrol); ^{1}H (500 MHz, $CDCl_3$) δ 9.95 (1H, s, C*H*O), 7.85 (2H, d, *J* 7.7, Ar), 7.49 (2H, d, *J* 7.7, Ar), 4.76 (2H, s, *CH*$_2$-Ar), 2.59 (1H, s, O*H*); ^{13}C (75 MHz, $CDCl_3$) δ 192.1 (C=O), 147.9 (Ar), 135.9 (Ar), 130.1 (Ar), 127.1 (Ar), 64.7 (*CH*$_2$-Ar); ν_{max}/cm^{-1} (film) 3348, 2909, 2851, 2748, 1690, 1609; m/z (ES) [M+] 136.1 (100%, M+); HRMS Found: 136.0529, $C_8H_8O_2$ requires 136.0524.

4-((*tert*-Butyldimethylsilyloxy)methyl)benzaldehyde (S2) [S3]

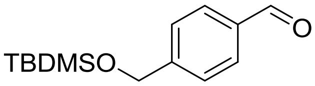

Imidazole (9.97 g, 146.4 mmol) was added to a CH_2Cl_2 (50 mL) solution of the benzylic alcohol **S1** and was stirred at r.t. for 30 min. To this solution was added TBDMSCl (13.3 g, 88.2 mmol) portionwise and the reaction mixture was stirred 24 h at r.t., washed with water and extracted with chloroform, dried ($MgSO_4$), concentrated in vacuo and purified by flash column chromatography (gradient elution: 10:90 → 30:70, ethyl acetate–petrol) to give silyl ether **S2** (15.4 g, 84%) as colourless amorphous solid. R_f 0.68 (30:70, EtOAc:petrol); ^{1}H (500 MHz, $CDCl_3$) δ 9.99 (1H, s, C*H*O), 7.85 (2H, d, *J* 8.0, Ar), 7.48 (2H, d, *J* 8.0, Ar), 4.82 (2H, s, *CH*$_2$-Ar), 0.96 (9H, s, *t*-butyl), 0.12 (6H, s, *CH*$_3$-TBS); ^{13}C (75 MHz; $CDCl_3$) δ 192.2 (*C*-1), 148.8 (*C*-5), 135.5 (*C*-2), 130.0 (*C*-4,6), 126.3 (*C*-3,7), 64.6 (*C*-8), 26.0 (*t*-butyl), 18.5 (*t*-butyl), -5.2 (*CH*$_3$-TBS); ν_{max}/cm^{-1} (film) 2930, 2858, 1694, 1611, 1579; m/z (ES) [M+H] 251.1 (100%, M+H); HRMS Found: 251.1462, $C_{14}H_{23}O_2Si_1$ requires 251.1460.

(*E*)-Ethyl 3-(4-((*tert*-butyldimethylsilyloxy)methyl)phenyl)acrylate (S3)

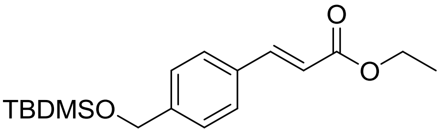

NaH (3.5 g, 145.6 mmol) was added to the solution of ethyl 2-(dimethoxyphosphoryl)acetate dissolved in anhydrous THF cooled at 0 °C portionwise. The reaction mixture was cooled to −78 °C and the aldehyde **S2** dissolved in THF was added dropwise and was stirred for 1 h. The reaction mixture was warmed to r.t. and

stirred for another 2 h. The reaction mixture was quenched with a saturated solution of NH_4Cl, the aq. phase was extracted with EtOAc, dried ($MgSO_4$), concentrated in vacuo, and purified using flash column chromatography to afford ester **S3** (14.1 g, 79%) as syrup, R_f 0.58 (20:80, EtOAc:petrol); ^{1}H (500 MHz, $CDCl_3$) δ 7.68 (1H, d, *J* 15.8, 3-H), 7.49 (2H, d, *J* 8.1, Ar), 7.33 (2H, d, *J* 8.1, Ar), 6.41 (1H, d, *J* 15.8, 2-H), 4.75 (2H, s, CH_2-Ar), 4.26 (2H, q, *J* 7.3, 14.5, CH_2-ethyl), 1.34 (3H, t, *J* 6.8, CH_3-ethyl), 0.95 (9H, s, CH_3-TBS), 0.10 (6H, s, CH_3-TBS); ^{13}C ($CDCl_3$, 75 MHz) δ 167.3 (C=O), 144.6 (C-3), 144.1 (Ar), 133.2 (Ar), 128.2 (Ar), 126.5 (Ar), 117.8 (C-2), 64.7 (CH_2-Ar), 60.6 (CH_2-ethyl), 26.1 (*t*-butyl), 18.5 (*t*-butyl), 14.5 (CH_3-ethyl), -5.1 (CH_3-TBS); ν_{max}/cm^{-1} (film) 3368, 2955, 2930, 2857, 1710, 1637; m/z (ES) [M+H] 321.2 (100%, M+H); HRMS Found: 321.1881, $C_{18}H_{29}O_3Si_1$ requires 321.1880.

(*E*)-3-(4-((*tert*-Butyldimethylsilyloxy)methyl)phenyl)prop-2-en-1-ol (14)

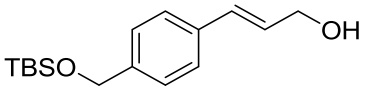

Di*iso*butylaluminium hydride (1M solution in hexane, 110 mL, 109.4 mmol) was added dropwise to a solution of the ester **S3** (14.0 g, 43.7 mmol) in THF (100 mL) at –78 ºC and the reaction mixture was stirred at –78 ºC for 1 h and at room temperature for 17 h. The mixture was cooled to 0 ºC and a saturated solution of sodium potassium tartrate (100 mL) was added and was stirred at r.t. The organic layers were washed with water, dried ($MgSO_4$), concentrated in vacuo and purified by flash column chromatography (gradient elution: 10:90 → 40:60, ethyl acetate–petrol) to afford the alcohol **14** (8.9 g, 75%) as a colourless amorphous solid; R_f 0.28 (20:80, EtOAc–petrol); ^{1}H NMR ($CDCl_3$, 500 MHz) δ 7.36 (2H, d, *J* 8.1, Ar), 7.27 (2H, d, *J* 8.1, Ar), 6.60 (1H, d, *J* 15.8, 3-H), 6.30 (1H, dt, *J* 15.8, 5.9, 2-H), 4.73 (2H, s, CH_2-Ar), 4.32 (2H, d, *J* 5.9, 1-CH_2), 0.94 (9H, s, CH_3 *t*-butyl), 0.10 (6H, s, CH_3 TBS); ^{13}C ($CDCl_3$, 75 MHz) δ 141.2 (Ar), 135.2 (Ar), 131.2 (C-3), 128.1 (C-2), 126.5 (Ar), 126.4 (Ar), 64.9 (CH_2-Ar), 63.9 (1-CH_2), 26.0 (*t*-butyl), 18.6 (*t*-butyl), -5.1 (CH_3-TBS); ν_{max}/cm^{-1} (film) 3542, 3413, 3029, 2928, 1917, 1659; m/z (ES) [M+Na] 301.2 (100%, M+Na); HRMS Found: 301.1588, $C_{16}H_{26}Na_1O_2Si_1$ requires 301.1594.

(*E*)-3-(4-((*tert*-Butyldimethylsilyloxy)methyl)phenyl)allyl methyl carbonate (15)

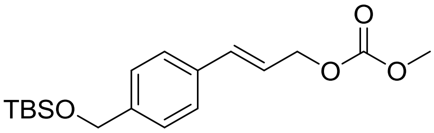

A solution of alcohol **14** in anhydrous CH_2Cl_2 (100 mL) cooled to 0 °C was treated with DMAP (11.1 g, 91.7 mmol) and was stirred for 10 min and methyl chloroformate dissolved in CH_2Cl_2 (50 mL) was added dropwise at 0 °C. The reaction mixture was stirred at r.t. overnight and was quenched with water, extracted with ethyl acetate, dried ($MgSO_4$), concentrated in vacuo, and purified by flash column chromatography to afford the carbonate **15** (9.5 g, 93%) as colourless amorphous solid, R_f 0.68 (30:70, EtOAc–petrol); ^{1}H ($CDCl_3$, 500 MHz) δ 7.36 (2H, d, *J* 8.1, Ar), 7.28 (2H, d, *J* 8.1, Ar), 6.68 (1H, d, *J* 15.8, 3-H), 6.28 (1H, dt, *J* 6.4, 15.8, 2-H), 4.78 (2H, d, *J* 6.4, 1-CH_2), 4.73 (2H, s, CH_2-Ar), 3.80 (3H, s, OCH_3), 1.58 (9H, s, *t*-butyl), 0.94 (6H, s, CH_3-TBS); ^{13}C ($CDCl_3$, 75 MHz) δ 155.8 (C=O), 141.8 (Ar), 134.9 (C-3), 134.8 (Ar), 126.7

(Ar), 126.4 (Ar), 122.0 (C-2), 68.7 (C-1), 64.8 (*CH*$_2$-Ar), 55.0 (O*CH3*), 26.1 (*t*-butyl), 18.5 (*t*-butyl), -5.1 (*CH*$_3$-TBS); ν_{max}/cm^{-1} (film) 3012, 2946, 2885, 2188, 1920, 1756; m/z (ES) [M+Na] 359.2 (100%, M+Na); HRMS Found: 359.1651, $C_{18}H_{22}Na_1O_4Si_1$ requires 359.1649.

(*S*)-*N*-(1-(4-((*tert*-Butyldimethylsilyloxy)methyl)phenyl)allyl)-2-nitrobenzenesulfonamide (17)

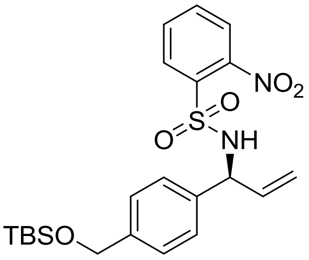

Under argon, 1,5,7-triazabicyclo[4.4.0]dec-5-ene (TBD) (83 mg, 0.60 mmol) was added to a solution of [Ir(cod)Cl]$_2$ (100 mg, 0.15 mmol) and (*S*,*S*,*S*)-(+)-(3,5-dioxa-4-phosphacyclo-hepta[2,1-*a*:3,4-*a'*]dinaphthalene-4-yl)-*N*,*N*-bis(1-phenylethyl)amine (162 mg, 0.30 mmol) in dry THF (2 mL). After stirring for 2 h at r.t., the allylcarbonate **15** (2.5 g, 7.44 mmol) dissolved in THF (10 mL) was added, and the mixture was stirred for 5 min at 50 °C, then the nucleophile NsNH$_2$ (1.8 g, 8.90 mmol) dissolved in THF (10 mL) was added and the reaction mixture was stirred at 50 °C for 12 h, concentrated in vacuo, and the residue obtained was purified flash column chromatography (gradient elution: 10:90 → 30:70, ethyl acetate–petrol) to afford the sulfonamide **17** (1.67 g, 66%), R_f 0.40 (30:70, EtOAc–petrol); $[\alpha]^{D}_{27}$: –88.4 (*c* 0.9, CHCl$_3$); ^{1}H (CDCl$_3$, 500 MHz) δ 7.81 (1H, dd, *J* 1.3, 7.7, nosyl), 7.74 (1H, dd, *J* 1.3, 7.7, nosyl), 7.57 (1H, td, *J* 1.3, 7.7, nosyl), 7.48 (1H, td, *J* 1.3, 7.7, nosyl), 7.09 (4H, m, Ar), 5.93 (1H, ddd, *J* 5.5, 10.2, 16.6, 2-H), 5.82 (1H, d, *J* 9.0, *N-H*), 5.14 (3H, m, 3-H and 1-H$_2$), 4.61 (2H, s, *CH*$_2$-Ar), 0.92 (9H, s, *t*-butyl), 0.07 (6H, s, *CH*$_3$-TBS); ^{13}C (CDCl$_3$, 75 MHz) δ 147.5 (nosyl), 141.5 (Ar), 137.1 (Ar), 136.7 (2-C), 134.8 (nosyl), 133.1 (nosyl), 132.6 (nosyl), 131.1 (nosyl), 127.1 (Ar), 126.3 (Ar), 125.1 (nosyl), 117.5 (1-C), 64.5 (*CH*$_2$-Ar), 60.7 (3-C), 26.0 (*t*-butyl), 18.5 (*t*-butyl), -5.1 (*CH*$_3$-TBS); ν_{max}/cm^{-1} (film) 3354, 2954, 2929, 2857, 1538; m/z (ES) [M+Na] 485.2 (29%, M+Na), 480.2 (71%, M+NH$_4$); HRMS Found: 480.2002, $C_{22}H_{34}N_3O_5S_1Si_1$ requires 480.1983.

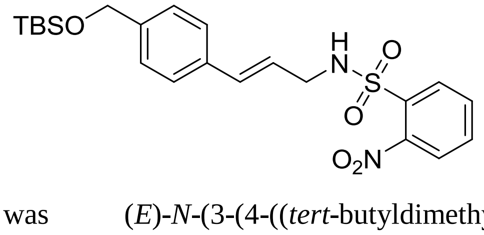

Also obtained was (*E*)-*N*-(3-(4-((*tert*-butyldimethylsilyloxy)methyl)phenyl)allyl)-2-nitrobenzenesulfonamide **16** (0.23 g, 7%), m.p. 76-78 °C; R_f 0.42 (30:70, EtOAc–petrol); ^{1}H (CDCl$_3$, 500 MHz) δ 8.13 (1H, m, nosyl), 7.85 (1H, m, nosyl), 7.73 (2H, m, nosyl), 7.23 (2H, d, *J* 8.6, Ar), 7.17 (2H, d, *J* 8.6, Ar), 6.46 (1H, d, *J* 15.8, 3-H), 5.99 (1H, dt, *J* 15.8, 6.8, 2-H), 5.47 (1H, t, *J* 5.9, *N-H*), 4.70 (2H, s, *CH*$_2$-Ar), 3.93 (2H, t, *J* 5.9, 1-H), 0.94 (9H, s, *t*-butyl), 0.09 (6H, s, CH$_3$-TBS); ^{13}C (CDCl$_3$, 75 MHz) δ 141.7 (Ar), 134.6 (nosyl), 134.4 (Ar), 133.7 (nosyl), 133.6 (C-3), 132.9 (nosyl), 131.4 (nosyl), 126.5 (Ar), 126.4 (Ar), 125.5 (Ar), 122.9 (C-2), 64.8 (CH$_2$-Ar), 46.3 (C-1), 26.1 (*t*-butyl), 18.5 (*t*-butyl), −5.1 (*CH*$_3$-TBS);

ν_{max}/cm^{-1} (film) 3356, 3327, 3091, 2936, 2857, 1917; m/z (ES) [M+NH$_4$] 480.2 (100%, M+NH$_4$); HRMS Found: 480.2002, $C_{22}H_{34}N_3O_5S_1Si_1$ requires 480.1983.

(*S*)-*N*-(1-(4-(Hydroxymethyl)phenyl)allyl)-2-nitrobenzenesulfonamide (18)

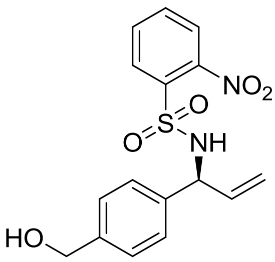

To a solution of the silyl ether **17** (200 mg, 0.43 mmol) in anhydrous THF was added TBAF (0.86 mmol, 1.0 M in THF) dropwise. The reaction mixture was stirred at r.t. until the disappearance of the starting material, then concentrated in vacuo, and the residue obtained was purified by flash column chromatography (gradient elution: 20:80 → 50:50, ethyl acetate–petrol) to afford the alcohol **18** (148 mg, 98%), m.p. 134-136 °C; R_f 0.33 (70:30, EtOAc–petrol); ^{1}H (CD$_3$OD, 500 MHz) δ 7.83 (1H, dd, *J* 1.3, 8.1, nosyl), 7.72 (1H, dd, *J* 1.3, 8.1, nosyl), 7.65 (1H, td, *J* 1.3, 8.1, nosyl), 7.57 (1H, td, *J* 1.3, 7.7, nosyl), 7.18 (4H, m, Ar), 6.00-5.93 (1H, m, 2-H), 5.08 (3H, m, 3-H, 1-*CH$_2$*), 4.51 (2H, s, *CH$_2$*-Ar); ^{13}C (CD$_3$OD, 75 MHz) δ 149.2 (nosyl), 142.2 (Ar), 139.9 (nosyl), 138.7 (C-2), 135.4 (Ar), 134.6 (nosyl), 133.2 (nosyl), 131.7 (nosyl), 128.3 (Ar), 127.9 (Ar), 125.5 (nosyl), 116.9 (C-1), 64.7 (*CH$_2$*-Ar), 61.5 (C-3); ν_{max}/cm^{-1} (film) 3563, 3180, 3095, 2934, 2878, 2401, 1640; m/z (ES) [M+NH$_4$] 366.1 (100%, M+NH$_4$); HRMS Found: 366.1129, $C_{16}H_{20}N_3O_5S_1$ requires 366.1118.

(*S*)-*N*-(1-(4-(((3,3,4,4,5,5,6,6,7,7,8,8,9,9,10,10,10-Heptadecafluorodecyl)diisopropylsilyloxy)methyl)-phenyl)allyl)-2-nitrobenzenesulfonamide (6b)

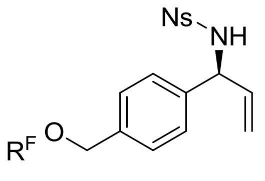

N-Bromosuccinimide (107 mg, 0.60 mmol) was added to a stirred solution of diisopropyl(3,3,4,4,5,5,6,6,7,7,8,8,9,9,10,10,10-heptadecafluorodecyl)silane (270 mg, 0.48 mmol) in dichloromethane (5 mL) at 0 °C. After the reaction mixture was stirred at 0 °C for 30 min, a solution of the alcohol **18** (140 mg, 0.40 mmol) and imidazole (33 mg, 0.48 mmol) in CH_2Cl_2 (5 mL) was added dropwise. The reaction mixture was warmed to room temperature and stirred for 16 h, then the reaction was quenched by the addition of methanol (5 mL) and concentrated in vacuo, and the residue obtained was purified by flash column chromatography (gradient elution: 0:100 → 10:90, ethyl acetate–petrol) to afford the silyl ether **6b** (320 mg, 88%) as a colorless oil, R_f 0.61 (30:70, EtOAc–petrol); ^{1}H (CDCl$_3$, 500 MHz) δ 7.84 (1H, dd, *J* 1.3, 7.7, nosyl), 7.75 (1H, dd, *J* 1.3, 7.7, nosyl), 7.57 (1H, td, *J* 1.3, 7.7, nosyl), 7.50 (1H, td, *J* 1.3, 7.7, nosyl), 7.12 (4H, s, Ar), 5.91 (1H, ddd, *J* 5.9, 10.3, 16.7, 2-H), 5.81 (1H, d, *J* 5.9, *N-H*), 5.14 (3H, m, 3-H, 1-*CH$_2$*), 4.68 (2H, s, *CH$_2$*-Ar), 2.09 (2H, m, *C-H* isopropyl), 1.06 (14H, m, 2'-*CH$_2$*, *CH$_3$* isopropyl), 0.89 (2H,

m, 1'-CH_2); ^{13}C ($CDCl_3$, 75 MHz) δ 147.9 (nosyl), 140.9 (Ar), 137.6 (Ar), 136.7 (C-2), 134.9 (nosyl), 133.2 (nosyl), 132.7 (nosyl), 131.2 (nosyl), 127.4 (Ar), 126.2 (Ar), 125.3 (nosyl), 117.6 (C-1), 64.8 (CH_2-Ar), 60.8 (C-3), 25.6 (2'-C), 17.8 (CH_3 isopropyl), 17.7 (CH_3 isopropyl), 12.6 (*C-H* isopropyl), 0.02 (1'-C); ν_{max}/cm^{-1} (film) 3359, 2947, 2869, 1694, 1643; m/z (ES) [M+NH_4] 926.2 (100%, M+NH_4); HRMS Found: 926.1940, $C_{32}H_{37}F_{17}N_3O_5S_1Si_1$ requires 926.1946.

(*S*)-1-(4-(((3,3,4,4,5,5,6,6,7,7,8,8,9,9,10,10,10-Heptadecafluorodecyl)diisopropyl-silyloxy)methyl)phenyl)prop-2-en-1-amine (19)

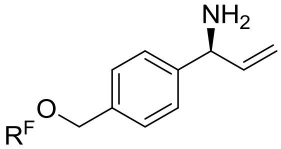

The fluorous-tagged sulfonamide **6b** (8.08 g, 8.89 mmol) and K_2CO_3 (3.8 g, 26.5 mmol) were dissolved in anhydrous DMF (20 mL, ca. 0.1 M) and cooled to 0 °C. Thiophenol (1.20 mL, 11.0 mmol) was added dropwise and the reaction mixture was stirred at r.t. for a specified time. The mixture was purified directly by F–SPE, eluting with 80:20 MeOH–H_2O then with MeOH to furnish the *amine* **19** (5.7 g, 89%), R_f 0.25 (80:20, EtOAc:petrol); δ_H (500 MHz; $CDCl_3$) 7.30 (4H, q, *J* 15.8, 8.1, Ar), 6.01 (1H, m, 3-H), 5.23 (1H, d, *J* 17.5, alkene-CH_2), 5.10 (1H, d, *J* 10.2, alkene-CH_2-H_A), 4.78 (2H, s, benzylic-CH_2), 4.51 (1H, d, *J* 5.5, 2-H), 2.09 (2H, m, tag-CH_2), 1.58 (2H, s, NH_2), 1.08 (14H, m, tag-CH, methyl), 0.89 (2H, m, tag-CH_2); δ_C (75 MHz; $CDCl_3$) 143.8 (Ar), 142.5 (C-3), 139.9 (Ar), 126.9 (Ar), 126.5 (Ar), 114.0 (alkene-CH_2), 65.3 (benzilic-CH_2), 58.5 (C-2), 25.7 (tag-CH_2), 17.9 (tag-methyl), 17.8 (tag-methyl), 12.7 (tag-CH), 0.2 (tag-CH_2); *m/z* (ES) [M+H] 724.2 (100%, M+H); HRMS Found: 724.1875, $C_{26}H_{31}F_{17}N_1O_1Si_1$ requires 724.1898.

(*S*)-1,1,1-trifluoro-*N*-(1-(4-(((3,3,4,4,5,5,6,6,7,7,8,8,9,9,10,10,10-Heptadecafluorodecy l)diisopropylsilyloxy)methyl)phenyl)allyl)methanesulfonamide (6a)

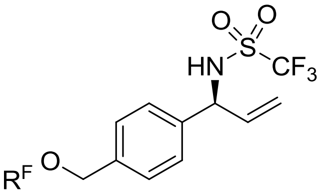

Triflic anhydride (1.6 mL, 9.5 mmol) was added dropwise to a stirred solution of the amine **19** (5.7 g, 7.88 mmol) and triethylamine (2.9 mL, 19.8 mmol) in dichloromethane (20 mL) at 0 °C. After it was stirred at 0 °C for 1 h, the reaction mixture was warmed to room temperature and stirred for 2 h. After the reaction was quenched by the addition of water (12 mL), the reaction mixture was extracted with EtOAc (3 × 50 mL), and the combined organic fractions were washed with a saturated aqueous brine solution (30 mL). After removal of solvent under reduced pressure, the crude product was purified by column chromatography, eluting with 5:95→10:90 petrol–EtOAc, to afford the sulfonamide **6a** (3.85 g, 57%) as a pale yellow oil, R_f 0.54 (10:90, EtOAc:petrol); δ_H (500 MHz; $CDCl_3$) 7.35 (2H, d, *J* 8.1, Ar), 7.27 (2H, d, *J* 8.1, Ar), 6.03 (1H, m, 3-H), 5.37 (1H, dd, *J* 10.2, 1.2, alkene-CH_2-H_A), 5.33 (1H, dd, *J* 17.1, 1.2, alkene-CH_2-H_B), 5.24 (1H, d, *J* 5.6, 5-H), 4.80 (2H, s, benzylic-CH_2), 2.09 (2H, m, tag-CH_2), 1.09 (14H, m, tag-CH, methyl), 0.90 (2H, m, tag-CH_2);

δ_C (75 MHz; $CDCl_3$) 141.8 (Ar), 137.6 (C-3), 136.2 (Ar), 127.3 (Ar), 126.9 (Ar), 116.9 (alkene-CH_2), 65.0 (benzilic-CH_2), 61.0 (C-2), 25.7 (tag-CH_2), 17.8 (tag-methyl), 17.7 (tag-methyl), 12.7 (tag-CH), 0.1 (tag-CH_2); m/z (ES) [M+NH_4] 873.2 (100%, M+NH_4); HRMS Found: 878.1221, $C_{27}H_{29}F_{20}N_1Na_1O_3S_1Si_1$ requires 878.1210.

_N_-[1-[(_tert_-Butyldimethylsilyl)oxy]pent-4-en-2-yl}-2-methylpropane-2-sulfinamide ((_S_s,_R_c)-22)

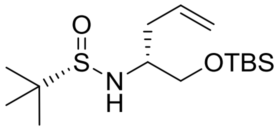

To a solution of (_S_)-_N_-[(1_E_)-2-[(_tert_-butyldimethylsilyl)oxy]ethylidene]-2-methylpropane-2-sulfinamide [S4] (0.9 g, 3.6 mmol) in CH_2Cl_2 (30 mL) at –78 °C, allylmagnesium bromide (7.5 mL of a 1M solution in ether, 7.5 mmol) was added dropwise. After 1 h the reaction was stirred at 0 °C for 4 h and then allowed to reach room temperature. After 16 h the reaction was cooled with an ice-bath and sat. aqueous NH_4Cl was added dropwise; after 2 h the reaction was concentrated in vacuo to half volume and extracted into ethyl acetate (3 × 50 mL). The organic layers were dried over Na_2SO_4, filtered and concentrated in vacuo. Column chromatography, eluting with petrol–EtOAc (80:20) gave the sulfinamide (S_s,R_c)**-22** (810 mg, 2.54 mmol, 70%) as a colourless oil and a single diastereomer; R_f 0.31 (petrol–EtOAc, 80:20); $[\alpha]^D_{23.4}$: +57.6 (_c_ 1.01, $CHCl_3$); δ_H (500 MHz; $CDCl_3$) 5.75 (1H, ddt, _J_ 7.2, 10.3 and 17.5, 4-H), 5.11 (1H, d, _J_ 7.2, 5-H), 5.07 (1H, s, 5-H), 3.61 (1H, dd, _J_ 4.3 and 10.3, 3-H_a), 3.47 (1H, dd, _J_ 5.3 and 10.3), 3.46-3.43 (1H, m, N-H), 3.33-3.24 (1H, m, 2-H), 2.51-2.29 (2H, m, 1-H), 1.14 (9H, s, ^{t}Bu), 0.84 (9H, s, $SiC(CH_3)_3$), 0.00 (6H, s, $Si(CH_3)_2$); δ_C (75 MHz; $CDCl_3$) 134.8 (4-C), 118.9 (5-C), 65.6 (1-C), 56.8 (2-C), 56.3 ($SC(CH_3)_3$), 37.5 (3-C), 26.3 (^{t}Bu), 22.9 ($SiC(CH_3)_3$), 18.6 ($SiC(CH_3)_3$), 0.41 ($Si(CH_3)_2$); ν_{max}/cm^{-1} (film): 2954, 2928, 2857, 1252, 1099, 1051, 855 and 775; _m/z_ (ES+) 320.2 (100%, $[M+H]^+$).

Also obtained was the sulfinamide (S_s,S_c)**-22** (196 mg, 0.61 mmol, 17%); $[\alpha]^D_{23.4}$: +26.9 (_c_ 1.0, $CHCl_3$); R_f 0.48 (80:20, petrol–EtOAc); δ_H (500 MHz; $CDCl_3$) 5.77 (1H, dddd, _J_ 17.0, 10.4, 7.5 and 6.7, 4-H), 5.07 (1H, d , _J_ 17, 5-H), 5.06 (1H, d, _J_ 10.4, 5-H), 3.78 (1H, d, _J_ 6.7, NH), 3.74 (1H, dd , _J_ 9.9 and 4.6, 1-H), 3.60 (1H, dd , _J_ 9.9 and 5.1, 1-H), 3.38 (1H, qt, _J_ 6.4 and 4.8, 2-H), 2.40-2.21 (2H, m, 3-H); 1.21 (9H, s, tBu), 0.90 (9H, s, $SiC(CH_3)_3$), 0.07 (3H, s, $SiCH_3$), 0.06 (3H, s, $SiCH_3$); δ_C (75 MHz; $CDCl_3$) 134.5 (4-C), 117.6 (5-C), 65.4 (1-C), 56.4 (2-C), 55.7 (SO^{t}Bu), 36.8 (3-C), 25.8 (SO^{t}Bu), 22.7 ($SiC(CH_3)_3$), 18.2 ($SiC(CH_3)_3$), -5.2 ($SiCH_3$), -5.3 ($SiCH_3$); ν_{max}/cm^{-1} (film) 3312, 2956, 2930, 2858, 1642, 1472, 1390, 1364 and 1324; _m/z_ (ES+) 320.1 (20%, $[M+H]^+$). See reference [S4] for the previous description of this reaction in which the configuration of the product is incorrectly assigned.

(2_R_)-2-Aminopent-4-enol hydrochloride (23)

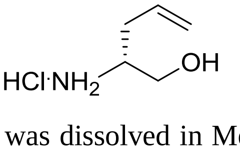

The sulfinamide (S_s,R_c)**-22** (4.0 g, 10 mmol) was dissolved in MeOH (40 mL), and 4 N HCl in 1,4-dioxane (20 mL) was added dropwise at 0 °C for 1 h and then room temperature for 4 h. The reaction was

concentrated in vacuo to give a pale yellow solid. The solid was slurried in Et_2O (20 mL) and filtered to give the amine hydrochloride **23** (1.30 g, 9.5 mmol) as a colourless crystalline solid, $[\alpha]^{D}_{23.4}$: −10.3 (*c* 0.7, MeOH); δ_H (500 MHz; MeOD) 5.84 (1H, ddt, *J* 7.1, 10.2 and 17.2), 5.31-5.20 (2H, m, 5-H_a and 5-H_b), 3.78 (1H, dd, *J* 3.8 and 11.6, 3-H_a), 3.58 (1H, dd, *J* 7.1 and 11.6, 3-H_b), 3.31-3.24 (1H, m, 2-H), 2.5-2.34 (2H, m, 1-H_{ab}); δ_C (75 MHz; MeOD) 131.9 (4-C), 118.8 (5-C), 60.5 (1-C), 52.5 (2-C) , 33.5 (3-C); ν_{max}/cm^{-1} (solid): 2472, 2071, 1121 and 972.

(2*R*)-1-Hydroxy-*S*-(2-nitrophenyl)pent-4-ene-2-sulfonamide (24)

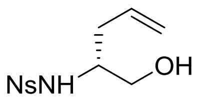

The amine hydrochloride **23** (1.6 g, 11.6 mmol) was dissolved in CH_2Cl_2 (50 mL), triethylamine (2.93 g, 29 mmol) was added and the reaction cooled to 0 °C. 2-Nitrobenzene sulfonyl chloride (2.58 g, 11.6 mmol) was added in one portion; after 1 h the ice bath was removed and the reaction was stirred at room temperature. After 16 h the reaction was poured into water (50 mL), separated and washed with HCl (0.5M, 50 mL), 10% $NaHCO_3$ (50 mL) and brine (100 mL). The organic layers were dried over $MgSO_4$, filtered and concentrated in vacuo to give the *sulfonamide* **24** (3.15 g, 11.1 mmol, 95%) as a pale yellow viscous oil, which was not purified, R_f 0.71 (80:20, EtOAc–petrol); $[\alpha]^{D}_{23.4}$: −5.1 (*c* 0.3, $CHCl_3$); δ_H (500 MHz; $CDCl_3$) 8.17-8.13 (1H, m, nosyl 3-H), 7.90-7.85 (1H, m, nosyl 6-H), 7.79-7.72 (2H, m, nosyl 4 and 5-H), 5.54 (1H, ddt, *J* 7.2, 10.0 and 17.2, 4-H), 5.01 (1H, *J* 17.0, 5-H_a), 4.92 (1H, *J* 10.0, 5-H_b), 3.66-3.53 (3H, m, 1-H_{ab} and 2-H), 2.35-2.22 (2H, m, 3-H_{ab}); δ_C (75 MHz; $CDCl_3$) 147.7 (nosyl 2-C), 134.5 (4-C), 133.6 (nosyl 1-C), 132.9 (nosyl 4 and 5-C), 130.7 (nosyl 6-C)), 125.4 (nosyl 3-C), 118.9 (5-C), 64.4 (1-C), 56.2 (2-C), 36.2 (3-C); ν_{max}/cm^{-1} (film) 3334, 1537, 1163 and 593; *m/z* (ES+) 309.1 (100%, $[M+Na]^+$); found 309.0515, $C_{11}H_{14}N_2O_5S$ requires *MNa*, 309.0516.

***N*-[(2*R*)-1-{[(3,3,4,4,5,5,6,6,7,7,8,8,9,9,10,10,10-Heptadecafluorodecyl)bis(propan-2-yl)silyl]oxy}pent-4-en-2-yl]-2-nitrobenzene-1-sulfonamide (7)**

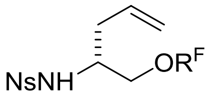

A solution of (1*H*,1*H*,2*H*,2*H*-heptadecafluorodecyl)diisopropylsilane (6.6 g, 11.7 mmol) in CH_2Cl_2 (40.0 mL) was added slowly to a solution of *N*-bromosuccinimide (2.2 g, 12.2 mmol) in CH_2Cl_2 (50 mL) at 0 °C. After 5 min at 0 °C, the reaction was then stirred for 20 min at room temperature. A solution of the sulfonamide **24** (3.15 g, 11.1 mmol) and imidazole (1.0 g, 14.6 mmol) dissolved in CH_2Cl_2 (50 mL) was added dropwise at 0 °C. After 16 h at room temperature the reaction was concentrated in vacuo, dissolved in the petrol–EtOAc (50:50) and filtered through a silica/Celite® plug. The resulting filtrate was concentrated in vacuo, to give the sulfonamide **7** (9.3 g, 11.1 mmol, 99%) as a pale yellow viscous oil, which was not purified further, R_f 0.95 (80:20 EtOAc–petrol); $[\alpha]^{D}_{23.4}$: −2.4 (*c* 1.5, $CHCl_3$); δ_H (500 MHz; $CDCl_3$) 8.14-8.12 (1H, m, nosyl), 7.87-7.84 (1H, m, nosyl), 7.73-7.69 (1H, m, nosyl), 5.65 (1H, d, *J* 10, N-H), 5.61 (1H, ddt, *J* 9.5, 13 and 18, 4-H), 5.03 (1H, d, *J* 18, 5-H), 4.97 (1H, d, *J* 13, 5-H), 3.72-3.69 (1H, m, 3-H_a), 3.61-

3.52 (2H, m, 3-H_b and 2-H), 2.35-2.26 (2H, m, 1-H), 2.15-1.94 (2H, m, 2′-H), 0.98 (14H, s, ^{i}Pr), 0.86-0.75 (2H, m, 1′-H); δ_C (75 MHz; $CDCl_3$) 135.1 (4-C), 133.4 (nosyl 1-C), 133.0 (nosyl 6-C), 132.9 (nosyl 4 or 5-C), 130.6 (nosyl 6-C), 125.4 (nosyl 3-C), 118.8 (5-C), 64.6 (1-C), 55.8 (2-C), 36.2 (3-C), 24.5 (^{i}Pr), 17.4 (^{i}Pr), 12.2 (^{i}Pr), -0.3 (1′-C), *nosyl 2-C missing*; ν_{max}/cm^{-1} (film): 2949, 2870, 1643, 1543, 1275 and 1259; *m/z* (ES+) 864.2 (100%, $[M+NH_4]^+$); found 864.1787, $C_{27}H_{35}F_{17}N_3O_5SSi$ requires *MNH_4*, 864.1790.

2-Aminopent-4-enol (S4)

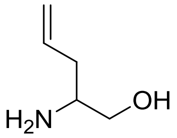

To a solution of MeOH (40 mL) was added acetyl chloride (4.0 g, 52 mmol); to this, 2-amino-4-pentenoic acid (2.00 g, 17.4 mmol) in MeOH (60 mL) was added. The reaction was heated under reflux for 4 h and concentrated in vacuo to give the crude methyl ester hydrochloride. THF (100 mL) was added, the solution was cooled to 0 °C, and $LiAlH_4$ (1.97 g, 52 mmol) was added portionwise (ca. 0.5 g). After 16 h, aqueous sat. NH_4Cl was added until effervescence ceased, the resulting solution was concentrated in vacuo onto silica gel. Column chromatography eluting with CH_2Cl_2–MeOH (85:15) gave the amino alcohol **S4** (1.62 g, 16 mmol, 92%) as a pale yellow oil. R_f 0.1 (90:10 CH_2Cl_2–MeOH); δ_H (300 MHz; $CDCl_3$) 5.58 (1H, ddt, *J* 17.1, 10.2 and 7.2, 4-H), 4.96-4.82 (2H, m, 5-H_{AB}), 3.39 (1H, dd, *J* 10.9, 3.9, 1-H_A), 3.16 (dd, *J* 10.9, 7.4, 1-H_B), 2.74 (1H, dq, *J* 9.7, 7.5 Hz, 2-H), 2.04 (1H, dt, *J* 12.5, 6.1, 3-H_A), 1.96-1.80 (1H, m, 2-H_B); δ_C (75 MHz; $CDCl_3$) 134.4 (4-C), 177.8 (5-C), 65.1 (1-C), 52.1 (2-C), 37.7 (3-C); ν_{max}/cm^{-1} (film) 3543, 3352, 2939, 2308, 1960, 1846, 1660, 1643, 1594, 1539, 1428, 1361.

(*R*)-2-Aminopent-4-enol ((*R*)-S4)

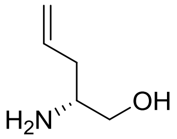

To a solution of MeOH (5 mL) was added acetyl chloride (3.4 g, 43 mmol); to this, (*R*)-2-amino-4-pentenoic acid (1.00 g, 8.7 mmol) in MeOH (10 mL) was added. The reaction was heated under reflux for 4 h and concentrated in vacuo to give the crude methyl ester hydrochloride. THF (100 mL) was added, the solution was cooled to 0 °C, and $LiAlH_4$ (0.66 g, 17.4 mmol) was added portionwise (ca. 0.2 g). After 16 h, aqueous sat. NH_4Cl was added until effervescence ceased, and the resulting solution was concentrated in vacuo onto silica gel. Column chromatography elution with CH_2Cl_2–EtOH–NH_4OH (86:13.5:1.5) gave the amino alcohol **(*R*)-S4** (700 mg, 6.93 mmol, 80%) as a pale yellow oil; $[\alpha]^D_{23.7}$: −25.4 (*c* 0.8, MeOH).

***N*-(1-Hydroxypent-4-en-2-yl)benzamide (S5)**

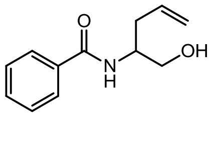

Benzoyl chloride (124 mg, 0.9 mmol) was added to a solution of the amino alcohol **S4** (100 mg, 0.99 mmol) and Et_3N (156 mg, 1.5 mmol) in CH_2Cl_2 (10 mL). After 24 h the reaction was concentrated in vacuo onto silica, column chromatography eluting with petrol–EtOAc (10:90 → 20:80) gave the amide **S5** (104 mg, 0.51 mmol, 51%) as an off-white solid; R_f 0.29 (90:10 petrol–EtOAc); δ_H (500 MHz; $CDCl_3$) 7.76 (2H, dd, *J* 8.3 and 1.4, Ar 2 and 6-H), 7.51 (1H, tt, *J* 7.5 and 1.4, Ar 4-H), 7.43 (2H, dd, *J* 8.3 and 7.5, Ar 3 and 5-H), 6.38 (1H, br s, NH), 5.86 (1H, ddt, *J* 17.2, 10.1 and 7.1, 4-H), 5.20 (1H, ddd, *J* 17.2, 1.7 and 1.6, 5-H_A), 5.17 (1H, ddd, *J* 10.1, 1.3 and 1.7, 5-H_B), 4.25-4.19 (1H, m, 2-H), 3.81 (1H, dd, *J* 11.1 and 3.7, 1-H_A), 3.75 (1H, dd, *J* 11.1 and 5.4, 1-H_B), 2.88 (1H, br s, OH), 2.51-2.38 (2H, m, 3-H_{AB}); δ_C (75 MHz; $CDCl_3$) 168.2 (C=O), 134.3 (5-C), 134.2 (Ar 1-C), 131.7 (Ar 4-C), 128.6 (Ar 2 and 6-C), 126.9 (Ar 3 and 5-C), 118.5 (4-C), 65.4 (1-C), 51.6 (2-C), 35.8 (3-C); ν_{max}/cm^{-1} (film) 3302, 2952, 1955, 1894, 1637, 1603, 1578, 1536, 1490, 1442; *m/z* (ES+) 228.1 (100%, $[M+Na]^+$); found 228.1002, $C_{12}H_{15}NO_2$ requires *MNa*, 228.0995. See Section S7 for details of the chiral HPLC analysis undertaken.

(*R*)-*N*-(1-Hydroxypent-4-en-2-yl)benzamide ((*R*)-S5)

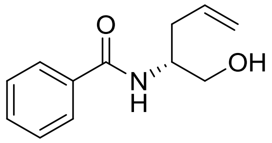

Benzoyl chloride (124 mg, 0.9 mmol) was added to a solution of **(*R*)-S4** (101 mg, 0.1 mmol) and Et_3N (150 mg, 1.5 mmol) in CH_2Cl_2 (10 mL). After 24 h the reaction was concentrated in vacuo onto silica, and column chromatography eluting with petrol–EtOAc (10:90 → 20:80) gave the amide **(*R*)-S5** (120 mg, 0.58 mmol, 58%) as an off-white solid, $[\alpha]^D_{23.7}$: 11.3 (*c* 0.5, MeOH), spectroscopically identical to that obtained previously.

***N*-(But-3-enyl)-1,1,1-trifluoromethanesulfonamide (12a)**

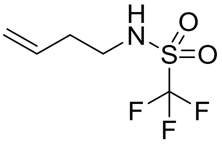

Triflic anhydride (2.56 mL, 15.0 mmol) was added dropwise to a stirred solution of but-3-enyl amine hydrochloride (1.50 g, 14.0 mmol) and triethylamine (4.85 mL, 35.0 mmol) in dichloromethane (20 mL) at 0 °C. After it was stirred at 0 °C for 1 h, the reaction mixture was warmed to room temperature and stirred for 5 h. After the reaction was quenched by the addition of water (20 mL), the pH was checked and adjusted to neutrality, the reaction mixture was extracted with dichloromethane (3 × 40 mL), and the combined organic fractions were washed with a saturated aqueous solution of sodium bicarbonate (30 mL) and brine (30 mL). After removal of solvent under reduced pressure, the crude product was purified by column chromatography, eluting with 50:50→0:100 petrol–EtOAc, to afford the sulfonamide **12a** (1.65 g, 58%) as pale yellow oil, R_F: 0.8 (50:50 EtOAc:petrol); δ_H (500 MHz; $CDCl_3$) 5.72 (1H, ddt, *J* 19.1, 9.8 and 6.9, butenyl 3-H), 5.26 (1H, br. s, N-H), 5.17 (1H, s, butenyl 4-H_a), 5.15 (1H, d, *J* 6.1, butenyl 4-H_b), 3.34 (2H, s, butenyl 1-H), 3.34 (2H, app q, *J* 6.8, butenyl 2-H); δ_C (75 MHz; $CDCl_3$) 133.3 (butenyl 3-H), 119.6 (butenyl

4-H), 119 (butenyl 4-H), 43.5 (butenyl 1-H), 34.3 (butenyl 2-H); ν_{max}/cm^{-1} (film) 3319, 2922, 1715, 1644, 1434, 1374; *m/z* (ES) 226.1 (100%, $M+Na^+$); HRMS Found: 202.0144, $C_5H_7F_3NO_2S$ requires *M-H* 202.015.

***N*-(2-Nitrophenylsulfonyl)pent-4-enamide (13)**

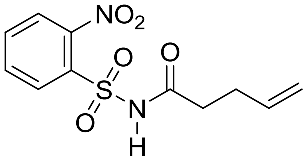

2-Nitrobenzenesulfonamide (1.0 g, 5.0 mmol) was combined with isopropylacetate, triethylamine (1.06 g, 10.3 mmol) and DMAP (6.0 mg, 0.05 mmol) under nitrogen forming a light yellow solution. The mixture was heated to 55 °C and to this was added a toluene (10 mL) solution of pent-4-enoyl chloride (0.652 g, 5.5 mmol) over 1h via a syringe pump with the temperature maintained at 50-60 °C. During the addition the amine salt precipitated as white slurry. Water was added and the mixture was washed with 0.7 M HCl. The layers were separated, the aqueous phase was discarded, and the organic phase was washed with water and brine, dried ($MgSO_4$), concentrated in vacuo, and purified by flash column chromatography (gradient elution: 10:90 → 30:70, ethyl acetate–petrol) to afford the sulfonamide **13** (0.97 g, 69%) as colourless amorphous solid; R_f 0.29 (30:70, EtOAc:petrol); ^1H ($CDCl_3$, 500 MHz) δ 8.47 (1H, s, *N-H*), 8.41 (1H, m, nosyl), 7.78-7.35 (3H, m, nosyl), 5.75 (1H, m, 4-H), 5.03 (1H, dd, *J* 1.7, 17.1, 5-H_A), 4.99 (1H, dd, *J* 1.7, 10.3, 5-H_B), 2.46 (2H, t, *J* 7.3, 2-CH_2), 2.38 (2H, q, J 7.3, 3-CH_2); ^{13}C ($CDCl_3$, 75 MHz) δ 172.8 (C=O), 135.7 (nosyl), 135.1 (C-4), 134.0 (nosyl), 132.8 (nosyl), 132.0 (nosyl), 131.8 (nosyl), 125.0 (nosyl), 116.7 (C-5), 35.8 (C-3), 25.2 (C-2); ν_{max}/cm^{-1} (film) 3263, 3079, 2940, 2912, 1850, 1732, 1640; m/z (ES) [M+H] 285.1 (37%, M+H), 307 (63%, M+Na); HRMS Found: 307.0362, $C_{11}H_{12}N_2Na_1O_5S_1$ requires 307.0359.

4 Synthesis of Metathesis Substrates

(*S*)-4-(1,1,1-Trifluoro-*N*-(1-(4-(((3,3,4,4,5,5,6,6,7,7,8,8,9,9,10,10,10-heptadecafluorodecyl)diisopropyl-silyloxy)methyl)phenyl)allyl) methylsulfonamido)but-2-ynyl acetate (S6)

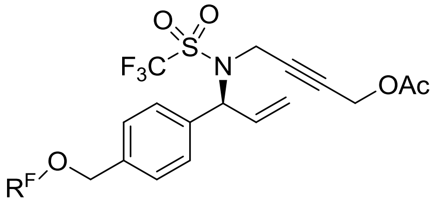

By general method **A1**, the fluorous-tagged sulfonamide **6a** (1.8 g, 2.1 mmol) and alcohol **8** (1.07 g, 8.4 mmol) gave a crude product, which was purified by F-SPE to afford the acetate **S6** (1.2 g, 70%) as yellow syrup, R_f 0.60 (1:4, EtOAc:petrol); δ_H (500 MHz; $CDCl_3$) 7.34 (4H, q, *J* 12.1, 8.9, Ar), 6.36 (1H, m, alkene-H), 5.68 (1H, d, *J* 7.3, allyl-H), 5.48 (1H, d, *J* 10, alkene-CH_2-H_A), 5.40 (1H, d, *J* 17.1, alkene-CH_2-H_B), 4.81 (2H, s, benzyl-CH_2), 4.54 (2H, t, *J* 1.9, alkyne-CH_2), 4.17 (1H, d, *J* 15.6, alkyne-CH_2-H_A), 3.87 (1H, d, *J* 18.8, alkyne-CH_2-H_B), 2.12 (2H, m, tag-CH_2), 2.07 (3H, s, acetate), 1.12 (2H, m, tag), 1.08 (6H, d, *J* 2.6, tag), 1.06 (6H, d, *J* 2.6, tag), 0.91 (2H, m, tag); δ_C (75 MHz; $CDCl_3$) 170.4 (carbonyl), 141.9 (Ar), 135.1 (Ar), 128.7 (alkene), 126.6 (Ar), 120.6 (alkene), 80.9 (alkyne), 70.9 (alkyne), 65.6 (allyl), 65.0 (benzyl), 52.1 (alkyne), 35.5 (alkyne), 25.6 (tag), 20.9 (acetate), 17.8 (tag), 17.7 (tag), 12.7 (tag), 0.1 (tag); m/z (ES) [M+NH_4] 983.2 (100%, M+NH_4); HRMS Found: 988.1567, $C_{33}H_{35}F_{20}N_1Na_1O_5S_1Si_1$ requires 988.1578.

(*S*)-1,1,1-Trifluoro-*N*-(1-(4-(((3,3,4,4,5,5,6,6,7,7,8,8,9,9,10,10,10-heptadecafluorodecyl)diisopropyl-silyloxy)methyl)phenyl)allyl)-*N*-(4-hydroxybut-2-ynyl)methanesulfonamide (25)

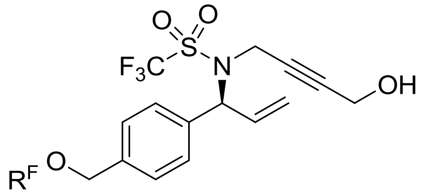

By the general method for deacetylation, the acetate **S6** (1.10 g, 1.13 mmol) gave the alcohol **25** (0.967 g, 92%), which was used without further purification, R_f 0.57 (1:4, EtOAc:petrol); δ_H (500 MHz; $CDCl_3$) 7.40 (2H, d, *J* 8.1, Ar), 7.36 (2H, d, *J* 8.1, Ar), 6.31 (1H, ddd, *J* 17.1, 10.7, 6.8, alkene-*CH*), 5.71 (1H, d, *J* 6.8, allyl-*CH*), 5.50 (1H, d, *J* 10.7, alkene-CH_2-H_A), 5.39 (1H, d, *J* 17.1, alkene-CH_2-H_B), 4.79 (2H, s, benzyl), 4.11 (1H, d, *J* 17.1, alkyne-CH_2-H_A), 4.05 (2H, s, alkyne-CH_2), 3.99 (1H, d, *J* 17.1, alkyne-CH_2-H_B), 2.11 (2H, m, tag), 1.16-1.03 (14H, m, tag), 0.92 (2H, m, tag); δ_C (75 MHz; $CDCl_3$) 141.8 (Ar), 129.0 (Ar), 126.9 (alkene), 122.1 (Ar), 120.3 (alkene), 83.9 (alkyne), 79.8 (alkyne), 65.2 (allyl), 65.1 (benzyl), 50.1 (alkyne), 35.6 (alkyne), 25.7 (tag), 17.9 (tag), 17.8 (tag), 12.7 (tag), 0.5 (tag); m/z (ES) [M+Na] 946.3 (100%, M+Na); HRMS Found: 946.1476, $C_{31}H_{33}F_{20}N_1Na_1O_4S_1Si_1$requires 946.1472.

(*S*)-6-(1,1,1-Trifluoro-*N*-(1-(4-(((3,3,4,4,5,5,6,6,7,7,8,8,9,9,10,10,10-heptadecafluorodecyl) diisopropylsilyloxy)methyl)phenyl)allyl)methylsulfonamido)hexa-2,4-diynyl acetate (S7)

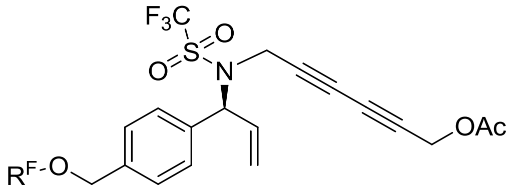

By general method **A2**, the fluorous-tagged sulfonamide **6a** (1.7 g, 1.99 mmol) and the alcohol **9** (1.2 g, 7.95 mmol) gave a crude product, which was purified by F-SPE to afford the acetate **S7** (1.9 g, 97%), R_f 0.60 (20:80, EtOAc:petrol); δ_H (500 MHz; $CDCl_3$) 7.36 (4H, q, *J* 9.4, Ar), 6.33 (1H, ddd, *J* 17.1, 10.2, 7.2, alkene-CH), 5.68 (1H, d, *J* 7.2, allyl-H), 5.51 (1H, d, *J* 10.2, alkene-CH_2-H_A), 5.40 (1H, d, *J* 17.1, alkene-CH_2-H_B), 4.81 (2H, s, benzyl), 4.71 (2H, s, diyne-CH_2), 4.21 (1H, d, *J* 18.3, diyne-CH_2-H_A), 3.88 (1H, d, *J* 18.3, diyne-CH_2-H_B), 2.10 (5H, m, acetate, tag), 1.16-1.02 (14H, m, tag), 0.88 (2H, m, tag); δ_C (75 MHz; $CDCl_3$) 170.3 (carbonyl), 142.1 (Ar), 134.9 (Ar), 128.7 (alkene), 126.8 (Ar), 120.8 (alkene), 73.9 (diyne), 73.4 (diyne), 70.5 (diyne), 69.4 (diyne), 65.6 (alkene), 64.9 (benzyl), 52.5 (diyne), 35.8 (diyne), 25.7 (tag), 20.9 (acetate), 17.9 (tag), 17.8 (tag), 12.7 (tag), 0.1 (tag); ν_{max}/cm^{-1} (film) 2947, 2869, 1755, 1513, 1464; m/z (ES) [M+Na] 1012.2 (100%, M+Na); HRMS Found: 1012.159, $C_{35}H_{35}F_{20}N_1Na_1O_5S_1Si_1$ requires 1012.1578.

(*S*)-1,1,1-Trifluoro-*N*-(1-(4-(((3,3,4,4,5,5,6,6,7,7,8,8,9,9,10,10,10-heptadecafluorodecyl)diisopropyl-silyloxy)methyl)phenyl)allyl)-*N*-(6-hydroxyhexa-2,4-diynyl)methanesulfonamide (26)

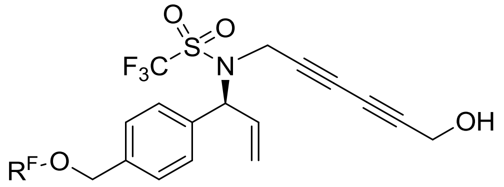

By the general method for deacetylation, the acetate **S7** (1.9 g, 1.92 mmol) gave the alcohol **26** (1.59 g, 87%), which was used without further purification. R_f 0.40 (20:80, EtOAc:petrol); δ_H (500 MHz; $CDCl_3$) 7.36 (4H, s, Ar), 6.33 (1H, ddd, *J* 17.2, 10.2, 7.2, alkene-*CH*), 5.69 (1H, d, *J* 7.2, allyl-H), 5.51 (1H, d, *J* 10.2, alkene-CH_2-H_A), 5.40 (1H, d, *J* 17.2, alkene-CH_2-H_B), 4.82 (2H, s, benzyl), 4.32 (2H, d, *J* 6.4, diyne-CH_2), 4.21 (1H, d, *J* 17.2, diyne-CH_2-H_A), 3.90 (1H, d, *J* 17.2, diyne-CH_2-H_B), 2.10 (2H, m, tag-CH_2), 1.67 (1H, t, *J* 6.4, *OH*), 1.12 (2H, m, tag), 1.08, 1.06 (12H, d, *J* 2.5, tag), 0.91 (2H, m, tag); δ_C (75 MHz; $CDCl_3$) 142.1 (Ar), 133 (Ar), 128.6 (alkene), 126.7 (Ar), 117.8 (alkene), 73.6 (diyne), 69.8 (diyne), 69.5 (diyne), 65.6 (allyl), 65.0 (benzyl), 51.7 (diyne), 35.6 (diyne), 25.7 (tag), 17.9 (tag), 17.8 (tag), 12.7 (tag), 0.1 (tag); ν_{max}/cm^{-1} (film) 3575, 3399, 2948, 2870, 1514, 1463; m/z (ES) [M+Na] 970.1 (100%, M+Na); HRMS Found: 970.1492, $C_{33}H_{33}F_{20}N_1Na_1O_4S_1Si_1$ requires 970.1472.

(1*R*,4*R*)-4-(1,1,1-Trifluoro-*N*-((*S*)-1-(4-(((3,3,4,4,5,5,6,6,7,7,8,8,9,9,10,10,10-heptadecafluorodecyl)diisopropylsilyloxy)methyl)phenyl)allyl)methylsulfonamido)cyclopent-2-enyl acetate (S8)

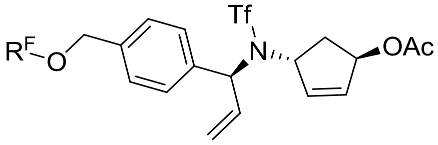

By general method **A3**, the fluorous-tagged sulfonamide **6a** (0.5 g, 0.58 mmol), and the alcohol **10** (0.330 g, 2.32 mmol) gave a crude product, which was purified by F-SPE to afford the acetate **S8** (0.481 mg**,** 85%), R_f 0.38 (10:90, EtOAc:petrol); δ_H (500 MHz; $CDCl_3$) 7.40 (2H, d, *J* 8.3, Ar), 7.36 (2H, d, *J* 8.3, Ar), 6.15 (1H, ddd, *J* 16.6, 10.4, 6.2, 3-H), 6.01 (1H, d, *J* 5.2, 6-H), 5.98 (1H, d, *J* 5.2, 7-H), 5.69 (1H, d, *J* 6.2, 2-H), 5.52 (1H, d, *J* 10.4, 4-H_A), 5.38 (1H, d, *J* 16.6, 4-H_B), 4.82 (2H, s, benzyl), 2.22 (1H, ddd, *J* 12.5, 7.3, 4.7), 2.13 (3H, m, tag), 1.95 (3H, s, acetate), 1.12 (2H, m,), 1.02 (14H, m, tag), 0.91 (2H, m, tag); δ_C (75 MHz; $CDCl_3$) 170.9 (carbonyl), 141.5, 134.1 (C-7), 134.3, 121.4 (C-4), 78.5 (C-8), 76.4 (C-5), 64.9 (C-2), 64.8 (benzyl), 35.8 (C-9), 25.5 (tag), 21.0 (acetate), 17.6 (tag), 17.5 (tag), 12.5 (tag), 1.46 (tag); ν_{max}/cm^{-1} (film) 2948, 2870, 1741, 1513, 1464, 1397; m/z (ES) [M+NH_4] 997.2 (100%, M+NH_4); HRMS Found: 997.2179, $C_{34}H_{41}F_{20}N_2O_5S_1Si_1$ requires 997.2181.

1,1,1-Trifluoro-*N*-((*S*)-1-(4-(((3,3,4,4,5,5,6,6,7,7,8,8,9,9,10,10,10-heptadecafluorodecyl)diisopropyl-silyloxy)methyl)phenyl)allyl)-*N*-((1*R*,4*R*)-4-hydroxycyclopent-2-enyl)methanesulfonamide (27)

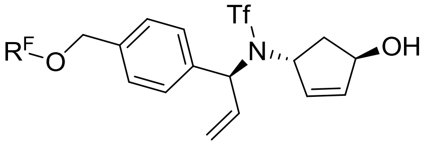

By the general method for deacetylation, the acetate **S8** (1.0 g, 1.02 mmol) gave the *alcohol* **27** (0.910 g, 87%), which was used without further purification, R_f 0.20 (20:80, EtOAc:petrol); δ_H (500 MHz; $CDCl_3$) 7.40 (2H, d, *J* 8.3, Ar), 7.34 (2H, d, *J* 8.3, Ar), 6.13 (1H, ddd, *J* 16.6, 10.3, 6.3, 3-H), 5.99 (1H, d, *J* 4.5, 6-H), 5.88 (1H, d, *J* 4.5, 7-H), 5.50 (1H, d, *J* 10.2, 4-H_A), 5.37 (1H, d, *J* 16.6, 4-H_B), 4.93 (1H, s, 2-H), 4.81 (2H, s, benzyl), 2.11 (3H, m, 5-H & tag), 1.42 (2H, m, 11-H), 2.08 (14H, m, tag), 0.92 (2H, m, tag); δ_C (100 MHz; $CDCl_3$) 141.6 (Ar), 138.1 (C-3), 136.7 (Ar), 133.9 (C-6), 128.5 (C-7), 120 (C-4), 126.5 (Ar), 76.2 (C-8), 65.4 (C-5), 65.1 (C-2), 65.0 (benzyl), 41.5 (C-9), 25.7 (tag), 17.9 (tag), 17.8 (tag), 12.7 (tag), 0.2 (tag); ν_{max}/cm^{-1} (film) 3364, 2948, 2870, 1396, 1381; m/z (ES) [M+NH_4] 955.2 (100%, M+NH_4); HRMS Found: 955.2104, $C_{32}H_{39}F_{20}N_2O_4S_1Si_1$ requires 955.2075.

((1*R*,4*S*)-4-((*N*-((*S*)-1-(4-(((3,3,4,4,5,5,6,6,7,7,8,8,9,9,10,10,10-Heptadecafluorodecyl)diisopropyl-silyloxy)methyl)phenyl)allyl)-2-nitrophenylsulfonamido)methyl)cyclopent-2-enyl)methyl acetate (S9)

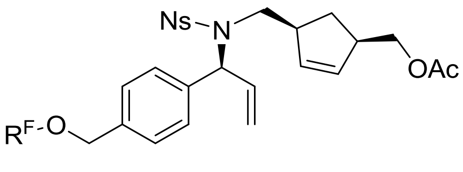

By general method **A3**, the fluorous-tagged sulfonamide **6b** (0.5 g, 0.55 mmol) and the alcohol **11** (0.374 g, 2.20 mmol) gave a crude product, which was purified by F-SPE to afford acetate **S9** (0.481 mg**,** 85%; ca. 75:25 mixture of diastereomers), R_F: 0.58 (30:70, EtOAc:petrol); δ_H (500 MHz; $CDCl_3$) 7.98 (1H, d, *J* 7.8,

nosyl), 7.68 (1H, d, *J* 7.8, nosyl), 7.63 (2H, q, *J* 7.8, nosyl), 7.35 (2H, d, *J* 8.3, Ar), 7.29 (2H, d, *J* 8.3, Ar), 6.14 (1H, ddd, *J* 17.6, 10.3, 7.3, 3-H), 5.69 (1H, d, *J* 7.3, 2-H), 5.53 (1H, dt, *J* 4.2, 2.1, 7-H), 5.37 (1H, dt, *J* 4.2, 2.1, 8-H), 5.29 (1H, d, *J* 10.3, 4-H_A), 5.16 (1H, d, *J* 17.6, 4-H_B), 4.81 (2H, s, benzyl), 3.99 (1H, dd, *J* 10.9, 6.7, 10-H_A), 3.93 (1H, dd, *J* 10.9, 6.7, 10-H_B), 3.36 (2H, d, *J* 7.3, 5-H), 2.86 (1H, m, 9-H), 2.68 (1H, tt, *J* 6.2, 1.6, 6-H), 2.15 (2H, m, tag), 2.07 (3H, s, acetate), 1.98 (1H, m, 11-H_A), 1.26 (1H, quin, *J* 6.7, 11-H_B), 1.10 (14H, m, tag), 0,94 (2H, m, tag); δ_C (75 MHz; $CDCl_3$) 171.4 (carbonyl), 160.9 (nosyl), 148.3 (nosyl), 141.2 (Ar), 141.0 (Ar), 137.0 (Ar), 136.9 (Ar), 134.5, 134.2, 134.1, 134.0, 133.9, 133.7, 133.5 (C-7), 132.3, 132.0, 131.6 (C-8), 131.3, 131.2, 128.7, 128.6, 126.3, 126.2 (Ar), 126.1 (Ar), 124.2 (nosyl), 120.0, 119.6 (C-4), 119.2, 67.9 (C-10), 64.9 (benzyl), 63.3 (C-2), 63.2, 51.8 (C-5), 51.7 (C-5), 46.0 (C-9), 45.0 (C-6), 31.7 (C-11), 25.5 (tag), 21.1 (acetate), 17.7 (tag), 17.6 (tag), 12.5 (tag), 0.0 (tag); ν_{max}/cm^{-1} (film) 2947, 2869, 1739, 1546, 1372; m/z (ES) [$M+NH_4$] 1078.3 (100%, $M+NH_4$); HRMS Found: 1083.2321, $C_{41}H_{45}F_{17}N_2Na_1O_7S_1Si_1$ requires 1083.2337.

***N*-((*S*)-1-(4-(((3,3,4,4,5,5,6,6,7,7,8,8,9,9,10,10,10-Heptadecafluorodecyl)diisopropylsilyloxy)methyl)-phenyl)allyl)-*N*-(((1*S*,4*R*)-4-(hydroxymethyl)cyclopent-2-enyl)methyl)-2-nitrobenzenesulfonamide (28)**

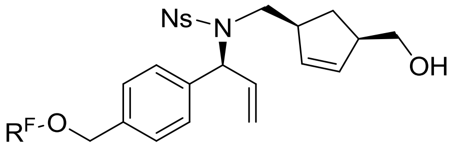

By the general method for deacetylation, the acetate **S9** (1.6 g, 1.50 mmol) gave the alcohol **28** (1.3 g, 85%; ca. 75:25 mixture of diastereomers), which was used without further purification, R_f 0.18 (30:70, EtOAc:petrol); δ_H (500 MHz; $CDCl_3$) 7.91 (1H, dd, *J* 7.0, 1.7, nosyl), 7.63 (1H, dd, *J* 7.0, 1.7, nosyl), 7.59 (2H, m, nosyl), 7.33 (2H, d, *J* 8.3, Ar), 7.26 (2H, d, *J* 8.3, Ar), 6.12 (1H, ddd, *J* 17.6, 10.6, 7.0, 3-H), 5.65 (1H, d, *J* 7, 2-H), 5.52 (1H, dt, *J* 7.7, 2.1, 7-H), 5.40 (1H, dt, *J* 7.7, 2.1, 8-H), 5.25 (1H, d, *J* 10.6, 4-H_A), 5.21 (1H, d, *J* 17.6, 4-H_B), 4.76 (2H, s, benzyl), 3.49 (2H, dd, *J* 5.5, 4.2, 10-H), 3.32 (2H, t, *J* 7.0, 5-H), 2.74 (1H, m, 6-H), 2.60 (1H, m, H-9), 2.11 (2H, m, tag), 1.87 (1H, dt, *J* 22.0, 9.0, 11-H_A), 1.33 (1H, dt, *J* 19.0, 5.3, 11-H_B), 1.09 (14H, m, tag), 0.90 (2H, m, tag); δ_C (75 MHz; $CDCl_3$) 148.3 (nosyl), 141.0 (nosyl), 137.2 (C-3), 133.9, 133.8 (nosyl), 133.5 (nosyl), 132.9 (nosyl), 131.5, 130.9, 128.5 (Ar), 126.2 (Ar), 124.1 (nosyl), 120.1 (C-4), 66.1 (C-10), 64.9 (benzyl), 63.2 (C-2), 51.6 (C-5), 48.4 (C-6), 45.7 (C-9), 30.5 (C-11), 25.6 (tag), 17.7 (tag), 17.6 (tag), 12.5 (tag), 0.1 (tag); ν_{max}/cm^{-1} (film) 3359, 2947, 2869, 1668, 1547; m/z (ES) [$M+NH_4$] 1036.3 (100%, $M+NH_4$); HRMS Found: 1041.2276, $C_{39}H_{43}F_{17}N_2Na_1O_6S_1Si_1$ requires 1041.2232.

(*R*)-4-(*N*-(1-((3,3,4,4,5,5,6,6,7,7,8,8,9,9,10,10,10-Heptadecafluorodecyl)diisopropylsilyloxy)pent-4-en-2-yl)-2-nitrophenylsulfonamido)but-2-ynyl acetate (S10)

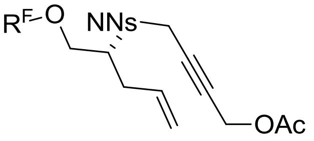

By general method **A3**, the fluorous-tagged sulfonamide **7** (0.2 g, 0.23 mmol) and the alcohol **8** (0.95 g, 0.95 mmol) gave a crude product, which was purified by F-SPE to afford the acetate **S10** (0.198 mg**,** 92%), R_f 0.56 (30:70, EtOAc:petrol); δ_H (500 MHz; $CDCl_3$) 8.15 (1H, m, nosyl), 7.67 (2H, m, nosyl), 7.62 (1H, m, nosyl), 5.63 (1H, m, 4-H), 5.05 (1H, d, *J* 17.3, 5-H_A), 4.95 (1H, d, *J* 10.6, 5-H_B), 4.49 (2H, s, 1'-CH_2 alkyne), 4.32 (2H, q, *J* 18.8, 4'-CH_2 alkyne), 4.00 (1H, m, 2-H), 3.87 (2H, d, *J* 5.1, 1-CH_2), 2.52 (1H, quin, *J* 7.3, 3-H_A), 2.46 (1H, quin, *J* 7.3, 3-H_B), 2.09 (2H, m, tag), 2.04 (3H, s, CH_3-acetate), 1.02 (14H, s, tag), 0.82 (2H, m, tag); δ_C (75 MHz; $CDCl_3$) 170.3 (carbonyl), 148.4 (nosyl), 134.3 (C-4), 133.9 (nosyl), 131.2 (nosyl), 124.4 (nosyl), 118.4 (C-5), 82.8 (C-2' alkyne), 78.9 (C-3' alkyne), 65.0 (C-1), 59.7 (C-2), 52.3 (C-1'), 34.4 (C-4'), 34.3 (C-3), 25.6 (tag), 20.9 (methyl acetate), 17.7 (tag), 17.6 (tag), 12.5 (tag), 0.1 (tag); ν_{max}/cm^{-1} (film) 3697, 2946, 2869, 1751, 1545, 1438;); m/z (ES) [M+NH_4] 974.2 (100%, M+NH_4); HRMS Found: 974.2159, $C_{33}H_{37}F_{17}N_2Na_1O_7S_1Si_1$ requires 974.2163.

(*R*)-*N*-(1-((3,3,4,4,5,5,6,6,7,7,8,8,9,9,10,10,10-Heptadecafluorodecyl)diisopropylsilyloxy)pent-4-en-2-yl)-*N*-(4-hydroxybut-2-ynyl)-2-nitrobenzenesulfonamide (29)

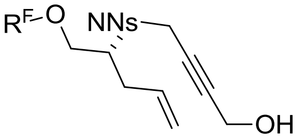

By the general procedure for deacetylation, the acetate **S10** (1.9 g, 1.98 mmol) was dissolved in ammonia-saturated methanol solution (82 mL, 0.025 M) and was stirred at room temperature overnight. The solvent was removed under reduced pressure to give the crude product, which was purified by flash chromatography (gradient elution: 10:90 → 40:60, ethyl acetate–petrol) to afford the *alcohol* **15** (1.67 g, 94%) R_f 0.67 (40:60, EtOAc:petrol); δ_H (500 MHz; $CDCl_3$) 8.16 (1H, dd, *J* 7.2, 1.9, nosyl), 7.66 (3H, m, nosyl), 5.65 (1H, m, 4-H), 5.06 (1H, dd, *J* 16.8, 1.5, 5-H_A), 4.96 (1H, d, *J* 10.4, 5-H_B), 4.30 (2H, qt, *J* 18.5, 1.7, 4'-H_B alkyne), 4.09 (2H, d, *J* 5.3, 1'-CH_2 alkyne), 4.01 (1H, m, 2-H), 3.88 (2H, d, *J* 5.1, 1-H), 2.51 (2H, m, 3-CH_2), 2.09 (2H, m, tag), 1.51 (1H, t, *J* 5.3, OH), 1.03 (14H, m, tag), 0.83 (2H, m, tag); δ_C (75 MHz; $CDCl_3$) 148.3 (nosyl), 134.3 (C-4), 134.3 (nosyl), 133.8 (nosyl), 132.0 (nosyl), 131.8 (nosyl), 124.0 (nosyl), 118.4 (C-5), 82.9 (C-2' alkyne), 81.9 (C-3' alkyne), 65.0 (C-1), 59.8 (C-2), 51.2 (C-1' alkyne), 34.4 (C-4' alkyne), 34.3 (C-3), 25.6 (tag), 17.7 (tag), 17.6 (tag), 12.5 (tag), 0.1 (tag); ν_{max}/cm^{-1} (film) 3564, 3081, 2947, 2870, 1727, 1548; m/z (ES) [M+NH_4] 932.2 (100%, M+NH_4); HRMS Found: 937.1608, $C_{31}H_{35}F_{17}N_2Na_1O_6S_1Si_1$ requires 937.1606.

(*R*)-6-(*N*-(1-((3,3,4,4,5,5,6,6,7,7,8,8,9,9,10,10,10-Heptadecafluorodecyl)diisopropylsilyloxy)pent-4-en-2-yl)-2-nitrophenylsulfonamido)hexa-2,4-diynyl acetate (S11)

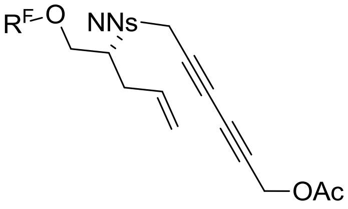

By general method **A3**, the fluorous-tagged sulfonamide **7** (0.2 g, 0.23 mmol) and the alcohol **9** (0.145 g, 0.95 mmol) gave a crude product, which was purified by flash chromatography (gradient elution: 10:90 → 33:67, ethyl acetate–petrol) to afford the acetate **S11** (0.171 g, 74%) R_f 0.65 (30:70, EtOAc:petrol); δ_H (500 MHz; $CDCl_3$) 8.12 (1H, m, nosyl), 7.68 (2H, m, nosyl), 7.63 (1H, m, nosyl), 5.63 (1H, m, 4-H), 5.07 (1H, dd, *J* 17.1, 1.5, 5-H_A), 4.97 (1H, dd, *J* 10.2, 5-H_B), 4.67 (2H, s, 1'-CH_2 diyne), 4.42 (1H, d, *J* 19.4, 6'-H_A, diyne), 4.32 (1H, d, *J* 19.4, 6'-H_B diyne), 4.02 (1H, m, 2-H), 3.87 (2H, dd, *J* 4.9, 1.5, 1-CH_2), 2.50 (1H, m, 3-H_A), 2.44 (1H, m, 3-H_B), 2.08 (3H, s, CH_3 acetate), 2.07 (2H, m, tag), 1.02 (14H, m, tag), 0.83 (2H, m, tag); δ_C (75 MHz; $CDCl_3$) 170.3 (carbonyl), 148.3 (nosyl), 134.0 (nosyl), 133.9 (C-4), 131.9 (nosyl), 124.5 (nosyl), 118.5 (C-5), 75.9 (C-2' diyne), 73.0 (C-3' diyne), 70.5 (C-4' diyne), 68.8 (C-5' diyne), 65.0 (C-1), 59.5 (C-2), 52.4 (C-1' diyne), 34.8 (C-6' diyne), 34.2 (C-3), 25.6 (tag), 20.9 (C-Acetate), 17.8 (tag), 17.7 (tag), 12.5 (tag), 0.1 (tag); ν_{max}/cm^{-1} (film) 3444, 2974, 2869, 1751, 1643, 1544; m/z (ES) [M+NH_4] 998.2 (100%, M+NH_4); HRMS Found: 998.2168, $C_{35}H_{41}F_{17}N_3O_7S_1Si_1$ requires 998.2158.

(*R*)-*N*-(1-((3,3,4,4,5,5,6,6,7,7,8,8,9,9,10,10,10-Heptadecafluorodecyl)diisopropylsilyloxy)pent-4-en-2-yl)-*N*-(4-hydroxybut-2-ynyl)-2-nitrobenzenesulfonamide (30)

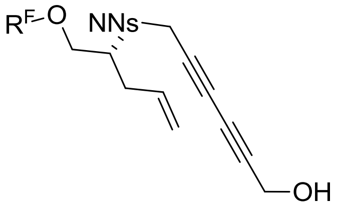

By the general method for deacetylation, the acetate **S11** (1.51 g, 1.53 mmol) gave the *alcohol* **30** (1.3 g, 97%), which was used without further purification, R_f 0.67 (40:60, EtOAc:petrol); δ_H (500 MHz; $CDCl_3$) 8.16 (1H, dd, *J* 7.2, 1.9, nosyl), 7.66 (3H, m, nosyl), 5.65 (1H, m, 4-H), 5.06 (1H, dd, *J* 16.8, 1.5, 5-H_A), 4.96 (1H, d, *J* 10.4, 5-H_B), 4.30 (2H, qt, *J* 18.5, 1.7, 4'-H_B alkyne), 4.09 (2H, d, *J* 5.3, 1'-CH_2 alkyne), 4.01 (1H, m, 2-H), 3.88 (2H, d, *J* 5.1, 1-H), 2.51 (2H, m, 3-CH_2), 2.09 (2H, m, tag), 1.51 (1H, t, *J* 5.3, OH), 1.03 (14H, m, tag), 0.83 (2H, m, tag); δ_C (75 MHz; $CDCl_3$) 148.3 (nosyl), 134.3 (C-4), 134.3 (nosyl), 133.8 (nosyl), 132.0 (nosyl), 131.8 (nosyl), 124.0 (nosyl), 118.4 (C-5), 82.9 (C-2' alkyne), 81.9 (C-3' alkyne), 65.0 (C-1), 59.8 (C-2), 51.2 (C-1' alkyne), 34.4 (C-4' alkyne), 34.3 (C-3), 25.6 (tag), 17.7 (tag), 17.6 (tag), 12.5 (tag), 0.1 (tag); ν_{max}/cm^{-1} (film) 3564, 3081, 2947, 2870, 1727, 1548; m/z (ES) [M+NH_4] 932.2 (100%, M+NH_4); HRMS Found: 937.1572, $C_{33}H_{35}F_{17}N_2Na_1O_6S_1Si_1$ requires 937.1608.

(1*R*,4*R*)-4-(*N*-((*R*)-1-((3,3,4,4,5,5,6,6,7,7,8,8,9,9,10,10,10-Heptadecafluorodecyl)diisopropylsilyloxy) pent-4-en-2-yl)-2-nitrophenylsulfonamido)cyclopent-2-enyl acetate (S12)

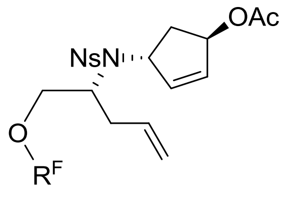

By general method **A3**, the fluorous-tagged sulfonamide **7** (0.200 g, 0.23 mmol) and the alcohol **10** (0.134 g, 0.95 mmol) gave a crude product, which was purified by F-SPE to afford the acetate **S12** (0.223 g, 97%) R_f 0.35 (20:80, EtOAc:petrol); δ_H (500 MHz; $CDCl_3$) 8.10 (1H, d, *J* 7.7, nosyl), 7.64 (3H, m, nosyl), 5.98 (2H, m, 2', 3'-H cyclopentene), 5.74 (1H, d, *J* 7.7, 1'-H cyclopentene), 5.69 (1H, m, 4-H), 5.10 (1H, m, 4'-H cyclopentene), 5.07 (1H, d, *J* 3.9, 5-H_A), 5.04 (1H, s, 5-H_B), 3.92 (1H, dd, *J* 10.9, 6.4, 1-H_A), 3.83 (1H, dd, *J* 10.9, 4.7, 1-H_B), 3.56 (1H, m, 2-H), 2.53 (2H, m, 3-H), 2.38 (1H, dd, *J* 14.5, 6.6, 5'-H_A), 2.19 (1H, ddd, *J* 14.9, 8.3, 2.4, 5'-H_B), 2.07 (2H, m, tag), 2.00 (3H, s, CH_3-acetate), 1.02 (14H, s, tag), 0.83 (2H, m, tag); δ_C (75 MHz; $CDCl_3$) 171.2 (carbonyl), 148.8 (nosyl), 137.6 (C-2' cyclopentene), 134.8 (nosyl), 135.1 (nosyl), 133.8 (C-4), 133.6 (nosyl), 133.5 (nosyl), 133.3 (C-3' cyclopentene), 124.4 (nosyl), 119.3 (C-5), 78.7 (C-1' cyclopentene), 64.8 (C-1), 63.8 (C-4' cyclopentene), 60.3 (C-2), 37.0 (C-5), 36.7 (C-3), 25.6 (tag), 21.3 (CH_3-acetate), 17.7 (tag), 17.6 (tag), 12.4 (tag), 0.1 (tag); ν_{max}/cm^{-1} (film) 3735, 2947, 2870, 1739, 1547, 1464; m/z (ES) [M+NH_4] 988.2 (100%, M+NH_4); HRMS Found: 988.2307, $C_{34}H_{43}F_{17}N_3O_7S_1Si_1$ requires 988.2314.

***N*-((*R*)-1-((3,3,4,4,5,5,6,6,7,7,8,8,9,9,10,10,10-Heptadecafluorodecyl)diisopropylsilyloxy)pent-4-en-2-yl)-*N*-((1*R*,4*R*)-4-hydroxycyclopent-2-enyl)-2-nitrobenzenesulfonamide (31)**

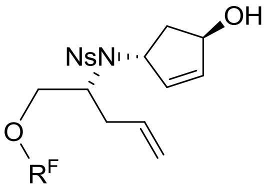

By the general method for deacetylation, the acetate **S12** (2.0 g, 2.09 mmol) gave the alcohol **31** (1.51 g, 80%) R_f 0.18 (30:70, EtOAc:petrol); δ_H (500 MHz; $CDCl_3$) 8.11 (1H, d, *J* 7.7, nosyl), 7.61 (3H, m, nosyl), 5.99 (1H, dt, *J* 5.6, 2.1, 2'-H cyclopentene), 5.84 (1H, dd, *J* 5.6, 1.2, 3'-H), 5.71 (1H, m, 4-H), 5.05 (4H, m, 5-CH_2, 1'-H, 4'-H cyclopentene), 3.91 (1H, dd, *J* 10.7, 5.1, 1-H_A), 3.81 (1H, dd, *J* 10.7, 5.1, 1-H_B), 3.50 (1H, bs, 2-H), 2.54 (2H, m, 3-CH_2), 2.34 (1H, quin, *J* 5.7, 5'-H_A cyclopentene), 2.08 (3H, m, 5-H_B, tag), 1.61 (1H, s, OH), 1.02 (14H, s, tag), 0.83 (2H, m, tag); δ_C (75 MHz; $CDCl_3$) 148.9 (nosyl), 137.6 (C-2' cyclopentene), 135.1 (nosyl), 135.0 (C-4), 134.8 (C-3' cyclopentene), 133.7 (nosyl), 131.7 (nosyl), 130.8 (nosyl), 124.4 (nosyl), 118.3 (C-5), 76.2 (C-1' cyclopentene), 64.7 (C-1), 64.2 (C-4' cyclopentene), 60.2 (C-2), 40.3 (C-5' cyclopentene), 36.8 (C-3), 25.6 (tag), 17.6 (tag), 12.4 (tag), 0.01 (tag); ν_{max}/cm^{-1} (film) 3735, 2948, 2870, 1547, 1463, 1440; m/z (ES) [M+NH_4] 946.2 (100%, M+NH_4); HRMS Found: 946.1797, $C_{32}H_{37}F_{17}N_2Na_1O_6S_1Si_1$ requires 946.1762.

(*S*)-*N*-(But-3-enyl)-2-nitro-*N*-(4-(1,1,1-trifluoro-*N*-(1-(4-(((3,3,4,4,5,5,6,6,7,7,8,8,9,9,10,10,10-hepta-decafluorodecyl)diisopropylsilyloxy)methyl)phenyl)allyl)methylsulfonamido)but-2-ynyl)benzene-sulfonamide (32)

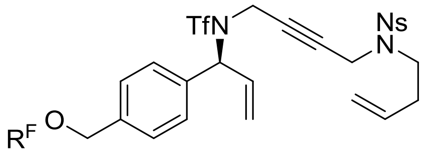

By general method **A4**, the fluorous-tagged alcohol **25** (0.8 g, 0.87 mmol) and the sulfonamide **12b** (0.891 g, 3.50 mmol) gave a crude product, which was purified by F-SPE to afford the metathesis substrate **32** (0.895 g, 89%), R_f 0.36 (20:80, EtOAc:petrol); δ_H (500 MHz; $CDCl_3$) 7.99 (1H, m, nosyl), 7.68 (2H, m, nosyl), 7.62 (1H, m, nosyl), 7.35 (2H, d, *J* 8.1, Ar), 7.32 (2H, d, *J* 8.1, Ar), 6.25 (1H, ddd, *J* 17.2, 10.2, 7.2, alkene-*CH*), 5.76 (2H, m, butenyl-*CH*, allyl-H), 5.45 (1H, d, *J* 10.2, alkene-CH_2-H_A), 5.34 (1H, d, *J* 17.1, alkene-CH_2-H_B), 5.06 (1H, dd, *J* 17.2, 1.7, butenyl-CH_2-H_A), 5.02 (1H, dd, *J* 10.5, 1.7, butenyl-CH_2-H), 4.81 (2H, s, benzyl), 4.20 (1H, m, alkyne-CH_2-1-H_A), 4.11 (1H, d, *J* 8.1, alkyne-CH_2-2-H_A), 4.05 (1H, d, *J* 21.0, alkyne-CH_2-1-H_B), 3.76 (1H, d, *J* 18.8, alkyne-CH_2-2-H_B), 3.38 (2H, t, *J* 7.4, butenyl-CH_2-2), 2.28 (2H, q, *J* 7.3, butenyl-CH_2-3), 2.11 (2H, m, tag), 1.27 (1H, m, tag), 1.15-1.02 (13H, m, tag), 0.92 (2H, m, tag); δ_C (75 MHz; $CDCl_3$) 148.5 (nosyl), 142.1 (Ar), 134.2 (alkene), 134.0 (Ar), 133.3 (nosyl), 132.2 (butenyl), 131.1 (nosyl), 128.6 (Ar), 126.7 (Ar), 124.6 (nosyl), 120.7 (alkene), 117.9 (butenyl), 80.2 (alkyne), 79.0 (alkyne), 65.6 (allyl), 64.9 (benzyl), 46.4 (butenyl), 36.8 (alkyne), 35.3 (alkyne), 32.2 (butenyl), 25.7 (tag), 17.9 (tag), 17.8 (tag), 12.7 (tag), 0.2 (tag); m/z (ES) [M+NH_4] 1179.2 (100%, M+NH_4); HRMS Found: 1184.1880, $C_{41}H_{43}F_{20}N_3Na_1O_7S_2Si_1$ requires 1184.1885.

(*S*)-*N*-(2-Nitrophenylsulfonyl)-*N*-(4-(1,1,1-trifluoro-*N*-(1-(4-(((3,3,4,4,5, 5,6,6,7,7,8,8,9,9,10,10,10-heptadecafluorodecyl)diisopropylsilyloxy)methyl)phenyl)allyl)methylsulfonamido)but-2-ynyl)pent-4-enamide (33)

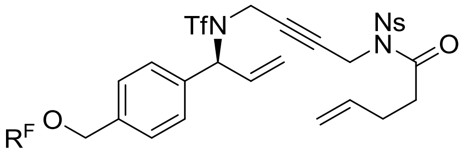

By general method **A4**, the fluorous-tagged alcohol **25** (0.190 g, 0.20 mmol) and the sulfonamide **13** (0.233 g, 0.82 mmol) gave a crude product, which was purified by F-SPE to afford the metathesis substrate **33** (0.220 g, 89%), R_f 0.48 (20:80, EtOAc:petrol); δ_H (500 MHz; $CDCl_3$) 8.42 (1H, m, nosyl), 7.79 (3H, m, nosyl), 7.37 (4H, s, Ar), 6.37 (1H, ddd, *J* 17.2, 10.1, 7.2, alkene-*CH*), 5.76 (1H, m, pentenyl-*CH*), 5.67 (1H, d, *J* 7.05, allyl-*CH*), 5.50 (1H, d, *J* 10.2, alkene-CH_2-H_A), 5.40 (1H, d, *J* 17.2, alkene-CH_2-H_B), 5.01 (2H, td, *J* 10.5, 1.5, pentenyl-CH_2), 4.82 (2H, s, benzyl), 4.51 (2H, s, alkyne-CH_2), 4.16 (1H, d, *J* 17.7, alkyne-CH_2-H_A), 3.88 (1H, d, *J* 17.7, alkyne-CH_2-H_B), 2.64 (2H, m, pentenyl-CH_2-4), 2.33 (2H, q, *J* 7.3, pentenyl-CH_2-3), 2.12 (2H, m, tag-CH_2), 1.15-1.03 (14H, m, tag), 0.91 (2H, m, tag); δ_C (75 MHz; $CDCl_3$) 172.2 (carbonyl), 148.2 (nosyl), 142.0 (Ar), 136.1 (pentenyl), 135.1 (alkene), 134.7 (nosyl), 133.2 (nosyl), 132.7 (Ar), 128.6 (Ar), 126.8 (nosyl), 125.0 (alkene), 116.5 (pentenyl), 79.9 (alkyne), 79.7 (alkyne), 65.7 (allyl), 64.9 (benzyl), 36.6 (alkyne), 35.4 (alkyne), 35.1 (pentenyl), 28.2 (pentenyl), 25.7 (tag), 17.9 (tag), 17.8 (tag), 12.7 (tag), 0.1 (tag); m/z (ES) [M+NH_4] 1207.2 (100%, M+NH_4); HRMS Found: 1207.2247, $C_{42}H_{47}F_{20}N_4O_8S_2Si_1$ requires 1207.2280.

(*S*)-*N*-(But-3-enyl)-2-nitro-*N*-(6-(1,1,1-trifluoro-*N*-(1-(4-(((3,3,4,4,5,5, 6,6,7,7,8,8,9,9,10,10,10-heptadecafluorodecyl)diisopropylsilyloxy)methyl)phenyl)allyl)methylsulfonamido)hexa-2,4-diynyl)benzenesulfonamide (34)

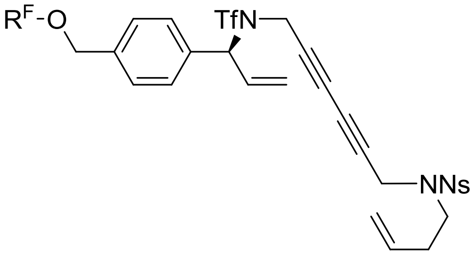

By general method **A4**, the fluorous-tagged alcohol **26** (0.8 g, 0.85 mmol) and the sulfonamide **12b** (0.869 g, 3.38 mmol) gave a crude product, which was purified by F-SPE to afford the metathesis substrate **34** (0.750 g, 76%), R_f 0.39 (20:80, EtOAc:petrol); δ_H (500 MHz; $CDCl_3$) 8.02 (1H, dd, *J* 7.7, 1.7, nosyl), 7.70 (2H, qd, *J* 16.7, 7.7, 1.7, nosyl), 7.64 (1H, dd, *J* 7.7, 1.7, nosyl), 7.36 (2H, d, *J* 8.1, Ar), 7.32 (2H, dd, *J* 8.1, Ar), 6.29 (1H, ddd, *J* 17.1, 10.2, 6.8, alkene-*CH*), 5.70 (2H, m, butenyl-*CH*, allyl-H), 5.50 (1H, d, *J* 10.7, alkene-CH_2-H_A), 5.39 (1H, d, *J* 17.1, alkene-CH_2-H_B), 5.10 (1H, dd, *J* 17.1, 1.2, butenyl-CH_2-H_A), 5.04 (1H, d, *J* 10.2, butenyl-CH_2-H_B), 4.81 (2H, s, benzyl), 4.27 (2H, s, diyne-CH_2), 4.14 (1H, d, *J* 17.5, diyne-CH_2-H_A), 3.83 (1H, d, *J* 18.4, diyne-CH_2-H_B), 3.45 (2H, t, *J* 7.7, butenyl-CH_2-2), 2.34 (2H, q, *J* 7.3, butenyl-CH_2-3), 2.10 (2H, m, tag), 1.13 (2H, m, tag), 1.08, 1.07 (12H, d, *J* 2.1, tag), 0.91 (2H, m, tag); δ_C (75 MHz; $CDCl_3$) 148.5 (nosyl), 142.2 (Ar), 134.3 (butenyl), 134.2 (alkene), 132.9 (Ar), 132.0 (nosyl), 131.2 (nosyl), 128.6 (Ar), 126.8 (Ar), 120.7 (alkene), 124.6 (nosyl), 118.1 (butenyl), 73.1 (diyne), 69.5 (diyne), 69.2 (diyne), 65.6 (allyl), 64.9 (benzyl), 47.0 (butenyl), 37.6 (diyne), 35.8 (diyne), 32.5 (butenyl), 25.7 (tag), 17.9 (tag), 17.8 (tag), 12.7 (tag), 0.1 (tag); ν_{max}/cm^{-1} (film) 2947, 2870, 1738, 1643, 1548; m/z (ES) [M+Na] 1208 (100%, M+Na); HRMS Found: 1208.1890, $C_{43}H_{43}F_{20}N_3Na_1O_7S_2Si_1$ requires 1208.1880.

(*S*)-*N*-(2-Nitrophenylsulfonyl)-*N*-(6-(1,1,1-trifluoro-*N*-(1-(4-(((3,3,4,4,5,5,6,6,7,7,8,8,9,9,10,10,10-heptadecafluorodecyl) diisopropylsilyloxy)methyl)phenyl)allyl)methylsulfonamido)hexa-2,4-diynyl)pent-4-enamide (35)

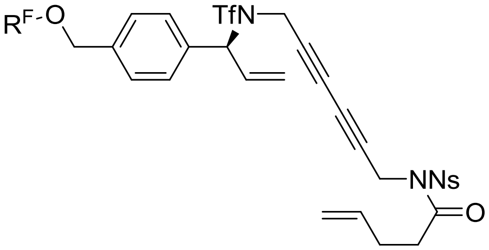

By general method **A4**, the fluorous-tagged alcohol **26** (0.200 g, 0.21 mmol) and the sulfonamide **13** (0.238 g, 0.84 mmol) gave a crude product, which was purified by F-SPE to afford the metathesis substrate **35** (0.190 g, 75%) R_f 0.32 (20:80, EtOAc:petrol); δ_H (500 MHz; $CDCl_3$) 8.40 (1H, m, nosyl), 7.81 (3H, m, nosyl), 7.38 (2H, d, *J* 8.1, Ar), 7.35 (2H, d, *J* 8.1, Ar), 6.32 (1H, ddd, *J* 17.1, 10.2, 7.2, alkene-*CH*), 5.79 (1H, m, pentenyl-*CH*), 5.68 (1H, d, *J* 6.4, allyl-H), 5.52 (1H, d, *J* 10.2, alkene-CH_2-H_A), 5.40 (1H, d, *J* 17.1,

alkene-CH_2-H_B), 5.02 (2H, td, *J* 11.5, pentenyl-CH_2), 4.82 (2H, s, benzyl), 4.66 (2H, s, diyne-CH_2), 4.19 (1H, d, *J* 16.7, diyne-CH_2-H_A), 3.88 (1H, d, *J* 17.5, diyne-CH_2-H_B), 2.74 (2H, t, *J* 7.7, pentenyl-CH_2-3), 2.38 (2H, q, *J* 7.3, pentenyl-CH_2-4), 2.10 (2H, m, tag), 1.13 (2H, m, tag), 1.08, 1.06 (12H, d, *J* 2.9, tag), 0.91 (2H, m, tag); δ_C (75 MHz; $CDCl_3$) 172.1 (carbonyl), 148.2 (nosyl), 142.2 (Ar), 136.2 (pentenyl), 135.4 (alkene), 134.7 (Ar), 132.9 (nosyl), 132.8 (nosyl), 128.7 (Ar), 126.8 (Ar), 125.2 (nosyl), 120.8 (alkene), 116.4 (pentenyl), 73.7 (diyne), 73.3 (diyne), 69.2 (diyne), 69.1 (diyne), 65.6 (allyl), 64.9 (benzyl), 37.1 (diyne), 35.8 (diyne), 35.5 (pentenyl), 28.3 (pentenyl), 25.7 (tag), 17.9 (tag), 17.8 (tag), 12.7 (tag), 0.1 (tag); ν_{max}/cm^{-1} (film) 3025, 2947, 2869, 1715, 1545; m/z (ES) [M+NH_4] 1231.2 (100%, M+NH_4); HRMS Found: 1231.2324, $C_{44}H_{47}F_{20}N_4O_8S_2Si_1$ requires 1231.2280.

***N*-(But-3-enyl)-2-nitro-*N*-((1*S*,4*R*)-4-(1,1,1-trifluoro-*N*-((*S*)-1-(4-(((3,3,4,4,5,5,6,6,7,7, 8,8,9,9,10,10,10-heptadecafluorodecyl)diisopropylsilyloxy)methyl)phenyl)allyl)methylsulfonamido)cyclopent-2-enyl)benzenesulfonamide (36)**

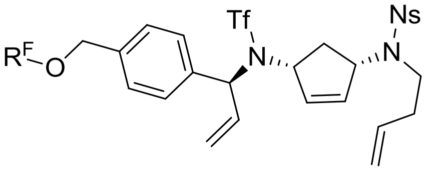

By general method **A5**, the fluorous-tagged alcohol **27** (0.600 g, 0.64 mmol) and the sulfonamide **12b** (0.655 g, 2.56 mmol) gave a crude product, which was purified by F-SPE followed by flash chromatography (gradient elution: 10:90 → 30:70, ethyl acetate–petrol) to afford the metathesis substrate **36** (0.460 g, 62%), R_f 0.33 (20:80, EtOAc:petrol); δ_H (500 MHz; $CDCl_3$) 7.97 (1H, dd, *J* 7.7, 1.5 , nosyl), 7.66 (1H, dq, *J* 7.7, 1.5, nosyl), 7.64 (1H, dq, *J* 7.7, 1.5, nosyl), 7.57 (1H, dd, *J* 7.7, 1.5, nosyl), 7.38 (2H, d, *J* 8.3, Ar), 7.36 (2H, d, *J* 8.3, Ar), 6.10 (1H, ddd, *J* 17.1, 10.6, 6.2, 3-H), 5.95 (1H, d, *J* 5.3, 6-H), 5.69 (1H, dt, *J* 5.8, 2.1, 7-H), 5.62 (1H, m, 13-H), 5.62 (1H, d, *J* 6.2, 2-H), 5.52 (1H, d, *J* 10.6, 4-H_A), 5.38 (1H, d, *J* 17.1, 4-H_B), 4.98 (1H, d, *J* 4.5, 14-H_A), 4.96 (1H, s, 14-H_B), 4.74 (1H, t, *J* 7.7, 5-H), 4.30 (1H, t, *J* 7.7, 8-H), 3.22 (2H, m, 11-H), 2.30 (1H, m, 12-H_A), 2.21 (1H, m, 12-H_B), 2.13 (2H, m, tag), 1.80 (2H, m, 9-H), 1.14 (2H, m, tag), 1.09, 1.08 (12H, d, *J* 3.2, tag), 0.93 (2H, m, tag); δ_C (75 MHz; $CDCl_3$) 148.3 (nosyl), 142.0 (Ar), 136.3 (Ar), 134.6 (nosyl), 133.9 (C-8), 133.8 (C-7), 133.1 (C-13), 133.2 (C-3), 131.9 (nosyl), 131.2 (nosyl), 128.5 (Ar), 126.7 (Ar), 124.5 (nosyl), 119.7 (C-4), 117.5 (C-14), 65.2 (C-2), 64.9 (benzyl), 63.0 (C-8), 62.3 (C-5), 43.9 (C-11), 35.6 (C-12), 25.7 (tag), 17.9 (tag), 17.8 (tag), 12.7 (tag), 0.2 (tag).

***N*-(2-Nitrophenylsulfonyl)-*N*-((1*S*, 4*R*)-4-(1,1,1-trifluoro-*N*-((*S*)-1-(4-(((3,3,4,4,5,5,6,6,7,7,8,8,9,9, 10,10,10-heptadecafluorodecyl)diisopropylsilyloxy)methyl)phenyl)allyl)methylsulfonamido)cyclopent-2-enyl)pent-4-enamide (37)**

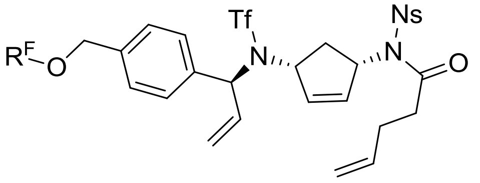

By general method **A5**, the fluorous-tagged alcohol **27** (0.185 g, 0.200 mmol) and the sulfonamide **13** (0.224 g, 0.79 mmol) gave a crude product, which was purified by F-SPE followed by flash chromatography (gradient elution: 10:90 → 30:70, ethyl acetate–petrol) to afford the metathesis substrate **37** (0.125 g, 54%), R_f 0.31 (20:80, EtOAc:petrol); δ_H (500 MHz; $CDCl_3$) 8.20 (1H, m, nosyl), 7.69 (3H, m, nosyl), 7.37 (2H, d, *J* 8.3, Ar), 7.34 (2H, d, *J* 8.3, Ar), 6.19 (1H, ddd, *J* 17.1, 10.2, 6.4, 3-H), 5.96 (2H, m,), 5.71 (1H, m, 13-H), 5.50 (1H, d, *J* 10.2, 4-H_A), 5.41 (1H, m,), 5.35 (1H, d, *J* 17.1, 4-H_B), 5.05 (1H, d, *J* 17.1, 14-H_A), 4.99 (1H, d, *J* 9.4, 14-H_B), 4.81 (2H, s, benzyl), 4.62 (1H, m,), 2.95 (2H, q, *J* 7.2), 2.40 (2H, m,), 2.26 (1H, s,), 2.12 (2H, m, tag), 1.94 (1H, dt, *J* 14.5, 3.6), 1.07 (15H, m, tag), 0.01 (2H, m, tag); δ_C (75 MHz; $CDCl_3$) 176 (carbonyl), 148.0, 141.6, 138.0, 135.9, 135.0, 133.8, 132.5, 130.0, 126.6, 126.5, 124.9, 120.4, 116.7, 81.2, 65.2, 64.9, 64.7, 63.3, 34.3, 29.9, 25.7, 17.8, 17.7, 12.7, 0.1.

***N*-(((1*S*,4*R*)-4-((*N*-(But-3-enyl)-1,1,1-trifluoromethylsulfonamido)methyl)cyclopent-2-enyl)methyl)-*N*-((*S*)-1-(4-(((3,3,4,4,5,5,6,6,7,7,8,8,9,9,10,10,10-heptadecafluorodecyl)diisopropylsilyloxy)methyl)-phenyl)allyl)-2-nitrobenzene sulfonamide (38)**

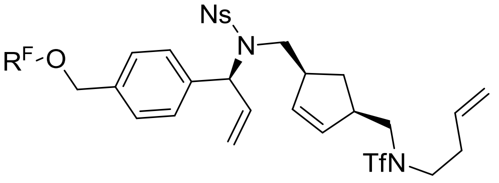

By general method **A5**, the fluorous-tagged alcohol **28** (0.200 g, 0.19 mmol) and the sulfonamide **12a** (0.153 g, 0.76 mmol) gave a crude product, which was purified by F-SPE followed by flash chromatography (gradient elution: 10:90 → 20:80, ethyl acetate–petrol) to afford the metathesis substrate **38** (0.197 g, 86%; ca. 75:25 mixture of diastereomers), R_f 0.42 (20:80, EtOAc:petrol); δ_H (500 MHz; $CDCl_3$) 7.94 (1H, dd, *J* 7.9, 1.3 , nosyl), 7.65 (1H, dd, *J* 7.9, 1.3, nosyl), 7.59 (2H, m, nosyl), 7.32 (2H, dd, *J* 8.3, Ar), 7.26 (2H, d, *J* 8.3, Ar), 6.08 (1H, ddd, *J* 17.3, 10.2, 6.8, 3-H), 5.72 (1H, ddd, *J* 17.1, 12.8, 6.6, 14-H), 5.66 (1H, d, *J* 6.8, 2-H), 5.53 (1H, bs, 7-H), 5.36 (1H, dt, *J* 5.7, 2.1, 8-H), 5.23 (1H, d, *J* 10.2, 4-H_A), 5.12 (3H, m, 4-H_B, 15-H), 4.76 (2H, s, benzyl), 3.40 (2H, m, 12-H), 3.34-3.20 (4H, m, 10, 5-H), 2.87 (1H, quin, *J* 16.0, 8.6, 2.1, 9-H), 2.60 (1H, bs, 6-H), 2.38 (2H, quin, *J* 15.6, 8.5, 13-H), 2.12 (2H, m, tag), 1.99 (1H, dt, *J* 21.8, 6.8, 11-H_A), 1.24 (1H, quin, *J* 6.8, 11-H_B), 1.13-1.05 (14H, m, tag), 0.91 (2H, m, tag); δ_C (75 MHz; $CDCl_3$) 148.5 (nosyl), 141.3 (nosyl), 137.2 (Ar), 134.1 (Ar), 133.9 (C-14), 133.8 (C-7), 133.7 (C-8), 131.7 (nosyl), 131.3 (nosyl), 128.8 (Ar), 126.4 (C-3), 126.3 (Ar), 124.3 (nosyl), 120.0 (C-4), 118.4 (C-15), 65.0 (benzyl), 63.4 (C-2), 54.1 (C-10), 51.5 (C-5), 49.0 (C-12), 46.3 (C-6), 44.7 (C-9), 33.3 (C-13), 32.9 (C-11), 25.7 (tag), 17.8 (tag), 17.7 (tag), 12.7 (tag), 0.2 (tag); ν_{max}/cm^{-1} (film) 3048, 2947, 2869, 1547, 1513; m/z (ES) [M+NH_4] 1221.3 (100%, M+NH_4); HRMS Found: 1221.2826, $C_{44}H_{53}F_{20}N_4O_7S_2Si_1$ requires 1221.2806.

***N*-(((1*R*,4*S*)-4-((*N*-((*S*)-1-(4-(((3,3,4,4,5,5,6,6,7,7,8,8,9,9,10,10,10-Heptadecafluoro decyl)diisopropyl-silyloxy)methyl)phenyl)allyl)-2-nitrophenylsulfonamido)methyl)cyclopent-2-enyl)methyl)-*N*-(2-nitro-phenylsulfonyl)pent-4-enamide (39)**

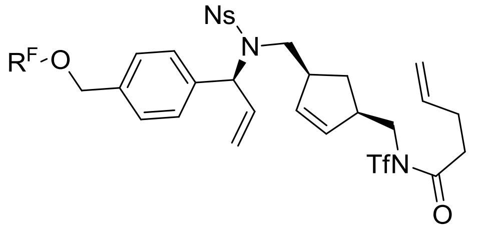

By general method **M5**, the fluorous-tagged alcohol **28** (0.200 g, 0.20 mmol) and the sulfonamide **13** (0.223 g, 0.80 mmol) gave a crude product after 6 hr, which was purified by F-SPE followed by flash chromatography (gradient elution: 10:90 → 20:80, ethyl acetate–petrol) to afford the metathesis substrate **39** (0.180 g, 77%; ca. 75:25 mixture of diastereomers), R_f 0.62 (40:60, EtOAc:petrol); δ_H (500 MHz; $CDCl_3$) 8.36 (1H, m, nosyl^{maj}), 8.25 (1H, m, nosyl^{min}), 7.92 (2H, m, nosyl), 7.80-7.55 (8H, m, nosyl$^{maj\&min}$), 7.32 (2H, d, *J* 8.1, Ar^{maj}), 7.25 (2H, d, *J* 8.1, Ar^{min}), 6.09 (2H, m, 3-H), 5.82 (1H, m, 15-H^{min}), 5.72 (1H, m, 16-H), 5.66 (2H, d, *J* 6.6, 2-H), 5.63 (1H, m, 8-H^{maj}), 5.46 (1H, dt, *J* 5.5, 1.9, 8-H^{min}), 5.41 (1H, td, *J* 5.5, 1.9, 7-H^{maj}), 5.32 (1H, td, *J* 5.5, 1.9, 7-H^{min}), 5.25 (1H, d, *J* 10.6, 4-H_A^{maj}), 5.16 (2H, d, *J* 9.4, 4-H_A^{min}), 5.11 (1H, d, *J* 17.1, 4-H_B^{maj}), 5.10 (1H, d, *J* 17.1, 4-H_B^{min}), 4.95 (1H, dd, *J* 17.1, 1.9, 17-H_A^{maj}), 4.92 (1H, dq, *J* 10.3, 3.0, 1.3, 17-H_B^{maj}), 4.78 (2H, s, benzyl^{maj}), 4.76 (2H, s, benzyl^{min}), 3.93 (2H, dq, *J* 17.3, 10.9, 6.8, 10-H^{min}), 3.77 (1H, dd, *J* 15.1, 5.5, 10-H_A^{maj}), 3.70 (1H, dd, *J* 15.1, 8.3, 10-H_B^{maj}), 3.37 (2H, q, *J* 8.7, 5-H^{min}), 3.31 (2H, q, *J* 8.4, 5-H^{maj}), 3.00 (3H, m, 14-H^{min}, 9-H^{maj}), 2.86 (1H, m, 9-H^{min}), 2.60 (2H, t, *J* 7, 14-H^{maj}), 2.53 (1H, s, 7-H), 2.59 (1H, m, 7-H^{maj}), 2.46 (2H, q, *J* 7, 15-H^{min}), 2.30 (2H, q, *J* 7, 15-H^{maj}), 2.11 (4H, m, tag), 1.99 (2H, m, 11-$H_A^{maj\&min}$), 1.29 (1H, quin, *J* 6.8, 11-H_B^{maj}), 1.19 (1H, quin, *J* 6.8, 11-H_B^{min}), 1.11-1.01 (28H, m, tag), 0.89 (4H, m, tag); δ_C (75 MHz; $CDCl_3$) 176.7 (carbonyl^{min}), 172.8 (carbonyl^{maj}), 148.5 (nosyl), 141.4 (nosyl^{min}), 141.3 (nosyl^{maj}), 137.2 (Ar), 136.4 (C-16), 136.2 (Ar), 135.2 (Ar), 134.8 (nosyl), 134.6 (nosyl), 134.5 (nosyl), 134.1, 134.0 (C-7^{min}), 133.6 (C-7^{maj}), 133.5, 132.5 (C-8^{maj}), 132.4, 132.2, 132.0 (C-8^{min}), 131.8, 131.4, 131.3, 129.9, 128.8, 128.7, 126.4, 126.4, 126.3, 124.8, 124.3, 124.2 (nosyl), 120.0 (C-4^{maj}), 119.8 (C-4^{min}), 116.6 (17-C^{min}), 116.2 (17-C^{maj}), 72.8 (benzyl), 65.0 (C-10), 63.4 (C-2), 63.3 (C-2^{min}), 52.3 (C-5^{maj}), 51.7 (C-5^{min}), 46.6 (C-6^{maj}), 46.3 (C-9^{maj}), 46.0 (C-6^{min}); ν_{max}/cm^{-1} (film) 3095, 2947, 2869, 1704, 1642, 1592, 1538; m/z (ES) [M+NH_4] 1302.3 (100%, M+NH_4); HRMS Found: 1307.2604, $C_{50}H_{53}F_{17}N_4Na_1O_{10}S_2Si_1$ requires 1307.2593.

(*R*)-*N*-(4-(*N*-(But-3-enyl)-1,1,1-trifluoromethylsulfonamido)but-2-ynyl)-*N*-(1-((3,3,4,4,5,5,6,6,7,7,8,8, 9,9,10,10,10-heptadecafluorodecyl)diisopropylsilyloxy)pent-4-en-2-yl)-2-nitrobenzenesulfonamide (40)

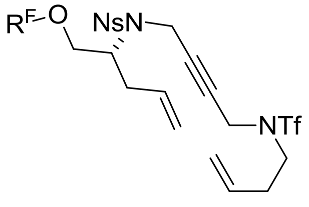

By general method **A6**, the fluorous-tagged alcohol **29** (1.05 g, 1.09 mmol) and the sulfonamide **12a** (0.888 g, 4.3 mmol) gave a crude product, which was purified by F-SPE followed by flash chromatography (gradient elution: 10:90 → 20:80, ethyl acetate–petrol) to afford the metathesis substrate **40** (1.015 g, 86%), R_f 0.41 (20:80, EtOAc:petrol); δ_H (500 MHz; $CDCl_3$) 8.10 (1H, dd, *J* 7.5, 1.7, nosyl), 7.68 (3H, m, nosyl), 5.71 (1H, m, 3''-H butene), 5.64 (1H, m, 4-H), 5.13 (1H, qd, *J* 13.3, 1.5, 4''-H_A butene), 5.11 (1H, dd, *J* 6.2, 1.5, 4''-H_B butene), 5.06 (1H, dd, *J* 17.3, 1.5, 5-H_A), 4.97 (1H, d, *J* 10.2, 5-H_B), 4.37 (1H, qd, *J* 18.8, 2.1, 4'-H_A alkyne), 4.31 (1H, qd, *J* 18.8, 2.1, 4'-H_B alkyne), 4.07 (2H, s, 1'-CH_2 alkyne), 3.96 (1H, m, 2-H), 3.84 (2H, m, 1-CH_2), 3.47 (2H, s, 1''-CH_2 butene), 2.51 (2H, m, 3-CH_2), 2.35 (2H, q, *J* 7.2, 2''-CH_2 butene), 2.08 (2H, m, tag), 1.02 (14H, s, tag), 0.82 (2H, m, tag); δ_C (75 MHz; $CDCl_3$) 148.3 (nosyl), 134.2 (C-3'' butene), 134.1 (nosyl), 134.0 (nosyl), 133.9 (C-4), 133.5 (nosyl), 132.0 (nosyl), 131.8 (nosyl), 124.5 (nosyl), 118.6 (C-5), 118.5 (C-4'' butene), 82.9 (C-2' alkyne), 76.6 (C-3' alkyne), 64.9 (C-1), 59.7 (C-2), 47.3 (C-1''), 37.8 (C-1'), 34.4 (C-3), 34.2 (C-4' alkyne), 32.5 (C-2'' butene), 25.6 (tag), 17.7 (tag), 17.6 (tag), 12.4 (tag), 0.1 (tag); ν_{max}/cm^{-1} (film) 3691, 2948, 2871, 1548, 1463, 1393; m/z (ES) [M+NH_4] 1117.2 (100%, M+NH_4); HRMS Found: 1117.2214, $C_{36}H_{45}F_{20}N_4O_8S_2Si_1$ requires 1117.2124.

(*R*)-*N*-(4-(*N*-(1-((3,3,4,4,5,5,6,6,7,7,8,8,9,9,10,10,10-Heptadecafluorodecyl)diisopropylsilyloxy)pent-4-en-2-yl)-2-nitrophenylsulfonamido)but-2-ynyl)-*N*-(2-nitrophenylsulfonyl)pent-4-enamide (41)

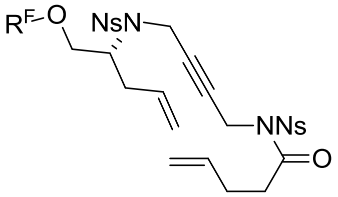

By general method **A6**, the fluorous-tagged alcohol **29** (0.450 g, 0.5 mmol) and the sulfonamide **13** (0.560 g, 2.0 mmol) gave a crude product, which was purified by F-SPE followed by flash chromatography (gradient elution: 10:90 → 20:80, ethyl acetate–petrol) to afford the metathesis substrate **41** (0.450 g, 77%) R_f 0.37 (30:70, EtOAc:petrol); δ_H (500 MHz; $CDCl_3$) 8.39 (1H, d, *J* 6.2, nosyl), 8.12 (1H, dd, *J* 7.3, 1.0, nosyl), 7.78 (3H, m, nosyl), 7.71 (2H, dq, *J* 7.3, 1.0, nosyl), 7.62 (1H, d, *J* 7.3, 1.0 , nosyl), 5.74 (1H, m, 4''-H pentene), 5.65 (1H, m, 4-H), 5.08 (1H, d, *J* 16.6, 5-H_A), 5.00 (1H, d, *J* 10.9, 5-H_B), 4.98 (1H, s, 5''-H_A pentene), 4.96 (1H, d, *J* 4.7, 5''-H_B pentene), 4.48 (2H, s, 1'-CH_2 alkyne), 4.32 (2H, s, 4'-CH_2 akyne), 3.98 (1H, m, 2-H), 3.85 (2H, d, *J* 5.2, 1-CH_2), 2.64 (2H, td, *J* 7.3, 2.6, 2''-CH_2 pentene), 2.51 (2H, m, 3-CH_2), 2.31 (2H, q, *J* 7.3, 3''-CH_2 pentene), 2.09 (2H, m, tag), 1.03 (14H, m, tag), 0.82 (2H, m, tag); δ_C (75 MHz; $CDCl_3$) 172.1 (carbonyl), 148.3 (nosyl), 136.2 (C-4'' pentene), 135.2 (nosyl), 134.4 (nosyl), 134.2 (nosyl), 134.1 (nosyl), 134.0 (nosyl), 133.1 (nosyl), 132.7 (nosyl), 132.3 (nosyl), 131.6 (nosyl), 125.0 (nosyl), 124.5 (nosyl), 118.5 (C-5), 116.4 (C-5'' pentene), 81.5 (C-2' alkyne), 80.0 (C-3' alkyne), 64.8 (C-1), 59.7 (C-2), 36.6 (C-1' alkyne), 35.3 (C-2'' pentene), 34.4 (C-4' alkyne), 34.3 (C-3), 28.2 (C-3'' pentene), 25.6 (tag), 17.7 (tag), 17.6 (tag), 12.4 (tag), 0.1 (tag); ν_{max}/cm^{-1} (film) 3687, 2947, 2869, 1717, 1545, 1464; m/z (ES) [M+NH_4] 1198.2 (100%, M+NH_4); HRMS Found: 1203.1979, $C_{42}H_{45}F_{17}N_4Na_1O_{10}S_2Si_1$ requires 1203.1967.

(*R*)-*N*-(6-(*N*-(But-3-enyl)-1,1,1-trifluoromethylsulfonamido)hexa-2,4-diynyl)-*N*-(1-((3,3,4,4,5,5,6,6,7,7,8,8,9,9,10,10,10-heptadecafluorodecyl)diisopropylsilyloxy)pent-4-en-2-yl)-2-nitrobenzenesulfonamide (42)

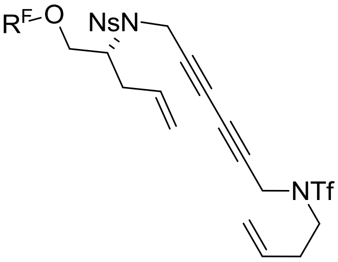

By general method **A6**, the fluorous-tagged alcohol **30** (0.900 g, 1.9 mmol) and the sulfonamide **12a** (0.771 g, 3.8 mmol) gave a crude product, which was purified by F-SPE followed by flash chromatography (gradient elution: 10:90 → 20:80, ethyl acetate–petrol) to afford the metathesis substrate **42** (0.970 g, 92%) R_f 0.53 (20:80, EtOAc:petrol); δ_H (500 MHz; $CDCl_3$) 8.13 (1H, d, *J* 7.4, nosyl), 7.67 (3H, m, nosyl), 5.74 (1H, m, 3"-H butene), 5.62 (1H, m, 4-H), 5.17 (1H, d, *J* 12.4, , 4"-H_A butene), 5.14 (1H, d, *J* 5.6, 4"-H_B butene), 5.06 (1H, d, *J* 17.1, 5-H_A), 4.97 (1H, d, *J* 10.1, 5-H_B), 4.39 (2H, ABq, *J* 19.2, 6'-H diyne), 4.23 (2H, s, 1'-H diyne), 4.02 (1H, m, 2-H), 3.88 (2H, d, *J* 5.1, 1-CH_2), 3.52 (2H, s, 1"-CH_2 butene), 2.48 (2H, m, 3-CH_2), 2.40 (2H, q, *J* 7.2, 2"-CH_2 butene), 2.08 (2H, m, tag), 1.03 (14H, s, tag), 0.83 (2H, m, tag); δ_C (75 MHz; $CDCl_3$) 148.3 (nosyl), 134.1 (C-4), 134.0 (nosyl), 133.9 (nosyl), 133.4 (C-3" butene), 131.9 (nosyl), 124.6 (nosyl), 118.9 (C-4" butene), 118.6 (C-5), 75.9 (C-5' diyne), 71.1 (C-3' diyne), 70.3 (C-4' diyne), 68.3 (C-2' diyne), 65.1 (C-1), 59.6 (C-2), 47.6 (C-1" butene), 38.4 (C-1' diyne), 34.8 (C-6' diyne), 34.2 (C-3), 32.6 (C-2" butene), 25.6 (tag), 17.7 (tag), 17.6 (tag), 12.5 (tag), 0.1 (tag); ν_{max}/cm^{-1} (film) 3083, 2947, 2870, 1644, 1548, 1463; m/z (ES) [M+NH_4] 1141.2 (100%, M+NH_4); HRMS Found: 1146.176, $C_{38}H_{41}F_{20}N_3Na_1O_7S_2Si_1$ requires 1146.1728.

(*R*)-*N*-(6-(*N*-(1-((3,3,4,4,5,5,6,6,7,7,8,8,9,9,10,10,10-Heptadecafluorodecyl)diisopropylsilyloxy)pent-4-en-2-yl)-2-nitrophenylsulfonamido)hexa-2,4-diynyl)-*N*-(2-nitrophenylsulfonyl)pent-4-enamide (43)

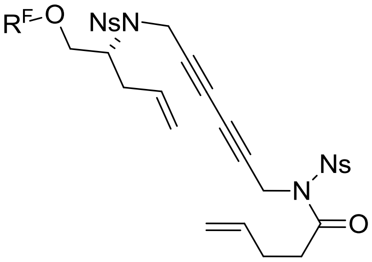

By general method **A6**, the fluorous-tagged alcohol **30** (0.4 g, 0.43 mmol) and the sulfonamide **13** (0.490 g, 1.7 mmol) gave a crude product, which was purified by F-SPE followed by flash chromatography (gradient elution: 10:90 → 20:80, ethyl acetate–petrol) to afford the metathesis substrate **43** (0.284 g, 55%) R_f 0.34 (30:70, EtOAc:petrol); δ_H (500 MHz; $CDCl_3$) 8.40 (1H, m, nosyl), 8.14 (1H, m, nosyl), 7.81 (3H, m, nosyl), 7.72 (2H, m, nosyl), 7.64 (1H, m, nosyl), 5.77 (1H, m, 4"-H pentene), 5.68 (1H, m, 4-H), 5.09 (1H, d, *J* 17.1, 5-H_A), 5.03 (1H, m, 4"-H_A pentene), 5.00 (1H, s, 4"-H_B pentene), 4.99 (1H, d, *J* 10.1, 5-H_B), 4.61 (2H, s, 1'-CH_2 diyne), 4.37 (2H, ABq, *J* 19.2, 6'-CH_2 diyne), 4.07 (1H, m, 2-H), 3.86 (2H, t, *J* 4.9, 1-H), 2.70 (2H, t, *J*

7.2, 2"-H pentene), 2.48 (2H, m, 3-CH_2), 2.37 (2H, q, *J* 7.2, 3"-CH_2 pentene), 2.09 (2H, m, tag), 1.02 (14H, s, tag), 0.84 (2H, m, tag); δ_C (75 MHz; $CDCl_3$) 172.0 (carbonyl), 148.2 (nosyl), 136.1 (C-4" pentene), 135.4 (nosyl), 134.7 (nosyl), 134.4 (nosyl), 134.1 (C-4), 133.9 (nosyl), 132.9 (nosyl), 132.7 (nosyl), 132.1 (nosyl), 132.0 (nosyl), 125.2 (nosyl), 124.5 (nosyl), 118.7 (C-5), 116.5 (C-5" pentene), 75.6 (C-2' diyne), 72.9 (C-5' diyne), 69.1 (C-3' diyne), 68.7 (C-4' diyne), 65.1 (C-1), 59.5 (C-2), 37.1 (C-1' diyne), 35.4 (C-2" pentene), 34.8 (C-6' diyne), 34.2 (C-3), 28.3 (C-3" pentene), 25.6 (tag), 17.7 (tag), 17.6 (tag), 12.5 (tag), 12.4 (tag), 0.1 (tag); ν_{max}/cm^{-1} (film) 3082, 2947, 2869, 1715, 1547, 1368; m/z (ES) [M+NH_4] 1222.3 (100%, M+NH_4); HRMS Found: 1222.2477, $C_{44}H_{49}F_{17}N_5O_{10}S_2Si_1$ requires 1222.2413.

***N*-((1*R*,4*S*)-4-(*N*-(But-3-enyl)-1,1,1-trifluoromethylsulfonamido)cyclopent-2-enyl)-*N*-((*R*)-1-((3,3,4,4,5,5,6,6,7,7,8,8,9,9,10,10,10-heptadecafluorodecyl)diisopropylsilyloxy)pent-4-en-2-yl)-2-nitrobenzenesulfonamide (44)**

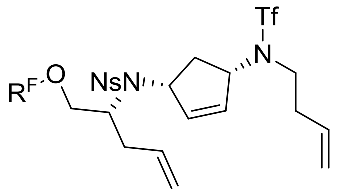

By general method **A6**, the fluorous-tagged alcohol **31** (1.00 g, 1.07 mmol) and the sulfonamide **12a** (1.1 g, 4.31 mmol) gave a crude product, which was purified by F-SPE to afford the metathesis substrate **44** (1.008 g, 85%) R_f 0.46 (20:80, EtOAc:petrol); δ_H (500 MHz; $CDCl_3$) 8.06 (1H, d, *J* 7.7, nosyl), 7.70 (1H, dt, *J* 7.7, 1.5, nosyl), 7.65 (1H, dt, *J* 7.7, 1.5, nosyl), 7.59 (1H, dd, *J* 7.7, 1.5, nosyl), 5.95 (1H, s, 2'-H cyclopentene), 5.80 (1H, d, *J* 4.7, 3'-H cyclopentene), 5.69 (1H, m, 4-H,), 5.08 (4H, m, 5-CH_2, 4"-H_A butene), 4.84 (1H, t, *J* 7.7, 1'-H cyclopetene), 4.70 (1H, dt, *J* 8.3, 2.5, 4'-H cyclopentene), 3.92 (1H, dd, *J* 10.9, 5.7, 1-H_A), 3.86 (1H, dd, *J* 10.9, 4.2, 1-H_B), 3.75 (1H, bs, 2-H), 3.42 (2H, t, *J* 8.3, 1"-H butene), 2.74 (1H, quin, *J* 7.7, 5'-H_A cyclopentene), 2.50 (5H, m, 3-H, 2"-H butene, 5'-H_B cyclopentene), 2.25 (1H, m, 2"-H butene), 2.05 (2H, m, tag), 1.07 (14H, m, tag), 0.82 (2H, m, tag); δ_C (75 MHz; $CDCl_3$) 148.9 (nosyl), 135.9 (C-2' cyclopentene), 134.4 (C-3" butene), 134.3 (C-4), 133.6 (nosyl), 133.8 (nosyl), 131.7 (nosyl), 131.2 (C-3' cyclopentene), 130.8 (nosyl), 124.4 (nosyl), 118.4 (C-5), 118.0 (C-4" butene), 64.8 (C-1), 63.6 (C-1' cyclopentene), 61.5 (C-4' cyclopentene), 60.2 (C-2), 45.3 (C-1" butene), 36.4 (C-5' cyclopentene), 35.9 (C-3, C-2" butene), 25.6 (tag), 17.7 (tag), 17.6 (tag), 12.4 (tag), 0.1 (tag); ν_{max}/cm^{-1} (film) 3422, 2949, 2870, 1643, 1548, 1462; m/z (ES) [M+NH_4] 1131.2 (100%, M+NH_4); HRMS Found: 1131.2378, $C_{37}H_{43}F_{20}N_3Na_1O_7S_2Si_1$ requires 1131.1885.

5 Synthesis of metathesis products

(*S*)-5-(5-(4-(((3,3,4,4,5,5,6,6,7,7,8,8,9,9,10,10,10-Heptadecafluorodecyl)diisopropylsilyloxy)methyl)-phenyl)-1-(trifluoromethylsulfonyl)-2,5-dihydro-1*H*-pyrrol-3-yl)-1,2,3,6-tetrahydropyridine (45)

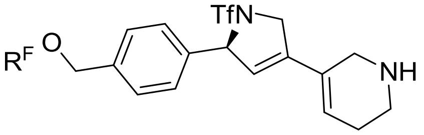

By using general method **B1**, the metathesis precursor **32** (0.880 g, 0.76 mmol) and catalyst HG-II (5 mol % then 2.5 mol % after 48 hr) gave a crude product, which was purified by flash chromatography (gradient elution: 10:90 → 40:60, ethyl acetate–petrol) to afford a *ca* 70:20:10 mixture (0.498 g) of the diene and diastereomeric cyclopropane products. By general method **C**, a portion of this mixture (0.350 g) gave a crude product that was purified by F–SPE, eluting with 80:20 MeOH–H_2O then with MeOH, and flash chromatography (gradient elution: 50:50 → 00:100, ethyl acetate–petrol then 2:98 → 10:90, MeOH–$CHCl_3$) to furnish the *diene* **45** (193 mg, 37% over two steps), R_f 0.44 (20:80, MeOH:$CHCl_3$); δ_H (500 MHz; $CDCl_3$) 7.29 (2H, d, *J* 8.1, Ar), 7.23 (2H, d, *J* 8.1, Ar), 5.84 (1H, s, 4'-H), 5.76 (1H, s, 5'-H), 5.53 (1H, s, 4-H), 4.78 (2H, s, benzyl-CH_2), 4.68 (1H, d, *J* 12.8, 2'-H_A), 4.55 (1H, d, *J* 12.8, 2'-H_B), 3.52 (2H, s, 6-CH_2), 2.98 (2H, t, *J* 5.5, 2-CH_2), 2.26 (2H, s, 3-CH_2), 2.10 (2H, m, tag), 1.12-1.01 (14H, m, tag), 0.99 (2H, m, tag); δ_C (75 MHz; $CDCl_3$) 141.7 (Ar), 138.0 (Ar), 135.9 (C-3'), 130.2 (C-5), 127.6 (Ar), 127.1 (C-4'), 126.4 (Ar), 121.7 (C-4), 71.8 (C-5'), 64.9 (benzyl), 55.6 (C-2'), 45.1 (C-6), 42.6 (C-2), 26.1 (C-3), 25.6 (tag), 17.7 (tag), 17.6 (tag), 12.6 (tag), 0.1 (tag); m/z (ES) [M+H] 949.2 (100%, M+H); HRMS Found: 949.1953, $C_{33}H_{37}F_{20}N_2O_3S_1Si_1$ requires 949.1969.

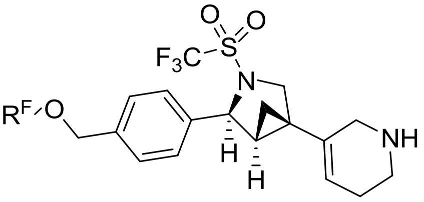

Also obtained was (1*S*,4*S*,5*R*)-4-(4-(((3,3,4,4,5,5,6,6,7,7,8,8,9,9,10,10,10-heptadecafluorodecyl)diisopropylsilyloxy)methyl)phenyl)-1-(1,2,5,6-tetrahydropyridin-3-yl)-3-(trifluoromethylsulfonyl)-3-azabicyclo-[3.1.0]hexane **46** (59 mg, 11% over 2 steps), R_f 0.31 (20:80, MeOH:$CHCl_3$); δ_H (500 MHz; $CDCl_3$) 7.29 (4H, s, Ar), 5.70 (1H, t, *J* 4.2, 4-H), 5.17 (1H, d, *J* 4.2, 6'-H), 4.79 (2H, s, benzyl-CH_2), 3.90 (1H, d, *J* 9.4, 2'-H_A), 3.86 (1H, d, *J* 9.4, 2'-H_B), 3.32 (1H, d, *J* 16.2, 6-H_A), 3.22 (1H, d, *J* 16.2, 6-H_B), 2.92 (2H, q, *J* 9.4, 6.4, 2-CH_2), 2.62 (1H, bs, N-H), 2.12 (4H, m, 3-CH_2, tag), 1.97 (1H, quin, *J* 4.2, 5'-H), 1.20 (1H, t, *J* 5.1, 4'-H_A), 1.09 (14H, m, tag), 0.89 (3H, m, tag, 4'-H_B); δ_C (75 MHz; $CDCl_3$) 141.3 (Ar), 136.8 (C-5), 134.1 (Ar), 127.4 (Ar), 126.1 (Ar), 123.1 (C-4), 66.3 (C-6'), 65.1 (benzyl), 56.4 (C-2'), 45.7 (C-6), 42.7 (C-2), 31.1 (C-5'), 30.9 (C-3'), 25.7 (tag), 25.5 (C-3), 17.9 (tag), 17.8 (tag), 13.7 (C-4'), 12.7 (tag), 0.1 (tag); ν_{max}/cm^{-1} (film) 2948, 2869, 1661, 1515, 1463, 1391; m/z (ES) [M+H] 963.2 (100%, M+H); HRMS Found: 963.2118, $C_{34}H_{39}F_{20}N_2O_3S_1Si_1$ requires 963.2126.

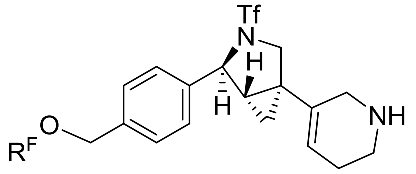

Also obtained was (1*R*,4*S*,5*S*)-4-(4-(((3,3,4,4,5,5,6,6,7,7,8,8,9,9,10,10,10-heptadecafluorodecyl)diisopropylsilyloxy)methyl)phenyl)-1-(1,2,5,6-tetrahydropyridin-3-yl)-3-(trifluoromethyl sulfonyl)-3-azabicyclo-[3.1.0]hexane **47** (28 mg, 5% over two steps), R_f 0.25 (10:90, MeOH:CHCl$_3$); δ_H (500 MHz; CDCl$_3$) 7.29 (2H, dd, *J* 8.1, 2.5, Ar), 7.21 (2H, d, *J* 8.1, Ar), 5.69 (1H, s, 6'-H), 5.40 (1H, t, *J* 2.1, 4-H), 4.78 (2H, s, benzyl), 4.43-4.23 (2H, m, 2'-H), 3.18 (2H, quin, *J* 19.4, 12.4, 6-H), 2.75 (1H, m, 2-H$_A$), 2.51 (1H, m, 2-H$_B$), 2.10 (2H, m, tag), 1.96 (1H, m, 3-H$_A$), 1.73 (1H, m, 3-H$_B$), 1.23 (1H, m, 4'-H$_A$), 1.10 (1H, m, 5'-H), 1.08 (14H, m, tag), 0.97 (1H, m, 4'-H$_B$), 0.89 (2H, m, tag); δ_C (75 MHz; CDCl$_3$) 142.8 (Ar), 141.6 (C-5), 138.2 (Ar), 127.5 (Ar), 126.3 (Ar), 122.2 (C-4), 71.6 (C-6'), 64.9 (benzyl), 56.0 (C-2'), 47.6 (C-6), 42.4 (C-2), 25.5 (tag), 18.7 (C-3'), 18.0 (C-4'), 17.7 (tag), 17.6 (tag), 17.2 (C-5'), 12.6 (tag), 0.1 (tag); ν_{max}/cm^{-1} (film) 2948, 2869, 1647, 1464, 1388; m/z (ES) [M+H] 963.2 (100%, M+H); HRMS Found: 963.2147, $C_{34}H_{39}F_{20}N_2O_3S_1Si_1$ requires 963.2126.

(*S*)-6-(5-(4-(Hydroxymethyl)phenyl)-1-(trifluoromethylsulfonyl)-2,5-dihydro-1*H*-pyrrol-3-yl)-3,4-dihydro-1*H*-azepin-2(7*H*)-one (48)

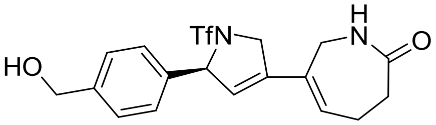

By general method **B1**, the metathesis precursor **33** (0.190 g, 0.16 mmol) and catalyst HG-II (5 mol % then 5 mol % after 48 hr) gave a crude product, which was subjected to flash chromatography (gradient elution: 5:95 → 10:90, ethyl acetate–petrol) to afford a mixture (0.125 g) of products. By general method **C1**, a portion of the mixture(0.110 g) gave a crude product (0.075 g) that was subjected to F–SPE and flash column chromatography (gradient elution: 20:80 → 80:20, ethyl acetate–petrol). By general method **D**, a portion of the resulting mixture (0.040 g) (gradient elution: 02:98 → 10:90, MeOH–CHCl$_3$) gave the *diene* **48** (0.014 g, 43% over three steps), R_f 0.65 (20:80, MeOH:CHCl$_3$); δ_H (500 MHz; CD$_3$OD) 7.36 (2H, d, *J* 8.3, Ar), 7.27 (2H, d, *J* 8.3, Ar), 5.83 (3H, m, 5-H, 4'-H, 5'-H), 4.67 (2H, s, 2'-H), 4.60 (2H, s, benzyl), 4.05 (2H, s, 7-H), 2.72 (2H, dt, *J* 13.4, 5.3 , 4-H), 2.58 (2H, m, 3-H); δ_C (100 MHz; CD$_3$OD) 177.9 (carbonyl), 141.9 (Ar), 136.4 (C-3'), 130.4 (C-5), 130.2 (C-6), 128.0, 126.9, 126.7, 126.3, 122.8 (C-4'), 71.7 (C-5'), 66.0 (benzyl), 55.4 (C-2'), 39.2 (C-7), 31.5 (C-4), 23.8 (C-3).

(*S*)-5-((5-(4-(((3,3,4,4,5,5,6,6,7,7,8,8,9,9,10,10,10-Heptadecafluorodecyl)diisopropyl silyloxy)methyl)-phenyl)-1-(trifluoromethylsulfonyl)-2,5-dihydro-1*H*-pyrrol-3-yl)ethynyl)-1,2,3,6-tetrahydropyridine (49)

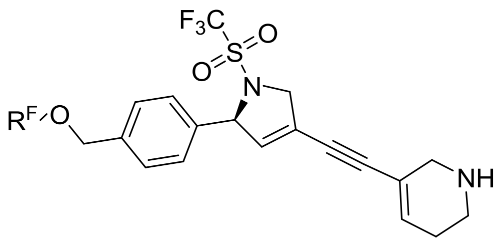

By general method **B1**, the metathesis precursor **20** (0.730 g, 0.62 mmol) and the catalyst HG-II (5 mol % then 5 mol % after 3 days then 2.5 mol % after 5 days) gave a crude product, which was purified by flash chromatography (gradient elution: 5:95 → 10:90, ethyl acetate–petrol) to give a mixture (0.330 g) of products. By general procedure **C1**, a portion of the mixture (0.270 g) gave a crude product, which was purified directly by F–SPE, and then flash chromatography (gradient elution: 50:50 → 80:20, ethyl acetate–petrol), to furnish the *dienyne* **49** (0.185 g, 49% over 2 steps), R_f 0.67 (10:90, MeOH:CHCl$_3$); δ_H (500 MHz; CDCl$_3$) 7.32 (2H, d, *J* 8.3, Ar), 7.26 (2H, d, *J* 8.3, Ar), 6.30 (1H, t, *J* 4.2, 4-H), 5.94 (1H, d, *J* 2.1, 4'-H), 5.78 (1H, d, *J* 2.1, 5'-H), 4.79 (2H, s, benzyl), 4.55 (1H, d, *J* 13.5, 2'-H$_A$), 4.41 (1H, dd, *J* 13.5, 4.2, 2'-H$_B$), 3.42 (2H, s, 6-H), 2.97 (2H, t, *J* 5.2, 2-H), 2.21 (2H, s, 3-H), 2.11 (2H, m, tag), 1.06 (14H, m, tag), 0.90 (2H, m, tag); δ_C (75 MHz; CDCl$_3$) 142.0 (Ar), 137.1 (Ar), 135.6 (C-4), 133.6 (C-4'), 127.6 (Ar), 126.5 (Ar), 120.6 (C-5), 119.8 (C-3'), 93.7 (C-7), 80.7 (C-8), 71.4 (5'-C), 64.9 (benzyl), 57.5 (C-2'), 47.6 (C-6), 41.9 (C-2), 26.0 (C-3'), 25.6 (tag), 17.7 (tag), 17.6 (tag), 12.6 (tag), 0.01 (tag); ν_{max}/cm^{-1} (film) 2948, 2870, 2209, 1464, 1426, 1391; m/z (ES) [M+H] 973.2 (100%, M+H); HRMS Found: 973.1973, $C_{35}H_{37}F_{20}N_2O_3S_1Si_1$ requires 973.1969.

(*S*)-6-(((2*R*,5*S*)-5-(4-(((3,3,4,4,5,5,6,6,7,7,8,8,9,9,10,10,10-Heptadecafluorodecyl) diisopropylsilyloxy)-methyl)phenyl)-1-(trifluoromethylsulfonyl)-2,5-dihydro-1*H*-pyrrol-2-yl)methyl)-1-(2-nitrophenyl-sulfonyl)-1,2,3,6-tetrahydropyridine (S13)

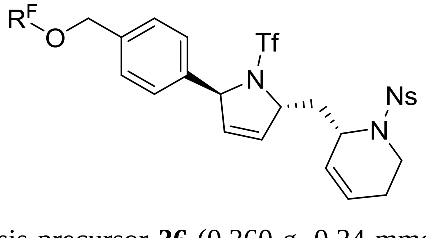

By general method **B1**, the metathesis precursor **36** (0.360 g, 0.34 mmol) and the catalyst HG-II (5 mol % then 3 × 5 mol % portions at 3 day intervals) gave a crude product, which was purified by flash chromatography (gradient elution: 10:90 → 20:80, ethyl acetate–petrol) to afford the cascade product **S13** (0.270 g, 77%) R_f 0.55 (30:70, EtOAc:petrol); δ_H (500 MHz; CDCl$_3$) 8.04 (1H, dd, *J* 7.7, 1.5, nosyl), 7.67 (2H, dq, *J* 7.7, 1.5, nosyl), 7.57 (1H, d, *J* 7.7, 1.5, nosyl), 7.30 (2H, d, *J* 8.3, Ar), 7.26 (2H, d, *J* 8.3, Ar), 6.36 (1H, dt, *J* 8.3, 1.5, 4'-H), 5.74 (1H, dt, *J* 8.2, 1.7, 3'-H), 5.69 (1H, dq, *J* 16.2, 12.4, 1.7, 5-H), 5.65 (1H, dq, *J* 16.2, 12.4, 1.7, 4-H), 5.60 (1H, d, *J* 8.3, 5'-H), 4.79 (2H, s, benzyl), 4.59 (1H, d, *J* 11.3, 6-H), 4.00 (1H, dd, *J* 15.3, 5.5, 2-H$_A$), 3.40 (1H, ddd, *J* 15.3, 11.3, 5.5, 2-H$_B$), 2.79 (1H, t, *J* 13.2, 2'-H), 2.10 (2H, m, tag), 1.90 (4H, m, 3, 7-H), 1.09 (14H, m, tag), 0.90 (2H, m, tag); δ_C (75 MHz; CDCl$_3$) 148.5 (nosyl), 141.9 (Ar), 134.0 (nosyl), 131.8 (nosyl), 130.7 (nosyl), 129.7 (C-4), 127.8 (C-4'), 127.4 (C-5), 127.9 (Ar), 126.1 (Ar), 125.0

(C-3'), 125.5 (nosyl), 72.1 (C-5'), 65.0 (benzyl), 51.7 (C-6), 38.4 (C-2), 39.0 (C-2'), 25.7 (tag), 23.0 (C-3, 7), 17.8 (tag), 17.7 (tag), 12.7 (tag), 0.1 (tag); m/z (ES) [M+H] 1165.2 (100%, $M+NH_4$); HRMS Found: 1170.1758, $C_{40}H_{41}F_{20}N_3Na_1O_7S_2Si_1$ requires 1170.1728.

(*S*)-6-(((2*R*,5*S*)-5-(4-(((3,3,4,4,5,5,6,6,7,7,8,8,9,9,10,10,10-Heptadecafluorodecyl)diisopropylsilyloxy)-methyl)phenyl)-1-(trifluoromethylsulfonyl)-2,5-dihydro-1*H*-pyrrol-2-yl)methyl)-1,2,3,6-tetrahydro-pyridine (50)

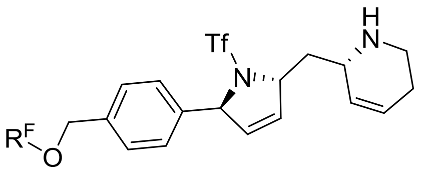

By general method **C1**, the fluorous-tagged sulfonamide **S13** (0.255 g, 0.217 mmol) gave a crude product that was purified by F–SPE and flash column chromatography (gradient elution: 2:98 → 10:90, MeOH–$CHCl_3$) to furnish the amine **50** (170 mg, 81%). R_f 0.42 (10:90, MeOH:$CHCl_3$); δ_H (500 MHz; $CDCl_3$) 7.29 (2H, d, *J* 8.1, Ar), 7.25 (2H, d, *J* 8.1, Ar), 6.20 (1H, d, *J* 6.4, 4'-H), 5.84 (1H, m, 4-H), 5.70 (1H, d, *J* 6.4, 3'-H), 5.63 (1H, s, 5'-H), 5.59 (1H, dd, *J* 9.8, 2.1, 5-H), 5.14 (1H, bs, 2'-H), 4.78 (2H, s, benzyl), 3.45 (1H, dd, *J* 9.8, 2.1, 6-H), 3.09 (1H, dt, *J* 12.8, 5.1, 3-H_A), 2.85 (1H, ddd, *J* 12.8, 8.5, 4.7, 3-H_B), 2.45 (1H, dt, *J* 12.8, 2.9, 7-H_A), 2.09 (3H, m, 3-H_A, tag), 1.96 (1H, d, *J* 12.4, 3-H_B), 1.76 (1H, m, 7-H_B), 1.05 (14H, m, tag), 0.89 (2H, m, tag); m/z (ES) [M+H] 963.2 (100%, M+H); HRMS Found: 963.2166, $C_{34}H_{39}F_{20}N_2O_3S_1Si_1$ requires 963.2126.

(*R*)-3-(((3*S*,6*S*)-6-(4-(((3,3,4,4,5,5,6,6,7,7,8,8,9,9,10,10,10-Heptadecafluorodecyl)diisopropylsilyloxy)-methyl)phenyl)-1-(2-nitrophenylsulfonyl)-1,2,3,6-tetrahydropyridin-3-yl)methyl)-1-(trifluoromethyl-sulfonyl)-2,3,6,7-tetrahydro-1*H*-azepine (S14)

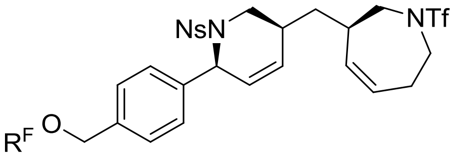

By general method **B1**, the metathesis precursor **38** (1.03 g, 0.86 mmol) and the catalyst **HG-II** (5 mol % then additional 5 mol % portions at 6 and 11 days) gave a crude product that was purified by flash chromatography to give the metathesis product **S14** (0.632 g, 63%), R_f 0.71 (30:70, EtOAc:petrol); δ_H (500 MHz; $CDCl_3$) 7.57 (1H, dd, *J* 8.1, 1.2, nosyl), 7.52 (2H, dd, *J* 8.1, nosyl), 7.33 (1H, dt, *J* 8.1, 1.5, nosyl), 7.20 (2H, d, *J* 8.1, Ar), 7.13 (2H, d, *J* 8.1, Ar), 6.05 (1H, bs,), 5.81 (2H, m,), 5.67 (1H, bs,), 5.52 (1H, s,), 4.72 (2H, s, benzylic-CH_2), 3.78 (1H, d, *J* 13, 2'-CH_2-H_A), 3.61 (1H, dd, *J* 13.0, 3.6, 2'-CH_2-H_B), 3.46 (4H, m,), 2.66 (1H, m,), 2.44 (3H, s,), 2.10 (2H, m, tag), 1.60 (1H, quin, *J* 7.6, 8-CH_2-H_A), 1.51 (1H, quin, *J* 7.6, 8-CH_2-H_B), 1.11 (14H, m, tag), 0.93 (2H, m, tag); δ_C (75 MHz; $CDCl_3$) 147.9, 140.9, 137.4, 134.6, 133.0, 131.5, 130.7, 129.0, 128.0, 127.5, 126.1, 125.4, 124.3, 122.6, 118.3, 64.8, 60.4, 57.5, 53.2, 49.4, 44.7, 32.3, 30.2, 25.6, 17.7, 17.6, 12.6, 0.1; ν_{max}/cm^{-1} (film) 3028, 2945, 2869, 1547, 1385; m/z (ES) [$M+NH_4$] 1193.3 ($M+NH_4$, 100%); HRMS Found: 1198.2076, $C_{42}H_{45}F_{20}N_3Na_1O_7S_2Si_1$ requires 1198.2041.

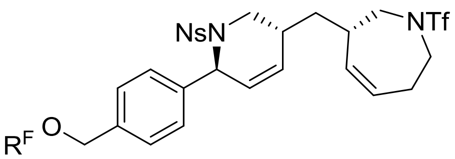

Also obtained was (*S*)-3-(((3*R*,6*S*)-6-(4-(((3,3,4,4,5,5,6,6,7,7,8,8,9,9,10,10,10-heptadecafluorodecyl) diiso propylsilyloxy)methyl)phenyl)-1-(2-nitrophenylsulfonyl)-1,2,3,6-tetrahydropyridin-3-yl)methyl)-1-(trifluoromethylsulfonyl)-2,3,6,7-tetrahydro-1*H*-azepine **S15** (0.298, 29%), R_f 0.63 (30:70, EtOAc:petrol); δ_H (500 MHz; $CDCl_3$) 7.90 (1H, d, *J* 7.5, nosyl), 7.66 (1H, dt, *J* 8.1, 1.2, nosyl), 7.59 (2H, m, nosyl), 7.38 (2H, d, *J* 8.1, Ar), 7.30 (2H, d, *J* 8.1, Ar), 5.95 (1H, ddd, *J* 10.3, 4.2, 2.5), 5.85 (2H, d, *J* 10.3), 5.68 (1H, bs,), 5.64 (1H, s,), 4.81 (2H, s, benzyl-CH_2), 3.93 (1H, ddd, *J* 14.1, 6.4), 3.53 (4H, m,), 2.85 (1H, dd, *J* 14.1, 10.9), 2.58 (1H, s,), 2.43 (2H, m,), 2.31 (1H, m,), 2.15 (2H, m, tag), 1.43 (2H, m,), 1.12 (14H, m, tag), 0.95 (2H, m, tag); δ_C (75 MHz; $CDCl_3$) 148.1, 140.9, 137.7, 134.2, 133.5, 132.9, 131.7, 131.4, 130.5, 129.5, 128.9, 128.3, 128.0, 126.7, 126.4, 126.2, 126.0, 124.2, 122.5, 118.5, 118.2, 115.4, 111.4, 110.9, 64.9, 56.9, 53.1, 49.6, 44.3, 35.8, 31.1, 30.1, 25.5, 17.7, 17.6, 12.5, 0.1; ν_{max}/cm^{-1} (film) 3028, 2945, 2869, 1547, 1385; m/z (ES) [M+NH_4] 1193.3 (100%, M+NH_4); HRMS Found: 1193.2545, $C_{42}H_{49}F_{20}N_4O_7S_2Si_1$ requires 1193.2487.

(*R*)-3-(((3*S*,6*S*)-6-(4-(((3,3,4,4,5,5,6,6,7,7,8,8,9,9,10,10,10-Heptadecafluorodecyl)diisopropylsilyloxy)-methyl)phenyl)-1,2,3,6-tetrahydropyridin-3-yl)methyl)-1-(trifluoromethylsulfonyl)-2,3,6,7-tetrahydro-1*H*-azepine (51)

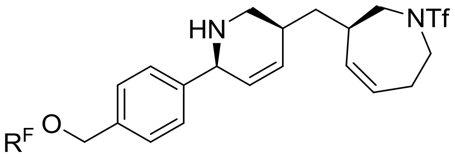

By general procedure **C1**, the metatheis product **S14** (0.700 g, 0.60 mmol) gave a crude product, which was purified by F–SPE and flash column chromatography to furnish the amine **51** (470 mg, 85%), R_f 0.30 (30:70, EtOAc:petrol); δ_H (500 MHz; $CDCl_3$) 7.36 (2H, d, *J* 8.3, Ar), 7.32 (2H, d, *J* 8.3, Ar), 5.86 (1H, quin, *J* 5.7, 5'-H), 5.82 (1H, d, *J* 10.9), 5.78 (1H, s,), 5.77 (1H, d, *J* 10.9), 4.82 (2H, s, benzyl-CH_2), 4.46 (1H, s,), 3.69 (4H, m,), 3.28 (1H, dd, *J* 11.4, 5.2, 2'-H_A), 2.66 (2H, dd, *J* 11.4, 8.8, 2'-H_B), 2.45 (3H, bs, 3'-H), 2.14 (2H, m, tag), 1.55 (1H, m, 8-CH_2-H_A), 1.45 (1H, m, 8-CH_2-H_B), 1.11 (14H, m, tag), 0.93 (2H, m, tag); δ_C (75 MHz; $CDCl_3$) 142.7, 140.1, 135.1, 130.5, 128.9, 127.8, 126.3, 65.1, 58.9, 53.7, 49.5, 48.9, 37.3, 32.8, 30.1, 25.5, 17.7, 17.6, 12.6, 0.1; ν_{max}/cm^{-1} (film) 3338, 2945, 2869, 1463, 1386;m/z (ES) [M+H] 991.2 (100%, M+H); HRMS Found: 991.2429, $C_{36}H_{43}F_{20}N_2O_3S_1Si_1$ requires 991.2439.

(*S*)-3-(((3*R*,6*S*)-6-(4-(((3,3,4,4,5,5,6,6,7,7,8,8,9,9,10,10,10-Heptadecafluorodecyl)diisopropylsilyloxy)-methyl)phenyl)-1,2,3,6-tetrahydropyridin-3-yl)methyl)-1-(trifluoromethylsulfonyl)-2,3,6,7-tetrahydro-1*H*-azepine (52)

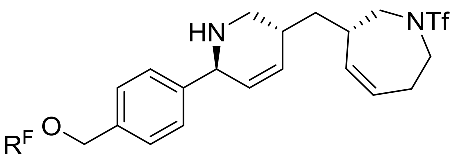

By general procedure **C1**, the metatheis product **S15** (0.200 g, 0.17 mmol) gave a crude product which was purified by F–SPE and flash column chromatography to furnish the amine **52** (158 mg, 94%), R_f 0.38 (70:30, EtOAc:petrol); δ_H (500 MHz; $CDCl_3$) 7.32 (2H, d, *J* 8.3, Ar), 7.29 (2H, d, *J* 8.3, Ar), 5.88 (1H, dt, *J* 10.2, 2.1), 5.81 (1H, m,), 5.76 (1H, dt, *J* 10.2, 2.1), 5.72 (1H, dd, *J* 11.1, 3.4), 4.78 (2H, s, benzyl-CH_2), 4.44 (1H, d, *J* 2.3, 6'-H), 3.69 (4H, m,), 3.04 (1H, dd, *J* 12.1, 4.7), 2.75 (1H, dd , *J* 12.1, 4.7), 2.63 (1H, m,), 2.42 (2H, m,), 2.26 (1H, m,), 2.10 (2H, m, tag), 1.63 (2H, m, 8-CH_2), 1.07 (14H, m, tag), 0.90 (2H, m, tag); δ_C (75 MHz; $CDCl_3$) 142.5, 140.0, 130.1, 128.8, 127.9, 126.3, 65.1, 58.1, 53.6, 49.4, 45.9, 37.4, 32.2, 30.1, 25.5, 17.7, 17.6, 12.6, 0.1; ν_{max}/cm^{-1} (film) 3353, 2495, 2869, 1651, 1463, 1385; m/z (ES) [M+H] 991.3 (100%, M+H); HRMS Found: 991.2429, $C_{36}H_{43}F_{20}N_2O_3S_1Si_1$ requires 991.2439.

(1*R*,9*S*,12*S*,*E*)-9-(4-(((3,3,4,4,5,5,6,6,7,7,8,8,9,9,10,10,10-Heptadecafluorodecyl)diiso-propylsilyloxy)methyl)phenyl)-3,10-diazabicyclo[10.2.1]pentadeca-7,13-dien-4-one (53)

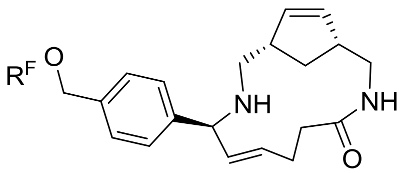

By using general procedure **B1**, the metathesis precursor **25** (1.30 g, 1.01 mmol) and the catalyst HG-II (5 mol % then 3 × 5 mol % portions at 5 day intervals) gave a crude product. By general procedure **C2**, a portion of this crude product (0.375 g) gave a crude product that was purified by F–SPE and flash column chromatography (gradient elution: 2:98 → 10:90, MeOH–$CHCl_3$) to furnish the macrocycle **53** (0.056 g, 8% over two steps), R_f 0.48 (1:9, MeOH:$CHCl_3$); δ_H (500 MHz; $CDCl_3$) 7.30 (2H, d, *J* 8.3, Ar), 7.27 (2H, d, *J* 8.3, Ar), 5.85 (1H, d, *J* 5.1, 12-H), 5.77 (1H, quin, *J* 14.0, 7.7, 5-H), 5.60 (1H, d, *J* 5.1, 11-H), 5.51 (1H, dd, *J* 16.2, 6.8, 6-H), 5.47 (1H, bs, *J* 4.2, NH amine), 4.77 (2H, s, benzyl), 4.20 (1H, d, *J* 6.8, 7-H), 3.60 (1H, dd, *J* 8.1, 2.5, 9-H_A), 3.28 (1H, dt, *J* 13.6, 3.4, 9-H_B), 3.07 (1H, bs, 10-H), 2.84 (1H, bs, 13-H), 2.78 (1H, dd, *J* 11.5, 7.7, 13-H_A), 2.55 (1H, dd, *J* 11.5, 6.8, 13-H_B), 2.46 (1H, m, 4-H_A), 2.38 (1H, m, 3-H_A), 2.31 (1H, m, 4-H_B), 2.21 (2H, q, *J* 12.8, 3-H_B, 15-H_A), 2.09 (2H, m, tag), 1.36 (1H, dt, *J* 18.3, 4.2, 15-H_B), 1.06 (14H, m, tag), 0.88 (2H, m, tag); δ_C (100 MHz; $CDCl_3$) 173.2 (carbonyl), 142.4 (Ar), 139.9 (Ar), 136.7 (C-12), 133.4 (C-5), 132.5 (C-6), 132.2 (C-11), 127.5 (Ar), 126.4 (Ar), 65.3 (benzyl), 64.3 (C-7), 50.9 (C-14), 46.8 (C-13), 45.6 (C-10), 42.5 (C-9), 39.0 (C-4), 29.8 (C-11), 28.2 (C-3), 25.7 (tag), 17.9 (tag), 17.8 (tag), 12.7 (tag), 0.2 (tag); m/z (ES) [M+H] 887.2 (100%, M+H); HRMS Found: 887.2906, $C_{36}H_{44}F_{17}N_2O_2Si_1$ requires 887.2895.

(*R*)-6-(((3,3,4,4,5,5,6,6,7,7,8,8,9,9,10,10,10-Heptadecafluorodecyl)diisopropylsilyloxy)methyl)-1-(2-nitrophenylsulfonyl)-1'-(trifluoromethylsulfonyl)-1,1',2,2',5,5',6,6'-octahydro-3,3'-bipyridine (S16)

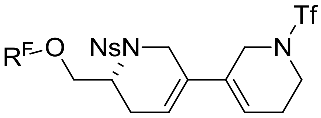

By general method **B2**, the metathesis precursor **40** (0.880 g, 0.80 mmol) and the catalyst HG-II (0.025 g, 5 mol %) gave a crude product which was purified by flash chromatography (gradient elution: 10:90 → 20:80, ethyl acetate–petrol) to afford the cascade product **S16** (0.790 g, 93%), R_f 0.30 (20:80, EtOAc:petrol); δ_H (500 MHz; $CDCl_3$) 8.07 (1H, dd, *J* 6.6, 1.9, nosyl), 7.68 (3H, m, nosyl), 5.86 (1H, t, *J* 4, 4-H), 5.65 (1H, d, *J* 4.7, 4'-H), 4.22 (1H, d, *J* 17.1, 2'-H_A), 3.81 (1H, d, *J* 17.1, 2'-H_B), 3.69 (1H, dd, *J* 10.0, 6.6, 7'-CH_2-H_A), 3.62 (1H, dd, *J* 10.0, 6.6, 7'-CH_2-H_B), 3.54 (2H, m, 2-CH_2), 2.53 (1H, d, *J* 17.1, 5'-CH_2-H_A), 2.39 (2H, s, 3-CH_2), 2.27 (1H, dd, *J* 17.1, , 5'-CH_2-H_B), 2.08 (2H, m, tag), 1.00 (14H, s, tag), 0.79 (2H, m, tag); δ_C (75 MHz; $CDCl_3$) 148.2 (nosyl), 134.0 (nosyl), 133.9 (nosyl), 132.2 (nosyl), 131.3 (nosyl), 130.1 (C-5), 129.6 (C-3'), 124.9 (nosyl), 122.5 (nosyl), 120.6 (C-4), 119.4 (C-4'), 62.5 (C-7'), 52.0 (C-6'), 45.3 (C-6), 43.3 (C-2), 41.3 (C-2'), 25.6 (C-3), 25.5 (tag), 25.4 (C-5'), 17.7 (tag), 17.6 (tag), 12.5 (tag), 0.1 (tag); ν_{max}/cm^{-1} (film) 2946, 2870, 1539, 1463, 1386; m/z (ES) [M+NH_4] 1089.2 (100%, M+NH_4); HRMS Found: 1089.1848, $C_{34}H_{41}F_{20}N_4O_7S_2Si_1$ requires 1089.1861.

(*R*)-6'-(((3,3,4,4,5,5,6,6,7,7,8,8,9,9,10,10,10-Heptadecafluorodecyl)diisopropylsilyloxy)methyl)-1-(trifluoromethylsulfonyl)-1,1',2,2',5,5',6,6'-octahydro-3,3'-bipyridine (54)

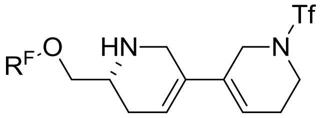

By general method **C1**, the sulfonamide **S16** (0.670 g, 0.63 mmol) gave a crude product which was purified directly by F–SPE to furnish the amine **54** (0.534 g, 96%), R_f 0.49 (60:40, EtOAc:petrol); δ_H (500 MHz; $CDCl_3$) 5.70 (1H, s, 4-H), 5.73 (1H, s, 4'-H), 4.14 (2H, s, 2'-CH_2-H_A, NH), 3.73 (1H, dd, *J* 9.8, 3.4, 7'-CH_2-H_A), 3.56 (6H, m, 2-CH_2, 6-CH_2, 2'-CH_2-H_B), 2.80 (1H, m, 6'-H), 2.36 (2H, s, 3-CH_2), 2.13 (2H, m, tag), 1.99 (2H, m, 5'-CH_2), 1.06 (14H, m, tag), 0.88 (2H, m, tag); δ_C (75 MHz; $CDCl_3$) 133.5 (C-5), 131.3 (C-3'), 121.0 (C-4'), 119.2 (C-4), 67.3 (C-7'), 54.1 (C-6'), 45.5 (C-2'), 45.4 (C-6), 43.4 (C-2), 28.0 (C-3), 25.6 (tag), 25.5 (C-5'), 17.7 (tag), 17.6 (tag), 12.5 (tag), 0.1 (tag); ν_{max}/cm^{-1} (film) 3342, 2945, 2870, 1589, 1461, 1390; m/z (ES) [M+H] 887.2 (100%, M+H); HRMS Found: 887.1854, $C_{28}H_{35}F_{20}N_2O_3S_1Si_1$ requires 887.1813.

(*R*)-6-(6-(((3,3,4,4,5,5,6,6,7,7,8,8,9,9,10,10,10-Heptadecafluorodecyl)diisopropylsilyloxy)methyl)-1-(2-nitrophenylsulfonyl)-1,2,5,6-tetrahydropyridin-3-yl)-1-(2-nitrophenylsulfonyl)-3,4-dihydro-1*H*-azepin-2(7*H*)-one (S17)

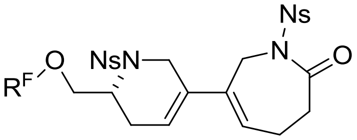

By general method **B2**, the metathesis precursor **27** (0.400 g, 0.35 mmol) and the catalyst HG-II (0.018 g, 5 mol %) gave a crude product which was purified by flash chromatography (gradient elution: 10:90 → 30:70,

ethyl acetate–petrol) to afford the diene **S17** (0.360 g, 93%), R_f 0.32 (40:60, EtOAc:petrol); δ_H (500 MHz; $CDCl_3$) 8.47 (1H, dd, *J* 5.7, 1.9, nosyl), 8.06 (1H, dd, *J* 5.7, 3.4, nosyl), 7.76 (3H, m, nosyl), 7.67 (3H, m, nosyl), 5.99 (1H, d, *J* 4.2, 4'-H), 5.72 (1H, t, *J* 4.2, 5-H), 4.69 (2H, s, 7'-CH_2), 4.21 (1H, d, *J* 7.7, 7'-H_A), 4.23 (1H, s, 2-H), 3.82 (1H, d, *J* 16.7, 7'-H_B), 3.73 (1H, dd, *J* 9.8, 6.6, 2'-CH_2-H_A), 3.66 (1H, dd, *J* 9.8, 6.6, 2'-CH_2-H_B), 2.83 (2H, t, *J* 6.6, 3-CH_2), 2.65 (2H, m, 4-CH_2), 2.54 (1H, d, *J* 16.6, 5'-CH_2-H_A), 2.31 (1H, dd, *J* 19.0, 6.6, 5'-CH_2-H_B), 2.11 (2H, m, tag), 1.02 (14H, m, tag), 0.81 (2H, m, tag); δ_C (75 MHz; $CDCl_3$) 173.7 (carbonyl), 148.1 (nosyl), 135.4 (nosyl), 134.9 (nosyl), 133.9 (C-3'), 133.8 (nosyl), 133.8 (C-6), 133.2 (nosyl), 132.4 (nosyl), 132.3 (nosyl), 131.7 (nosyl), 131.2 (nosyl), 126.0 (C-5), 124.8 (nosyl), 124.7 (nosyl), 121.0 (C-4'), 62.6 (C-2'), 51.9 (C-6'), 44.5 (C-7), 42.1 (C-7'), 35.0 (C-3), 25.6 (C-5'), 25.5 (tag), 25.3 (C-4), 17.8 (tag), 17.7 (tag), 12.5 (tag), 12.4 (tag), 0.1 (tag); ν_{max}/cm^{-1} (film) 3102, 2948, 2869, 1710, 1592, 1539, 1464; m/z (ES) [M+Na] 1170.2 (100%, M+Na); HRMS Found: 1175.1646, $C_{40}H_{41}F_{17}N_4Na_1O_{10}S_2Si_1$ requires 1175.1654.

(*R*)-6-(6-(((3,3,4,4,5,5,6,6,7,7,8,8,9,9,10,10,10-Heptadecafluorodecyl)diisopropylsilyloxy)methyl)-1,2,5,6-tetrahydropyridin-3-yl)-3,4-dihydro-1*H*-azepin-2(7*H*)-one (55)

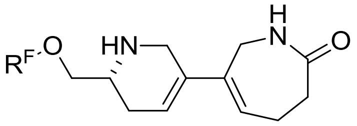

By general method **C2**, the sulfonamide **S17** (0.280 g, 0.24 mmol) gave a crude product which was purified by F–SPE to furnish the amine **55** (0.152 g, 80%), R_f 0.33 (10:90, MeOH:$CHCl_3$); δ_H (500 MHz; $CDCl_3$) 6.35 (1H, t, *J* 5.1, N-H, amide), 5.71 (1H, s, 4'-H), 5.65 (1H, t, *J* 5.1, 5-H), 3.95 (2H, d, *J* 5.1, 7-CH_2), 3.71 (1H, dd, *J* 9.4, 4.2, 7'-CH_2-H_A), 3.58 (1H, dd, *J* 9.4, 7.2, 7'-CH_2-H_B), 3.52 (2H, m, 2'-CH_2), 2.82 (1H, m, 6'-H), 2.67 (2H, td, *J* 7.2, 2.1, 3-CH_2), 2.50 (2H, m, 4-CH_2), 2.12 (2H, m, tag), 2.00 (2H, m, 5'-H), 1.05 (14H, m, tag), 0.87 (2H, m, tag); δ_C (75 MHz; $CDCl_3$) 177.3 (carbonyl), 135.7 (C-6), 136.0 (C-3'), 124.8 (C-5), 120.5 (C-4'), 67.4 (C-7'), 54.0 (C-6'), 46.4 (C-2'), 40.9 (C-7), 33.0 (C-3), 28.2 (C-5), 25.6 (tag), 24.9 (C-4), 17.8 (tag), 17.7 (tag), 12.5 (tag), 0.1 (tag); ν_{max}/cm^{-1} (film) 2945, 2869, 1674, 1463, 1242, 1205; m/s could not be obtained.

(*R*)-2-(((3,3,4,4,5,5,6,6,7,7,8,8,9,9,10,10,10-Heptadecafluorodecyl)diisopropylsilyloxy)methyl)-1-(2-nitrophenylsulfonyl)-5-((1-(trifluoromethylsulfonyl)-1,2,5,6-tetrahydropyridin-3-yl)ethynyl)-1,2,3,6-tetrahydropyridine (S18)

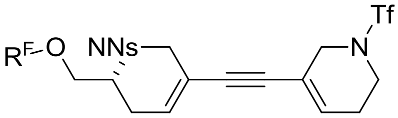

By general method **B2**, the metathesis precursor **42** (0.760 g, 0.68 mmol) and the catalyst HG-II (0.021 g, 5 mol % then 5 mol % portions at 24 hr and 72 hr) gave a crude product which was purified by flash chromatography (gradient elution: 10:90 → 40:60, ethyl acetate–petrol) to afford the cascade product **S18** (0.560 g, 76%), R_f 0.20 (2:8, EtOAc:petrol); δ_H (500 MHz; $CDCl_3$) 8.09 (1H, dd, *J* 6.6, 2.5, nosyl), 7.69 (3H,

m, nosyl), 6.23 (1H, t, *J* 4.2, 4-H), 6.11 (1H, d, *J* 4.2, 4'-H), 4.19 (1H, q, *J* 6.8, 6'-H), 4.00 (2H, s, 6-H), 3.97 (1H, d, *J* 7.4, 2'-CH_2-H_A), 3.74 (1H, d, *J* 14.7, 2'-CH_2-H_B), 3.71 (1H, dd, *J* 10.2, 6.8, 7'-CH_2-H_A), 3.65 (1H, dd, *J* 10.2, 6.8, 7'-CH_2-H_B), 3.56 (2H, s, 2-CH_2), 2.55 (1H, dq, *J* 14.7, 3.2, 5'-CH_2-H_A), 2.37 (2H, s, 3-CH_2), 2.28 (1H, dd, *J* 17.7, 5.3, 5'-CH_2-H_B), 2.09 (2H, m, tag), 1.01 (14H, s, tag), 0.81 (2H, m, tag); δ_C (75 MHz; $CDCl_3$) 148.1 (nosyl), 134.0 (nosyl), 133.7 (nosyl), 132.5 (C-4), 132.3 (nosyl), 131.7 (nosyl), 131.5 (C-4'), 124.9 (nosyl), 118.2 (C-5), 116.8 (C-3'), 87.5 (C-8), 86.6 (C-7), 62.8 (C-7'), 51.7 (C-6'), 47.4 (C-6), 43.6 (C-2'), 43.0 (C-2), 25.8 (C-5'), 25.8 (C-3), 25.6 (tag), 17.8 (tag), 17.7 (tag), 12.5 (tag), 0.1 (tag); ν_{max}/cm^{-1} (film) 2950, 2870, 1592, 1548, 1538, 1463; m/z (ES) [M+NH_4] 1113.2 (100%, M+NH_4); HRMS Found: 1113.1897, $C_{36}H_{41}F_{20}N_4O_7S_2Si_1$ requires 1113.1861.

(*R*)-2-(((3,3,4,4,5,5,6,6,7,7,8,8,9,9,10,10,10-Heptadecafluorodecyl)diisopropylsilyloxy)methyl)-5-((1-(trifluoromethylsulfonyl)-1,2,5,6-tetrahydropyridin-3-yl)ethynyl)-1,2, 3,6-tetrahydropyridine (56)

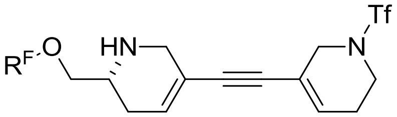

By general method **C1**, the sulfonamide **S18** (0.560 g, 0.51 mmol) gave a crude product that was purified by F–SPE to furnish the amine **56** (0.425 g, 92%), R_f 0.68 (40:60, EtOAc:petrol); δ_H (500 MHz; $CDCl_3$) 6.19 (2H, s, 4, 4'-H), 4.01 (2H, s, 6-CH_2), 3.72 (1H, dd, *J* 9.4, 3.8, 7'-CH_2-H_A), 3.61 (1H, dd, *J* 9.4, 5.9, 7'-CH_2-H_B), 3.56 (2H, m, 2-CH_2), 3.48 (1H, dd, *J* 17.1, 2'-CH_2-H_A), 3.41 (1H, d, *J* 16.2, 2'-CH_2-H_B), 2.83 (1H, m, 6'-H), 2.37 (2H, s, 6-CH_2), 2.12 (2H, m, tag), 2.05 (2H, m, 5'-CH_2), 1.04 (14H, s, tag), 0.88 (2H, m, tag); δ_C (75 MHz; $CDCl_3$) 133.5 (C-4'), 131.4 (C-4), 118.6 (C-3'), 117.8 (C-5), 89.4 (C-8), 85.7 (C-7), 67.1 (C-7'), 53.3 (C-6'), 48.3 (C-2'), 47.5 (C-6), 42.9 (C-2), 28.1 (C-5'), 25.6 (tag), 25.3 (C-3), 17.7 (tag), 17.6 (tag), 12.5 (tag), 0.1 (tag); ν_{max}/cm^{-1} (film) 3422, 2946, 2870, 1647, 1463; m/z (ES) [M+Na] 1066.2 (100%, M+Na); HRMS Found: 1066.1793, $C_{36}H_{37}F_{20}N_3Na_1O_5S_1Si_1$ requires 1066.1796.

(*R*)-6-((6-(((3,3,4,4,5,5,6,6,7,7,8,8,9,9,10,10,10-Heptadecafluorodecyl)diisopropylsilyloxy)methyl)-1-(2-nitrophenylsulfonyl)-1,2,5,6-tetrahydropyridin-3-yl)ethynyl)-1-(2-nitrophenylsulfonyl)-3,4-dihydro-1*H*-azepin-2(7*H*)-one (S19)

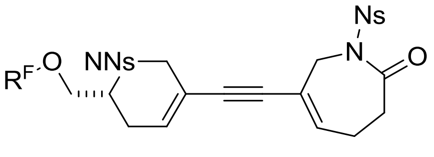

By general method **B2**, the metathesis precursor **43** (0.250 g, 0.20 mmol) and the catalyst HG-II (0.007 g, 5 mol % then 5 mol % after 48 hr) gave a crude product which was purified by flash chromatography (gradient elution: 10:90 → 40:60, ethyl acetate–petrol) to afford the dienyne **S19** (0.131 g, 54%), R_f 0.37 (50:50, EtOAc:petrol); δ_H (500 MHz; $CDCl_3$) 8.47 (1H, m, nosyl), 8.10 (1H, m, nosyl), 7.77 (3H, m, nosyl), 7.69 (3H, m, nosyl), 6.19 (1H, t, *J* 4.2, 5-H), 6.13 (1H, d, *J* 4.2, 4'-H), 4.64 (2H, s, 7-CH_2), 4.23 (1H, q, *J* 6.8, 6'-H), 3.99 (1H, d, *J* 17.1, 2'-CH_2-H_A), 3.78 (1H, d, *J* 17.1, 2'-CH_2-H_B), 3.74 (1H, dd, *J* 10.2, 6.8, 7'-CH_2-H_A), 3.67 (1H, dd, *J* 10.2, 8.1, 7'-CH_2-H_B), 2.82 (2H, t, *J* 6.8, 3-CH_2), 2.61 (2H, m, 4-CH_2), 2.55 (1H, m, 5'-CH_2-

H_A), 2.28 (1H, dd, *J* 17.1, 4.2, 5'-CH_2-H_B), 2.11 (2H, m, tag), 1.03 (14H, s, tag), 0.82 (2H, m, tag); δ_C (75 MHz; $CDCl_3$) 173.4 (carbonyl), 148.2 (nosyl), 148.1 (nosyl), 137.7 (C-5), 135.5 (nosyl), 135.1 (nosyl), 134.0 (nosyl), 133.7 (nosyl), 133.2 (nosyl), 132.4 (nosyl), 131.5 (C-4), 124.9 (nosyl), 124.8 (nosyl), 119.5 (C-6), 116.9 (C-3'), 88.5 (C-8), 87.2 (C-9), 62.9 (C-7'), 51.7 (C-6), 47.0 (C-7), 43.6 (C-2'), 34.8 (C-3), 25.8 (C-4), 25.7 (C-5'), 25.6 (tag), 17.8 (tag), 17.7 (tag), 12.5 (tag), 0.1 (tag); ν_{max}/cm^{-1} (film) 3102, 2946, 2869, 1713, 1592, 1538; m/z (ES) [M+NH_4] 1194.2 (100%, M+NH_4); HRMS Found: 1194.2137, $C_{42}H_{45}F_{17}N_5O_{10}S_2Si_1$ requires 1113.1861.

(*R*)-6-((6-(((3,3,4,4,5,5,6,6,7,7,8,8,9,9,10,10,10-Heptadecafluorodecyl)diisopropylsilyloxy)methyl)-1,2,5,6-tetrahydropyridin-3-yl)ethynyl)-3,4-dihydro-1*H*-azepin-2(7*H*)-one (57)

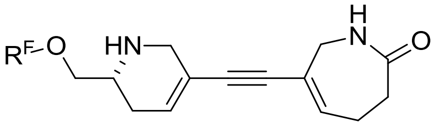

By general procedure **C2**, the sulfonamide **S19** gave a crude product which was purified by F–SPE to furnish the amine **57** (0.68 g, 77%), R_f 0.55 (10:90, MeOH:$CHCl_3$); δ_H (500 MHz; $CDCl_3$) 6.30 (1H, t, *J* 5.7, NH amide), 6.13 (1H, s, 4'-H), 6.11 (1H, t, *J* 4.2, 5-H), 3.88 (2H, d, *J* 5.7, 7-CH_2), 3.71 (1H, dd, *J* 9.9, 4.2, 7'-CH_2-H_A), 3.60 (1H, dd, *J* 9.9, 6.7, 7'-CH_2-H_B), 3.47 (1H, dd, *J* 16.6, 2.6, 2'-CH_2-H_A), 3.40 (1H, d, *J* 16.6, 2'-CH_2-H_B), 2.83 (1H, m, 6'-H), 2.69 (2H, t, *J* 6.7, 3-CH_2), 2.51 (2H, m, 4-CH_2), 2.12 (2H, m, tag), 2.04 (2H, m, 5'-CH_2), 1.03 (14H, m, tag), 0.87 (2H, m, tag); δ_C (75 MHz; $CDCl_3$) 176.7 (carbonyl), 136.9 (C-5), 132.6 (C-4'), 121.2 (C-6), 120.5 (C-3'), 88.2 (C-8), 80.9 (C-9), 67.1 (C-7'), 53.3 (C-6'), 48.3 (C-2'), 44.0 (C-7), 32.6 (C-3), 28.0 (C-5'), 25.6 (tag), 25.3 (C-4), 17.7 (tag), 12.5 (tag), 0,1 (tag); ν_{max}/cm^{-1} (film) 3420, 2947, 2869, 1667, 1463; m/z (ES) [M+H] 807.2 (100%, M+H); HRMS Found: 807.2276, $C_{30}H_{36}F_{17}N_2O_2Si_1$ requires 807.2269.

(2*S*,6*R*)-2-(((3,3,4,4,5,5,6,6,7,7,8,8,9,9,10,10,10-Heptadecafluorodecyl)diisopropylsilyloxy)methyl)-1-(2-nitrophenylsulfonyl)-6-(((*S*)-1-(trifluoromethylsulfonyl)-1,2,5,6-tetrahydropyridin-2-yl)methyl)-1,2,3,6-tetrahydropyridine (S20)

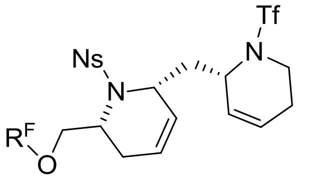

By general method **B2**, the metathesis precursor **30** (0.850 g, 0.76 mmol) and the catalyst HG-II (0.024 g, 5 mol %) gave a crude product which was purified by flash chromatography (gradient elution: 10:90 → 40:60, ethyl acetate–petrol) to afford the diene **S20** (0.430 g, 53%), R_f 0.57 (20:80, EtOAc:petrol); δ_H (500 MHz; $CDCl_3$) 8.02 (1H, dd, *J* 7.3, 1.7, nosyl), 7.66 (3H, m, nosyl), 5.89 (1H, dd, *J* 10.3, 5.5, 4'-H), 5.84 (1H, dt, *J* 10.3, 2.5, 4-H), 5.70 (2H, m, 3', 5-H), 4.25 (3H, m, 6, 6', 2'-H), 4.05 (1H, dd, *J* 14.9, 6.6, 2-CH_2-H_A), 3.69 (3H, m, 7'-CH_2, 2-CH_2-H_B), 2.49 (1H, t, *J* 12.4, 7-CH_2-H_A), 2.38 (2H, m, 5-CH_2), 2.11 (4H, m, tag, 3-CH_2), 1.71 (1H, td, *J* 10.6, 3.4, 7-CH_2-H_B), 1.05 (14H, m, tag), 0.86 (2H, m, tag); δ_C (75 MHz; $CDCl_3$)

148.1 (nosyl), 133.7 (nosyl), 133.4 (nosyl), 132.4 (nosyl), 131.6 (nosyl), 126.7 (C-3'), 125.9 (C-4'), 124.7 (nosyl), 124.2 (C-4), 123.2 (C-5), 64.7 (C-7'), 52.9 (C-6), 51.8 (C-6'), 49.3 (C-2'), 42.3 (C-7), 39.4 (C-2), 25.6 (tag), 23.9 (C-5', C-3), 17.8 (tag), 17.6 (tag), 12.5 (tag), 0.1 (tag); ν_{max}/cm^{-1} (film) 3429, 2947, 2870, 1547, 1384.

(2*R*,6*R*)-2-(((3,3,4,4,5,5,6,6,7,7,8,8,9,9,10,10,10-Heptadecafluorodecyl)diisopropylsilyloxy)methyl)-6-(((*S*)-1-(trifluoromethylsulfonyl)-1,2,5,6-tetrahydropyridin-2-yl)methyl)-1,2,3,6-tetrahydropyridine (58)

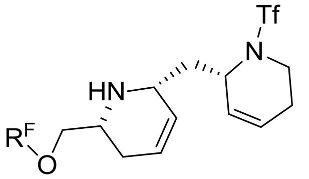

By general method **C1**, the sulfonamide **S20** (0.415 g, 0.38 mmol) gave a crude product which was purified by F–SPE to furnish the amine **58** (0.340 g, 99%), R_f 0.53 (30:70, EtOAc:petrol); δ_H (500 MHz; $CDCl_3$) 5.86 (2H, s, 4, 5-H), 5.81 (1H, ddd, *J* 14.1, 6.4, 3.4, 4'-H), 5.63 (1H, ddd, *J* 9.8, 3'-H), 4.46 (1H, s, 2'-H), 3.91 (1H, ddd, *J* 14.1, 6.4, 2-CH_2-H_A), 3.70 (1H, ddd, *J* 9.8, 4.2, 7'-CH_2-H_A), 3.58 (1H, ddd, *J* 9.4, 6.4, 7'-CH_2-H_B), 3.55 (1H, m), 3.34 (1H, t, *J* 14.1, 2-CH_2-H_B), 2.93 (2H, m, 6'-H), 2.38 (1H, m, 3-CH_2-H_A), 2.13 (2H, m, tag), 2.03 (1H, dt, *J* 18.3, 3.4, 3-CH_2-H_B), 1.89 (2H, m, 5'-CH_2), 1.71 (2H, t, *J* 6.4, 7-CH_2), 1.05 (14H, m, tag), 0.88 (2H, m, tag); ν_{max}/cm^{-1} (film) 3422, 2946, 2870, 1387, 1227; m/z (ES) [M+H] 901.2 (100%, M+H); HRMS Found: 901.2000, $C_{29}H_{37}F_{20}N_2O_3S_1Si_1$ requires 901.1969.

6 Synthesis of Final Products

(*S*)-(4-(4-(1,2,5,6-Tetrahydropyridin-3-yl)-1-(trifluoromethylsulfonyl)-2,5-dihydro-1*H*-pyrrol-2-yl)phenyl)methanol (60a)

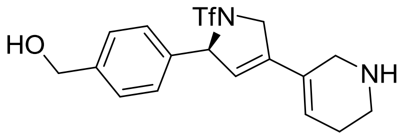

By using general method **D**, the fluorous-tagged silyl ether **45** (0.030g, 0.032 mmol) gave a crude product which was purified by flash chromatography (gradient elution: 2:98 → 15:85, MeOH−$CHCl_3$) to afford the *amine* **60a** (0.006 g, 51%). R_f 0.12 (20:80, MeOH:$CHCl_3$); δ_H (500 MHz; MeOD) 7.36 (2H, d, *J* 6.8, Ar), 7.28 (2H, d, *J* 6.8, Ar), 6.07 (1H, bs, 4-H), 5.86 (2H, s, 4', 5'-H), 4.75 (1H, d, *J* 13, 2'-H_A), 4.71 (1H, d, *J* 13, 2'-H_B), 4.60 (2H, s, benzyl), 3.90 (2H, s, 6-H), 2.56 (2H, bs, 2-H), 1.32 (2H, s, 3-H); δ_C (125 MHz; MeOD) 143.5 (Ar), 139.4 (Ar), 135.4 (C-3'), 128.3 (Ar), 124.4 (Ar), 126.8 (C-4), 126.0 (C-5), 125.5 (C-4'), 73.2 (C-5'), 64.7 (benzyl), 56.5 (C-2'), 41.6 (C-6), 30.5 (C-2), 23.7 (C-3'); m/z (ES) [M+H] 389.1 (100%, M+H); HRMS Found: 389.1141, $C_{17}H_{20}F_3N_2O_3S_1$ requires 389.1141.

(*S*)-(3-(5-(4-(((3,3,4,4,5,5,6,6,7,7,8,8,9,9,10,10,10-Heptadecafluorodecyl)diisopropylsilyloxy)methyl) phenyl)-1-(trifluoromethylsulfonyl)-2,5-dihydro-1*H*-pyrrol-3-yl)-5,6-dihydropyridin-1(2*H*)-yl) (isoxazol-5-yl)methanone (S21)

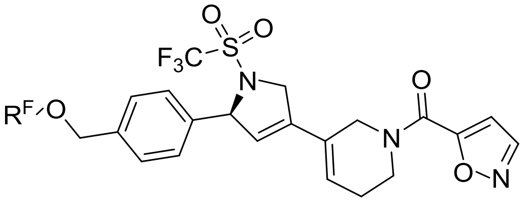

By general method **E1**, the fluorous-tagged amine **45** (0.035 g, 0.037 mmol) gave a crude product which was purified by F–SPE to afford the *amide* **S21** (0.032 g, 81%), R_f 0.42 (40:60, EtOAc:petrol); δ_H (500 MHz; $CDCl_3$) 8.33 (1H, d, *J* 1.6, 5''-H^{maj}), 8.30 (1H, d, *J* 9.3, 5''-H^{min}), 7.31 (2H, d, *J* 8.3, Ar), 7.24 (2H, d, *J* 8.3, Ar^{maj}), 7.22 (2H, d, *J* 8.3, Ar^{min}), 6.84 (1H, s, 6''-H^{min}), 6.81 (1H, d, *J* 1.6, 6''-H^{maj}), 5.94 (1H, s, 4'-H^{min}, 5.90 (1H, s, 4'-H^{maj}), 5.82 (1H, s, 5'-H), 5.77 (1H, s, 4-H^{maj}), 5.62 (1H, s, 4-H^{min}), 4.79 (2H, s, benzyl-*CH_2*), 4.72 (1H, d, *J* 13.5, 2'-H_A), 4.58 (1H, d, *J* 13.5, 2'-H_B), 4.40 (2H, q, *J* 17.1, 6-*CH_2*), 3.84 (2H, m, 2-*CH_2*), 2.50 (2H, s, 3-*CH_2*), 2.1 (2H, m, tag), 1.08 (14H, m, tag), 0.90 (2H, m, tag); δ_C (75 MHz; $CDCl_3$) 163.1 (carbonyl), 157.6 (C-5), 150.6 (C-5''), 142.0 (Ar), 137.6 (Ar), 134.9 (C-3'), 128.0 (Ar), 127.7 (Ar), 127.3 (Ar), 126.5 (Ar), 126.3 (C-4'), 123.6 (C-4^{maj}), 123.1 (C-4^{min}), 108.8 (C-6''^{min}), 108.1 (C-6''^{maj}), 72.0 (C-5'), 65.1 (benzyl), 55.7 (C-2'), 46.1 (C-6^{min}), 43.7 (C-2^{maj}), 43.2 (C-6^{maj}), 39.8 (C-2^{min}), 30.0, 26.6 (C-3), 25.7 (tag), 17.9 (tag), 17.7 (tag), 12.7 (tag), 0.1 (tag); ν_{max}/cm^{-1} (film) 2924, 2869, 1655, 1638, 1462; m/z (ES) [M+Na] 1066.2 (100%, M+Na); HRMS Found: 1066.1793, M+Na requires 1066.1802.

(*S*)-(3-(5-(4-(Hydroxymethyl)phenyl)-1-(trifluoromethylsulfonyl)-2,5-dihydro-1*H*-pyrrol-3-yl)-5,6-dihydropyridin-1(2*H*)-yl)(isoxazol-5-yl)methanone (60b)

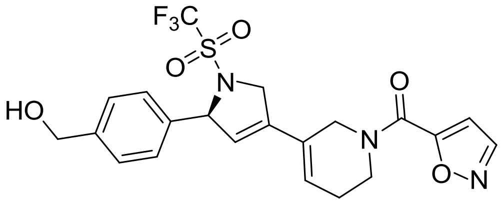

By general method **D**, the fluorous-tagged silyl ether **S21** (0.022 g, 0.024 mmol) gave a crude product which was purified by flash chromatography (gradient elution: 2:98 → 10:90, MeOH–$CHCl_3$) to furnish the alcohol **60b** (0.006 g, 60%). R_f 0.48 (1:9, MeOH:$CHCl_3$); δ_H (500 MHz; CD_3OD) 8.52 (1H, d, *J* 1.9, 5''-H^{maj}), 8.49 (1H, d, *J* 1.9, 5''-H^{min}), 7.36 (2H, d, *J* 8.3, Ar), 7.30 (2H, d, *J* 8.3, Ar), 6.85 (1H, bs, 6''-H), 6.05 (1H, s, 4-H), 5.80 (1H, s, 5'-H), 4.76 (1H, d, *J* 13, 2'-H_A), 4.70 (1H, d, *J* 13, 2'-H_B), 4.60 (2H, s, benzyl), 4.41 (2H, q, *J* 19.1, 6-H), 3.74 (2H, t, *J* 5.5, 2-H), 2.47 (2H, bs, 3-H); δ_C (100 MHz; CD_3OD) 164.4 (carbonyl), 161.0 (C-2''), 152.1 (C-5''), 143.8 (Ar), 140.0 (Ar), 136.4 (C-3'), 129.0, 128.7, 128.6, 127.8, 124.9 (C-4), 108.6 (C-6''), 73.6 (C-5'), 65.2 (benzyl), 57.0 (C-2'), 45.1 (C-6), 44.1 (C-2), 27.6 (C-3'); m/z (ES) [M+Na] 506.1 (100%, M+Na); HRMS Found: 506.0973, $C_{21}H_{20}F_3N_3Na_1O_5S_1$ requires 506.0968.

(*S*)-3-(5-(4-(((3,3,4,4,5,5,6,6,7,7,8,8,9,9,10,10,10-Heptadecafluorodecyl)diisopropylsilyloxy)methyl)-phenyl)-1-(trifluoromethylsulfonyl)-2,5-dihydro-1*H*-pyrrol-3-yl)-*N*-(pyridin-3-yl)-5,6-dihydropyridine-1(2*H*)-carboxamide (S22)

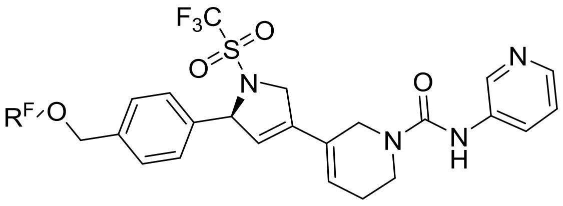

By general method **E2**, the fluorous-tagged amine (0.035 g, 0.037 mmol) gave a crude product which was purified by F–SPE to afford the urea **S22** (0.026 g, 67%), R_f 0.38 (10:90, MeOH:$CHCl_3$); δ_H (500 MHz; $CDCl_3$) 8.43 (1H, s, 4''-pyridyl), 8.26 (1H, d, *J* 4.2, 8''-pyridyl), 7.95 (1H, d, *J* 8.3, 6''-pyridyl), 7.30 (2H, d, *J* 8.3, Ar), 7.24 (2H, d, *J* 8.3, Ar), 7.21 (1H, m, 7''-H pyridyl), 6.62 (1H, s, N-H), 5.93 (1H, t, *J* 4.2, 4-H), 5.80 (1H, s, 4'-H), 5.70 (1H, s, 5'-H), 4.79 (2H, s, benzyl), 4.72 (1H, d, *J* 14, 2'-H_A), 4.57 (1H, dd, *J* 14.0, 4.5, 2'-H_B), 4.20 (2H, s, 6-H), 3.60 (2H, q, *J* 5.7, 2-H), 2.41 (2H, s, 3-H), 2.12 (2H, m, tag), 1.08 (14H, m, tag), 0.90 (2H, m, tag); δ_C (75 MHz; $CDCl_3$) 154.0 (carbonyl), 144.6 (C-4'), 141.9 (C-4), 141.6 (pyridyl), 137.7 (Ar), 136.0 (Ar), 135.0 (pyridyl), 128.2 (pyridyl), 127.9 (pyridyl), 127.7 (Ar), 126.5 (Ar), 123.9, 122.9, 72.0 (C-5'), 65.0 (benzyl), 55.8 (C-2'), 43.9 (C-6), 41.0 (C-2), 25.8 (C-3), 25.7 (tag), 17.8 (tag), 17.7 (tag), 12.7 (tag), 0.1 (tag); m/z (ES) [M+H] 1069.2 (100%, M+H); HRMS Found: 1069.2213, $C_{39}H_{41}F_{20}N_4O_4S_1Si_1$ requires 1069.2293.

(*S*)-3-(5-(4-(Hydroxymethyl)phenyl)-1-(trifluoromethylsulfonyl)-2,5-dihydro-1*H*-pyrrol-3-yl)-*N*-(pyridin-3-yl)-5,6-dihydropyridine-1(2*H*)-carboxamide (60c)

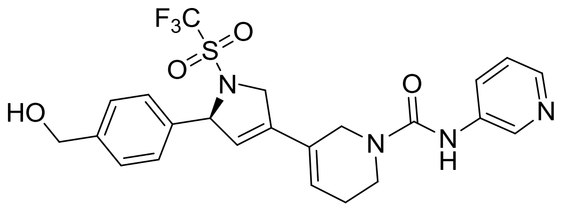

By general method **D**, the fluorous-tagged silyl ether **S22** (0.026 g, 0.024 mmol) gave a crude product which was purified by flash chromatography (gradient elution: 2:98 → 10:90, MeOH–$CHCl_3$) to give the *alcohol* **60c** (0.013 g, 98%), R_f 0.28 (10:90, MeOH:$CHCl_3$); δ_H (500 MHz; CD_3OD) 8.59 (1H, s, 8''-H pyridyl), 8.18 (1H, s, 4''-H pyridyl), 7.91 (1H, dq, *J* 8.3, 1.2, 6''-H pyridyl), 7.36 (2H, d, *J* 8.3, Ar), 7.34 (1H, m, 7''-H pyridyl), 7.30 (2H, d, *J* 8.3, Ar), 6.06 (1H, septate, *J* 6.4, 4.2, 2.1, 4-H), 5.86 (2H, bs, 4', 5'-H), 4.75 (1H, d, *J* 12.5, 2'-H_A), 4.69 (1H, d, *J* 12.5, 2'-H_B), 4.60 (2H, s, benzyl), 4.25 (2H, q, *J* 2.1, 6-H), 3.66 (2H, m, 2-H), 2.41 (2H, s, 3-H); δ_C (75 MHz; MeOD–CD_3OD) 157.2 (carbonyl), 143.8 (Ar), 143.3 (pyridyl), 142.6 (pyridyl), 136.2 (pyridyl), 130.0 (pyridyl), 129.2 (pyridyl), 128.4 (Ar), 128.2 (Ar), 127.9 (C-4'), 124.0 (C-4), 73.2 (C-5'), 64.7 (benzyl), 56.7 (C-2'), 44.9 (C-6), 41.4 (C-2), 26.4 (C-3); m/z (ES) [M+H] 509.1 (100%, M+H); HRMS Found: 509.1459, $C_{23}H_{24}F_3N_4O_4S_1$ requires 509.1465.

(*S*)-(3-(5-(4-(((3,3,4,4,5,5,6,6,7,7,8,8,9,9,10,10,10-Heptadecafluorodecyl)diisopropylsilyloxy)methyl) phenyl)-1-(trifluoromethylsulfonyl)-2,5-dihydro-1*H*-pyrrol-3-yl)-5,6-dihydropyridin-1(2*H*)-yl) (morpholino)methanone (S23)

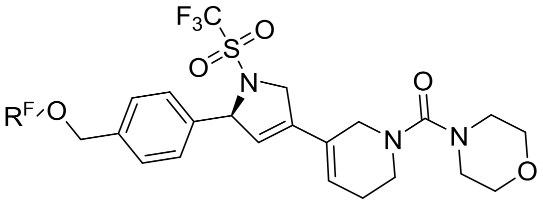

By general method **E3**, the fluorous-tagged amine (0.035 g, 0.037 mmol) gave a crude product which was purified by F–SPE to afford the *urea* **S23** (0.036 g, 94%), R_f 0.29 (40:60, EtOAc:petrol); δ_H (500 MHz; $CDCl_3$) 7.30 (2H, d, *J* 8.1, Ar), 7.24 (2H, d, *J* 8.1, Ar), 5.85 (1H, s, 4-H), 5.79 (1H, s, 5'-H), 5.65 (1H, s, 4'-H), 4.79 (2H, s, benzyl-*CH_2*), 4.70 (1H, d, *J* 13.2, 2'-H_A), 4.55 (1H, d, *J* 13.2, 2'-H_B), 3.94 (2H, s, 6-*CH_2*), 3.67 (4H, t, *J* 4.7, 6'',4''-*CH_2*), 3.41-3.30 (2H, m, 2-*CH_2*), 3.27 (4H, t, J 4.7, 3'', 7''-*CH_2*), 2.36 (2H, s, 3-*CH_2*), 2.11 (2H, m, tag), 1.09 (14H, m, tag), 0.90 (2H, m, tag); δ_C (75 MHz; $CDCl_3$) 164.1 (carbonyl), 141.9 (Ar), 137.8 (Ar), 135.4 (C-3'), 128.9 (C-5), 127.7 (Ar), 126.8 (C-4), 126.5 (Ar), 122.5 (C-4'), 71.9 (C-5), 66.9 (6'',4''), 65.0 (benzyl), 55.8 (C-2'), 47.6 (3'',7''), 46.3 (6-C), 44.2 (C-4), 25.8 (C-3'), 25.7 (tag), 17.8 (tag), 17.7 (tag), 12.7 (tag), 0.1 (tag); ν_{max}/cm^{-1} (film) 2948, 2868, 1643, 1463, 1422; m/z (ES) [M+H] 1062.2 (100%, M+H); HRMS Found: 1062.2449, $C_{38}H_{44}F_{20}N_3O_5S_1Si_1$ requires 1062.2446.

(*S*)-(3-(5-(4-(Hydroxymethyl)phenyl)-1-(trifluoromethylsulfonyl)-2,5-dihydro-1*H*-pyrrol-3-yl)-5,6-dihydropyridin-1(2*H*)-yl)(morpholino)methanone (60d)

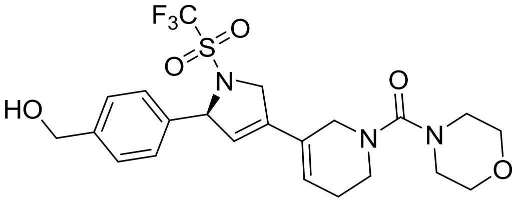

By general method **D**, the fluorous-tagged silyl ether **S23** (0.037 g, 0.035 mmol) gave a crude product which was purified by flash chromatography (gradient elution: 2:98 → 10:90, MeOH–$CHCl_3$) to furnish the alcohol **60d** (0.014 g, 81%), R_f 0.29 (10:90, MeOH:$CHCl_3$); δ_H (500 MHz; CD_3OD) 7.35 (2H, d, *J* 8.3, Ar), 7.27 (2H, d, *J* 8.3, Ar), 5.98 (1H, t, *J* 4.9, 4-H), 5.78 (1H, s, 4'-H), 4.71 (2H, d, *J* 13.4, 2'-H_A), 4.66 (2H, d, *J* 13.4, 2'-H_B), 4.60 (2H, s, benzyl-*CH₂*), 3.98 (2H, td, *J* 19.0, 1.7, 6-*CH₂*), 3.64 (4H, t, *J* 4.9, 4'', 6''-*CH₂*), 3.39 (2H, m, 2-*CH₂*), 3.26 (4H, t, *J* 4.9, 3'', 7''-*CH₂*), 2.37 (2H, s, 3-*CH₂*); δ_C (75 MHz; CD_3OD) 165.5 (carbonyl), 143.3 (Ar), 139.8 (Ar), 136.2 (C-3'), 129.5 (C-5), 128.4 (Ar), 128.2 (Ar), 127.9 (C-4), 123.7 (C-4'), 73.2 (C-5'), 67.6 (C-4'', 6''), 64.8 (benzyl), 56.6 (C-2'), 47.3 (C-6), 44.5 (C-2'), 26.2 (C-3), 48.4 (C-3'',7''); ν_{max}/cm^{-1} (film) 3404, 2922, 2854, 2114, 1614 1427; m/z (ES) [M+H] 502.2 (100%, M+H); HRMS Found: 502.1626, $C_{22}H_{27}F_3N_3O_5S_1$ requires 502.1618.

(3-((1*S*,4*S*,5*R*)-4-(4-(((3,3,4,4,5,5,6,6,7,7,8,8,9,9,10,10,10-Heptadecafluorodecyl)diisopropylsilyloxy) methyl)phenyl)-3-(trifluoromethylsulfonyl)-3-azabicyclo [3.1.0]hexan-1-yl)-5,6-dihydropyridin-1(2*H*)-yl)(isoxazol-5-yl)methanone (S24)

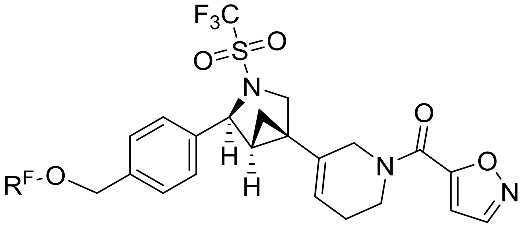

By general method **E1**, the fluorous-tagged amine **46** (0.060 g, 0.07 mmol), Et_3N (30 μL, 0.30 mmol) gave a crude product which was purified by flash chromatography (gradient elution: 10:90 → 30:70, ethyl acetate–petrol) to afford the amide **S24** (0.025 g, 39%), R_f 0.59 (40:60, EtOAc:petrol); δ_H (500 MHz; $CDCl_3$) 8.53 (1H, d, *J* 4.9, 5''-H^{maj}), 8.33 (1H, d, *J* 1.5, 5''-H^{maj}), 7.37 (2H, d, *J* 8.3, Ar^{min}), 7.33 (2H, d, *J* 8.3, Ar^{min}), 7.31 (4H, m, Ar^{maj}), 6.83 (1H, s, 6''^{min}), 6.80 (1H, d, *J* 1.5, 6''^{maj}), 5.82 (1H, m, 4-H), 5.36 (1H, d, *J* 4.5, 6'-H^{min}), 5.23 (1H, d, *J* 3.6, 6'-H^{maj}), 4.81 (2H, s benzyl-CH_2^{min}), 4.80 (2H, s, benzyl-CH_2^{maj}), 4.19 (1H, d, *J* 10.1, 2'-H_A^{min}), 4.14 (1H, d, *J* 10.1, 2'-H_B^{min}), 4.11 (2H, s, 6-H^{maj}), 3.97 (1H, d, *J* 10.1, 2'-H_A^{maj}), 3.93 (1H, d, *J* 10.1, 2'-H_B^{maj}), 3.88 (2H, s, 6-H^{min}), 3.80 (1H, dd, *J* 10.0, 5.5, 2-H_A^{maj}), 3.76 (1H, dd, *J* 10.1, 5.5, 2-H_B^{maj}), 3.70 (2H, q, *J* 6.6, 2-H_B^{min}), 2.34 (2H, bs, 3-*CH₂*), 2.29 (1H, quin, *J* 6.6, 5'-H^{maj}), 2.10 (4H, m, tag^{min}), 2.01 (1H, quin, *J* 4.2, 5'-H^{min}), 1.60 (1H, q, *J* 5.1, 4'-H_A), 1.30 (1H, t, *J* 5.1, 4'-H_B), 1.11 (14H, m, tag), 0.98 (1H, t, *J* 6.4, 4'-H_B), 0.90 (2H, m, tag); δ_C (75 MHz; $CDCl_3$) 163.8 (carbonyl), 150.3 (C-5''^{maj}), 148.8 (C-5''^{min}), 141.4 (Ar^{min}), 141.3 (Ar^{maj}), 136.2 (Ar^{maj}), 136.0 (Ar^{min}), 134.7 (C-5), 132.4, 127.3, 127.2, 126.0, 125.9 (Ar), 123.6 (C-4^{min}), 121.8 (C-4^{maj}), 108.2 (C''-6^{min}), 107.7 (C-6''^{maj}), 66.2 (C-6'^{min}), 66.0 (C-6'^{maj}), 64.9 (benzyl), 57.4 (C-6^{min}), 56.2 (C-6^{maj}), 46.5 (C-2'^{min}), 43.7 (C-2'^{maj}), 43.6 (C-2), 33.0 (C-5'), 31.0 (C-5^{min}), 25.9 (C-3^{maj}), 25.8 (tag), 25.6 (C-3^{min}), 17.6 (tag), 17.5 (tag), 15.8 (C-4'^{min}), 13.8 (C-4'^{maj}), 12.5 (tag), 0.1 (C-

tag); ν_{max}/cm^{-1} (film) 2948, 2875, 1644, 1427, 1393; m/z (ES) [M+NH_4] 1075.2 (100%, M+NH_4); HRMS Found: 1058.2167, $C_{38}H_{40}F_{20}N_3O_5S_1Si_1$ requires 1058.2133.

(3-((1*S*,4*S*,5*R*)-4-(4-(Hydroxymethyl)phenyl)-3-(trifluoromethylsulfonyl)-3-azabicyclo[3.1.0]hexan-1-yl)-5,6-dihydropyridin-1(2*H*)-yl)(isoxazol-5-yl)methanone (61b)

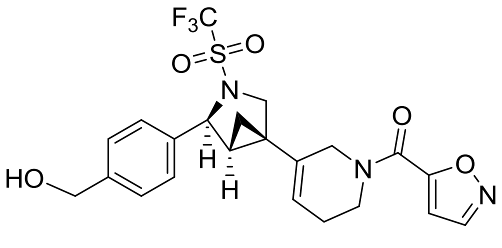

By general method **D**, the fluorous-tagged silyl ether **S24** (0.022 g, 0.020 mmol) gave a crude product which was purified by flash chromatography (gradient elution: 2:98 → 10:90, MeOH–$CHCl_3$) to furnish the alcohol **61b** (0.007 g, 70%) R_f 0.29 (80:20, EtOAc: petrol); δ_H (500 MHz; CD_3OD) 8.47 (1H, bs, 5''-H^{min}), 8.45 (1H, d, *J* 1.7, 5''-H^{maj}), 7.28 (2H, d, *J* 8.1, Ar), 7.27 (2H, d, *J* 8.1, Ar), 6.82 (1H, bs, 6''-H^{min}), 6.78 (1H, d, *J* 1.7, 6''-H^{maj}), 5.85 (1H, d, *J* 1.7, 4-H), 5.30 (1H, d, *J* 3.6, 6'-H^{maj}), 5.25 (1H, d, *J* 3.6, 6'-H^{min}), 4.54 (2H, s, benzyl-CH_2^{maj}), 4.53 (2H, s, benzyl-CH_2^{min}), 4.11 (2H, quin, *J* 17.3, 1.7), 3.93 (1H, d, *J* 9.6, 2'-H_A^{maj}), 3.90 (1H, d, *J* 9.6, 2'-H_B^{maj}), 3.82 (2H, s, 2'-H^{min}), 3.61 (2H, q, *J* 9.5, 5.7, 2-H), 2.25 (2H, m, 3-H), 2.15 (1H, quin, *J* 4.2, 5'-H^{maj}), 2.06 (1H, quin, J 4.2, 5'-H^{min}), 1.15 (1H, t, *J* 4.6, 4'-H_A^{min}), 0.93 (1H, t, *J* 4.6, 4'-H_A^{maj}), 0.84 (2H, m, 4'-$H_B^{min\&maj}$); δ_C (125 MHz; CD_3OD) 164.1 (carbonyl), 159.5 (C-5), 151.6 (C-5''), 142.7 (Ar), 138.5 (Ar), 133.8, 128.3 (Ar), 127.8 (Ar), 124.9 (C-4^{min}), 124.1 (C-4^{maj}), 122.9, 120.4, 108.0 (C-6''^{min}), 107.5 (C-6''^{maj}), 67.4 (C-6'), 64.9 (benzyl), 57.5 (C-2'), 48.0 (C-2), 44.8 (C-6^{min}), 44.5 (C-6^{maj}), 32.4 (C-4'), 31.9 (C-3'), 26.7 (C-3^{maj}), 25.3 (C-3), 14.7 (C-4'); m/z (ES) [M+H] 498.1 (100%, M+H); HRMS Found: 498.1311, $C_{22}H_{23}F_3N_3O_5S_1$ requires 498.1305.

(3-((1*R*,4*S*,5*S*)-4-(4-(((3,3,4,4,5,5,6,6,7,7,8,8,9,9,10,10,10-Heptadecafluorodecyl)diisopropylsilyloxy) methyl)phenyl)-3-(trifluoromethylsulfonyl)-3-azabicyclo [3.1.0]hexan-1-yl)-5,6-dihydropyridin-1(2*H*)-yl)(isoxazol-5-yl)methanone (S25)

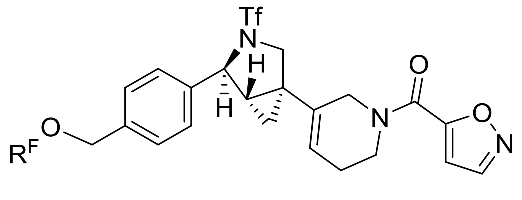

By general method **E1**, the fluorous-tagged amine **47** (0.030 g, 0.03 mmol) gave a crude product which was purified by flash chromatography (gradient elution: 10:90 → 30:70, ethyl acetate–petrol) to afford the amide **S25** (0.014 g, 43%), R_f 0.31 (30:70, EtOAc:petrol); δ_H (500 MHz; $CDCl_3$) 8.33 (1H, s, 5''-H), 7.31 (2H, d, *J* 8.1, Ar), 7.22 (2H, d, *J* 8.1, Ar), 6.80 (1H, d, *J* 3.4, 6''-H), 5.73 (1H, s, 6'-H^{maj}), 5.70 (1H, s, 6'-H^{min}), 5.58 (1H, d, *J* 1.3, 4-H^{maj}), 5.54 (1H, d, *J* 1.3, 4-H^{min}), 5.44 (1H, s), 4.79 (2H, s, benzyl), 4.43 (1H, d, *J* 13.2, 2'-H_A^{maj}), 4.39 (1H, d, *J* 14.1, 2'-H_A^{min}), 4.35 (1H, d, *J* 13.2, 2'-H_B^{maj}), 4.33 (1H, d, *J* 14.1, 2'-H_B^{min}), 4.26 (1H, m, 6-H_A^{maj}), 4.21 (1H, d, *J* 13.4, 6-H_A^{min}), 4.17 (1H, d, *J* 13.4, 6-H_B^{min}), 4.05 (1H, m), 3.89 (1H, d, *J* 13.1, 6-

$H_B{}^{maj}$), 3.82 (2H, m), 3.74 (1H, d, *J* 13.6), 3.65 (1H, d, *J* 13.6), 3.34 (1H, m, 2-H_A), 3.09 (1H, m, 2-H_B), 2.19 (2H, m, 3-H), 2.10 (2H, m, tag), 1.99 (2H, m, 5'-H), 1.39 (1H, q, *J* 8.9, 4'-H_A), 1.06 (14H, m, tag), 0.90 (2H, m, tag), 0.81 (1H, t, *J* 5.7, 4'-H_B); δ_C (100 MHz; $CDCl_3$) 163.9, 159.6, 157.8, 150.6, 141.9, 127.6, 126.5, 123.7, 108.3, 76.6, 71.9, 71.8, 71.7, 65.1, 56.0, 55.9, 44.7, 43.9, 41.2, 30.0, 25.7, 24.0, 17.8, 17.7, 12.7, 0.2; ν_{max}/cm^{-1} (film) 3434, 2956, 1644, 1428, 1389.

(3-((1*R*,4*S*,5*S*)-4-(4-(Hydroxymethyl)phenyl)-3-(trifluoromethylsulfonyl)-3-azabicyclo[3.1.0]hexan-1-yl)-5,6-dihydropyridin-1(2*H*)-yl)(isoxazol-5-yl)methanone (62b)

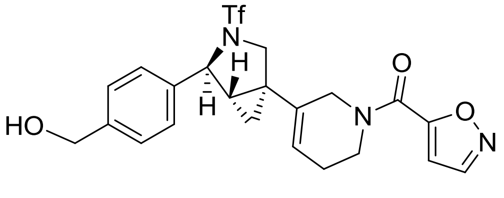

By general method **D**, the fluorous-tagged silyl ether **S25** (0.014 g, 0.013 mmol) gave a crude product which was purified by flash chromatography (gradient elution: 20:80 → 50:50, ethyl acetate–petrol) to furnish the alcohol **62b** (0.004 g, 63%), R_f 0.32 (70:30, EtOAc:petrol); δ_H (500 MHz; CD_3OD) 8.50 (1H, d, *J* 1.8, 5''-H^{maj}), 8.49 (1H, bs, 5''-H^{min}), 7.35 (2H, d, *J* 7.2, Ar^{maj}), 7.32 (2H, d, *J* 7.2, Ar^{maj}), 7.31 (4H, m, Ar), 6.85 (1H, d, *J* 1.8, 6''-H^{maj}), 6.78 (1H, d, *J* 1.8, 6''-H^{min}), 5.75 (1H, s, 6'-H^{maj}), 5.71 (1H, s, 6'-H^{min}), 5.66 (1H, d, *J* 2.1, 4-H^{maj}), 5.52 (1H, d, *J* 2.1, 4-H^{min}), 4.60 (2H, s, $benzyl^{maj}$), 4.59 (2H, s, $benzyl^{min}$), 4.45 (2H, m, 2'-H^{maj}), 4.20 (1H, d, *J* 13.3, 2'-$H_A{}^{min}$), 4.12 (1H, d, *J* 13.3, 2'-$H_B{}^{min}$), 4.04-4.86 (2H, m, 6-H), 3.65 (1H, m, 2-$H_A{}^{min}$), 3.43 (1H, m, 2-$H_B{}^{min}$), 2.22 (2H, m, 2-H^{maj}), 1.89 (2H, m, 3-H), 1.50 (2H, m, 5'-$H^{maj\ \&\ min}$), 1.19 (2H, m, 4'-$H_A{}^{maj\ \&\ min}$), 0.78 (2H, q, *J* 5.5, 4'-H_B); δ_C (75 MHz; CD_3OD) 164.0 (C-1''^{maj}), 163.9 (C-1''^{min}), 159.7 (C-5^{maj}), 159.6 (C-5^{min}), 151.7 (C-5''^{min}), 151.6 (C-5''^{maj}), 143.6 (Ar), 142.1 (Ar), 142.0 (Ar), 141.6 (Ar), 141.5 (C-5), 128.4 (Ar), 128.3 (Ar), 128.2 (Ar), 124.9 (C-4^{min}), 124.7 (C-4^{maj}), 107.9 (C-6''^{maj}), 107.8 (C-6''^{min}), 72.9 (C-6'^{maj}), 72.8 (C-6'^{min}), 64.8 (benzyl), 56.8 (C-2'^{maj}), 45.4 (C-2'^{min}), 45.3 (C-2^{min}), 44.8 (C-2^{maj}), 41.9, 24.7 (C-3), 22.9 (C-3), 21.1 (C-5'), 20.8 (C-5'), 17.9 (C-4'), 17.5 (C-4'); m/z (ES) [M+NH_4] 515.2 (100% M+NH_4); HRMS Found: 520.1137, $C_{22}H_{22}F_3Na_1O_5S_1$ requires 520.1124.

(*S*)-(4-(4-((1,2,5,6-Tetrahydropyridin-3-yl)ethynyl)-1-(trifluoromethylsulfonyl)-2,5-dihydro-1*H*-pyrrol-2-yl)phenyl)methanol (63a)

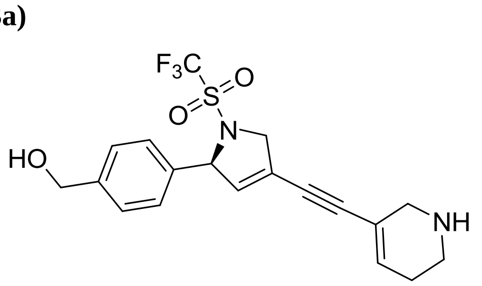

By general method **D**, the fluorous-tagged silyl ether **49** (0.020 g, 0.020 mmol) gave a crude product which was purified by flash chromatography (gradient elution: 2:98 → 10:90, MeOH–$CHCl_3$) to give the alcohol **63a** (0.007 g, 83%) m.p. 67-69 °C; R_f 0.40 (10:90, MeOH:$CHCl_3$); δ_H (500 MHz; CD_3OD) 7.36 (2H, d, *J* 8.3, Ar), 7.29 (2H, d, *J* 8.3, Ar), 6.32 (1H, septet, *J* 6.0, 4.3, 1.7, 4-H), 6.04 (1H, q, *J* 2.1, 4'-H), 5.84 (2H, d, *J*

2.1, 5’-H), 4.60 (2H, s, benzyl), 4.51 (2H, s, 2’-H), 3.36 (2H, q, *J* 4.2, 1.9, 6-H), 2.91 (2H, t, *J* 5.5, 2-H), 2.23 (2H, m, 3-H); δ_C (75 MHz; CD_3OD) 143.5 (Ar), 136.2 (C-4), 135.1 (C-4’), 128.5 (Ar), 128.3 (Ar), 120.8 (C-3’), 120.4 (C-5), 93.8 (C-8), 81.4 (C-7), 72.7 (C-5’), 64.7 (benzyl), 58.4 (C-2), 47.6 (C-6), 42.1 (C-2), 26.0 (C-3’); m/z (ES) [M+H] 413.1 (100%, M+H); HRMS Found: 413.1124, $C_{19}H_{20}F_3N_2O_3S_1$ requires 413.1141.

(*S*)-3-((5-(4-(((3,3,4,4,5,5,6,6,7,7,8,8,9,9,10,10,10-Heptadecafluorodecyl)diisopropylsilyloxy)methyl) phenyl)-1-(trifluoromethylsulfonyl)-2,5-dihydro-1*H*-pyrrol-3-yl)ethynyl)-*N*-(pyridin-3-yl)-5,6-dihydro-pyridine-1(2*H*)-carboxamide (S27)

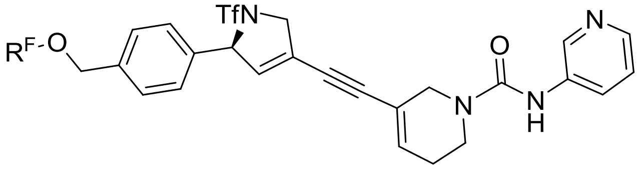

By using general procedure **E2**, the fluorous-tagged amine **49** (0.035 g, 0.036 mmol) gave a crude product which was purified by F–SPE to afford the urea **S27** (0.032 g, 83%), R_f 0.37 (10:90, MeOH:$CHCl_3$); δ_H (500 MHz; $CDCl_3$) 8.46 (1H, s, 2-H pyridyl), 8.29 (1H, d, *J* 4.5, 6-H pyridyl), 7.98 (1H, dd, *J* 8.3, 4-H pyridyl), 7.32 (2H, d, *J* 8.3, Ar), 7.26 (2H, d, *J* 8.3, Ar), 7.20 (1H, m, 5-H pyridyl), 6.44 (1H, s, N-H), 6.34 (1H, quin, *J* 6.2, 4.3, 1.9, 4-H), 5.94 (1H, q, *J* 2.1, 4'-H), 5.79 (1H, s, 5'-H), 4.79 (2H, s, benzyl), 4.57 (1H, d, *J* 14, 2'-H_A), 4.43 (1H, dd, *J* 14.0, 4.9, 2'-H_B), 4.06 (2H, q, *J* 2.3, 6-H), 3.61 (2H, t, *J* 4.7, 2-H), 2.38 (2H, m, 3-H), 2.10 (2H, m, tag), 1.07 (14H, m, tag), 0.92 (2H, m, tag); δ_C (75 MHz; $CDCl_3$) 154.5 (carbonyl), 144.7 (pyridyl), 142.2 (pyridyl), 141.5 (pyridyl), 136.0 (Ar), 135.4 (C-4), 134.2 (C-4'), 127.8 (Ar), 126.6 (Ar), 123.9 (pyridyl), 119.5 (C-3'), 117.9 (C-5), 92.3 (C-8), 81.2 (C-7), 71.5 (C-7'), 64.9 (benzyl), 57.4 (C-2'), 46.4 (C-6), 40.0 (C-2'), 25.8 (C-3), 25.7 (tag), 17.8 (tag), 17.7 (tag), 12.7 (tag), 0.1 (tag); m/z (ES) [M+H] 1093.2 (100%, M+H); HRMS Found: 1092.2299, $C_{41}H_{41}F_{20}N_4O_4S_1Si_1$ requires 1092.2293.

(*S*)-(3-((5-(4-(((3,3,4,4,5,5,6,6,7,7,8,8,9,9,10,10,10-Heptadecafluorodecyl)diisopropylsilyloxy)methyl) phenyl)-1-(trifluoromethylsulfonyl)-2,5-dihydro-1*H*-pyrrol-3-yl)ethynyl)-5,6-dihydropyridin-1(2*H*)-yl)(isoxazol-5-yl)methanone (S28)

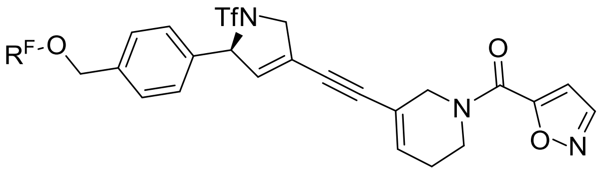

By general procedure **E1**, the fluorous-tagged amine **49** (0.035 g, 0.036 mmol) gave a crude product that was purified by F–SPE to afford the amide **S28** (0.012 g, 32%), R_f 0.32 (30:70, EtOAc:petrol); δ_H (500 MHz; $CDCl_3$) 8.23 (1H, d, *J* 1.7, 5''-H), 7.33 (2H, d, *J* 8.3, Ar), 7.27 (2H, d, *J* 8.3, Ar), 6.85 (1H, d, *J* 1.7, 6''-H^{min}), 6.83 (1H, d, *J* 1.7, 6''-H^{maj}), 6.35 (1H, m, 4-H), 6.01 (1H, bs, 4'-H), 5.79 (1H, s, 5'-H), 4.80 (2H, s, benzyl), 4.57 (2H, d, *J* 14.0, 2'-H^{min}), 4.51 (2H, d, *J* 10, 2'-H^{maj}), 4.30 (2H, d, *J* 18, 6-H), 3.83 (2H, t, *J* 5.5, 2-H^{min}), 3.78 (2H, t, *J* 5.5, 2-H^{maj}), 2.46 (2H, m, 3-H^{maj}), 2.40 (2H, m, 3-H^{min}), 2.13 (2H, m, tag), 1.07 (14H, m, tag), 0.90 (2H, m, tag).

(*S*)-(3-((5-(4-(Hydroxymethyl)phenyl)-1-(trifluoromethylsulfonyl)-2,5-dihydro-1*H*-pyrrol-3-yl)ethynyl)-5,6-dihydropyridin-1(2*H*)-yl)(isoxazol-5-yl)methanone (63b)

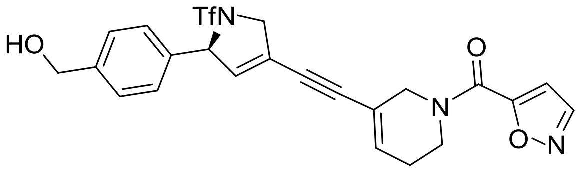

By general method **D**, the fluorous-tagged silyl ether **S28** (0.010 g, 0.01 mmol) gave a crude product that was purified by flash chromatography (gradient elution: 2:98 → 10:90, MeOH–$CHCl_3$) to furnish the *amide* **63b** (0.003 g, 64%), R_f 0.36 (60:40, EtOAc:petrol); δ_H (500 MHz; MeOD) 8.52 (1H, d, *J* 1.7, 5''-H), 7.38 (2H, d, *J* 8.3, Ar), 7.30 (2H, d, *J* 8.3, Ar), 6.88 (1H, s, 6'-H^{min}), 6.86 (1H, d, *J* 1.7, 6'-H^{maj}), 6.40 (1H, s, 4-H), 6.13 (1H, d, *J* 2.1, 4'-H^{maj}), 6.09 (1H, d, *J* 2.1, 4'-H^{min}), 5.86 (1H, s, 5'-H^{maj}), 5.84 (1H, s, 5'-H^{min}), 4.61 (2H, s, benzyl), 4.54 (2H, d, *J* 18.8, 2'-H), 4.26 (2H, q, *J* 2.3, 6-H), 3.83 (2H, t, *J* 5.9, 2-H^{min}), 3.72 (2H, t, *J* 5.9, 2-H^{maj}), 2.43 (2H, m, 3-H^{maj}), 2.39 (2H, m, 3-H^{min}); δ_C (125 MHz; MeOD) 163.9 (carbonyl), 151.7 (C-5''), 143.7 (Ar), 135.9 (C-4), 133.0 (C-4'), 130.0, 128.5 (Ar), 128.3 (Ar), 108.3 (C-6'), 90.7 (C-8), 82.6 (C-7), 72.7 (C-5'), 64.8 (benzyl), 58.3 (C-2'), 45.7 (C-2), 27.3 (C-3); m/z (ES) [M+H] 508.1 (100%, M+H); HRMS Found: 508.1150, $C_{23}H_{21}F_3N_3O_5S_1$ requires 508.1149.

(*S*)-3-((5-(4-(Hydroxymethyl)phenyl)-1-(trifluoromethylsulfonyl)-2,5-dihydro-1*H*-pyrrol-3-yl)ethynyl)-*N*-(pyridin-4-yl)-5,6-dihydropyridine-1(2*H*)-carboxamide (63c)

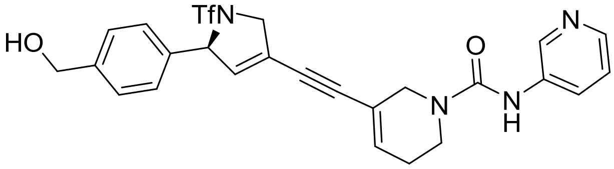

By general method **D**, the fluorous-tagged silyl ether **S27** (0.030 g, 0.027 mmol) gave a crude product which was purified by flash chromatography (gradient elution: 2:98 → 10:90, MeOH–$CHCl_3$) to furnish the *alcohol* **63c** (0.008 g, 58%), R_f 0.32 (10:90, MeOH:$CHCl_3$); δ_H (500 MHz; MeOD) 8.60 (1H, s, 8''-H pyridyl), 8.18 (1H, s, 4''-H pyridyl), 7.92 (1H, d, *J* 7.8, 6''-H pyridyl), 7.37 (2H, d, *J* 8.3, Ar), 7.34 (1H, d, *J* 7.8, 7''-H pyridyl), 7.30 (2H, d, *J* 8.3, Ar), 6.40 (1H, septate, *J* 6.3, 4.2, 2.1, 4-H), 6.09 (1H, q, *J* 2.1, 4'-H), 5.85 (1H, d, *J* 2.1, 5'-H), 4.61 (2H, s, benzyl), 4.54 (2H, s, 2'-H), 4.12 (2H, q, *J* 2.6, 6-H), 3.64 (2H, t, *J* 5.7, 2-H), 2.36 (2H, m, 3-H); δ_C (75 MHz; MeOD) 157.0 (carbonyl), 144.0 (Ar), 143.7 (pyridyl), 142.6 (pyridyl), 136.1 (C-4), 135.6 (C-4'), 129.8 (pyridyl), 128.5 (Ar), 128.3 (Ar), 127.9 (pyridyl), 120.3 (C-3'), 119.5 (C-5), 93.0 (C-8), 81.6 (C-7), 72.7 (C-5'), 64.7 (benzyl), 58.4 (C-2'), 47.1 (C-6), 41.0 (C-2), 26.5 (C-3); m/z (ES) [M+H] 533.1 (100%, M+H); HRMS Found: 533.1473, $C_{25}H_{24}F_3N_4O_4S_1$ requires 533.1465.

(*S*)-(3-((5-(4-(((3,3,4,4,5,5,6,6,7,7,8,8,9,9,10,10,10-Heptadecafluorodecyl)diisopropylsilyloxy)methyl) phenyl)-1-(trifluoromethylsulfonyl)-2,5-dihydro-1*H*-pyrrol-3-yl)ethynyl)-5,6-dihydropyridin-1(2*H*)-yl) (morpholino)methanone (S26)

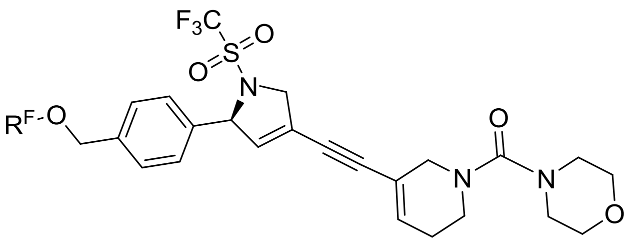

By general method **E3**, the fluorous-tagged amine **49** (0.035 g, 0.036 mmol) gave a crude product which was purified by F–SPE to give the urea **S26** (0.032 g, 84%) R_f 0.68 (5:95, MeOH:CHCl$_3$); δ_H (500 MHz; CDCl$_3$) 7.32 (2H, d, *J* 8.3, Ar), 7.26 (2H, d, *J* 8.3, Ar), 6.32 (1H, m, 4-H), 5.98 (1H, q, *J* 2.2, 4'-H), 5.79 (1H, bs, 5-H), 4.80 (2H, s, benzyl), 4.56 (1H, d, *J* 14, 2'-H$_A$), 4.43 (1H, dd, *J* 14.0, 4.0, 2'-H$_B$), 3.82 (2H, dd, *J* 4.5, 3.0, 6-H), 3.70 (4H, t, *J* 6, 4", 6"-H), 3.34 (2H, t, *J* 4.5, 2-H), 3.28 (4H, t, *J* 6, 3", 7"-H), 2.32 (2H, m, 3-H), 2.11 (2H, m, tag), 1.07 (14H, m, tag), 0.90 (2H, m, tag).

(*S*)-(3-((5-(4-(Hydroxymethyl)phenyl)-1-(trifluoromethylsulfonyl)-2,5-dihydro-1*H*-pyrrol-3-yl)ethynyl)-5,6-dihydropyridin-1(2*H*)-yl)(morpholino)methanone (63d)

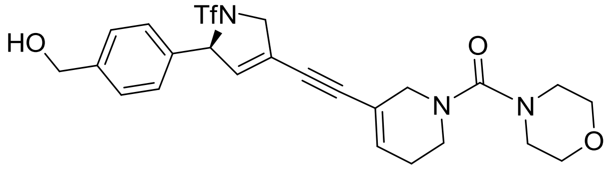

By using general method **D**, the fluorous-tagged silyl ether **S26** (0.022 g, 0.020 mmol) gave a crude product which was purified by flash chromatography (gradient elution: 2:98 → 10:90, MeOH–CHCl$_3$) to the alcohol **63d** (0.008 g, 79%), R_f 0.27 (10:90, MeOH:CHCl$_3$); δ_H (500 MHz; MeOD) 7.38 (2H, d, *J* 8.3, Ar), 7.30 (2H, d, *J* 8.3, Ar), 6.35 (1H, quin, *J* 6.4, 4.2, 2.1, 4-H), 6.09 (1H, q, *J* 2.2, 4'-H), 5.86 (1H, d, *J* 2.2, 5'-H), 4.60 (2H, s, benzyl), 4.54 (2H, s, 2'-H), 3.86 (2H, q, *J* 2.5, 6-H), 3.68 (4H, t, *J* 4.7, 4", 6"-H), 3.37 (2H, t, *J* 5.9, 2-H), 3.27 (4H, t, *J* 4.7, 3", 7"-H), 2.32 (2H, m, 3-H); δ_C (75 MHz; MeOD) 165.2 (carbonyl), 143.6 (Ar), 138.8 (Ar), 136.2 (C-4), 135.5 (C-4'), 128.5 (Ar), 128.3 (Ar), 120.3 (C-3'), 119.8 (C-5), 93.1 (C-8), 81.5 (C-7), 72.7 (C-5'), 67.6 (C-4", 6"), 64.7 (benzyl), 58.4 (C-2'), 49.7 (C-6), 48.5 (C-3", 7"), 43.7 (C-2), 26.1 (C-3); m/z (ES) [M+H] 526.2 (100%, M+H); HRMS Found: 526.1615, $C_{24}H_{27}F_3N_3O_5S_1$ requires 526.1618.

(4-((2*S*,5*R*)-5-(((*S*)-1,2,5,6-Tetrahydropyridin-2-yl)methyl)-1-(trifluoromethylsulfonyl)-2,5-dihydro-1*H*-pyrrol-2-yl)phenyl)methanol (64a)

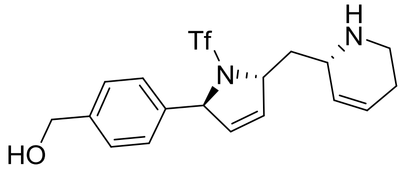

By general method **D**, the fluorous-tagged silyl ether **50** (0.033 g, 0.030 mmol) gave a crude product which was purified by flash chromatography (gradient elution: 2:98 → 10:90, MeOH–CHCl$_3$) to give the alcohol **64a** (0.012 g, 87%) R_f 0.29 (20:80, MeOH:CHCl$_3$); δ_H (500 MHz; MeOD) 7.34 (2H, d, *J* 8.3, Ar), 7.29 (2H,

d, *J* 8.3, Ar), 6.23 (1H, dt, *J* 6.4, 1.7, 4'-H), 5.94 (1H, m, 4-H), 5.88 (1H, d, *J* 6.4, 3'-H), 5.69 (1H, d, *J* 10.7, 5-H), 5.15 (1H, s, 2'-H), 4.60 (2H, s, benzyl), 3.81 (1H, s, 6-H), 3.26 (1H, m, 2-H_A), 3.08 (1H, m, 2-H_B), 2.50 (1H, dt, *J* 8.9, 3.0, 7-H_A), 2.31 (1H, m, 4-H_A), 2.20 (1H, d, *J* 17.3, 4-H_B), 2.11 (1H, m, 7-H_B); δ_C (100 MHz; MeOD) 143.7 (Ar), 131.6 (C-3'), 128.4 (C-4'), 127.9 (Ar), 127.3 (C-5), 125.9 (Ar), 122.7 (C-4), 73.5 (C-5'), 67.8 (C-2'), 64.7 (benzyl), 51.7 (C-6), 41.8 (C-2), 40.3 (C-7), 23.9 (C-3); m/z (ES) [M+H] 403.1 (100%, M+H); HRMS Found: 403.1324, $C_{18}H_{22}F_3N_2O_3S_1$ requires 403.1309.

((*S*)-2-(((2*R*,5*S*)-5-(4-(Hydroxymethyl)phenyl)-1-(trifluoromethylsulfonyl)-2,5-dihydro-1*H*-pyrrol-2-yl)methyl)-5,6-dihydropyridin-1(2*H*)-yl)(isoxazol-5-yl) methanone (64b)

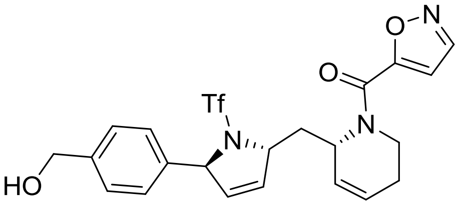

By general method **E1**, the fluorous-tagged amine **50** (0.055 g, 0.057 mmol) gave a crude product, which was purified by F–SPE and subjected to general method **D** to give another crude product, which was purified by flash chromatography (gradient elution: 10:90 → 30:70, ethyl acetate–petrol) to furnish the amide **64b** (0.008 g, 29%). R_f 0.44 (70:30, EtOAc:petrol); δ_H (500 MHz; MeOD) 8.53 (1H, d, *J* 2.1, 5''-H), 7.33 (2H, d, *J* 8.1, Ar), 7.27 (2H, d, *J* 8.1, Ar), 6.84 (1H, s, 6''-H), 6.34 (1H, m, 4'-H), 5.92 (1H, dd, *J* 9.4, 4.7, 4-H), 5.75 (3H, m, 5-H, 3-H, 3'-H), 5.18 (1H, dd, *J* 11.1, 2.1, 6-H), 4.94 (1H, m, 2'-H), 4.59 (2H, s, benzyl), 3.92 (1H, dd, *J* 14.1, 5.9, 2-H_A), 3.48 (1H, dt, *J* 11.9, 3.8, 2-H_B), 2.68 (1H, t, *J* 11.1, 7-H_A), 2.41 (1H, m, 3-H_A), 2.15 (1H, td, *J* 17.5, 4.7, 3-H_B), 2.08 (1H, m, 7-H_B); δ_C (100 MHz; MeOD) 164.5, 160.7, 152.0, 129.2, 128.3, 127.2, 107.6, 73.9, 68.8, 65.1, 41.6, 39.4, 27.5; m/z (ES) [M+H] 498.1 (100%, M+H); HRMS Found: 498.1325, $C_{22}H_{23}F_3N_3O_5S_1$ requires 498.1305.

(*S*)-2-(((2*R*,5*S*)-5-(4-(Hydroxymethyl)phenyl)-1-(trifluoromethylsulfonyl)-2,5-dihydro-1*H*-pyrrol-2-yl)methyl)-*N*-(pyridin-3-yl)-5,6-dihydropyridine-1(2*H*)-carboxamide (64c)

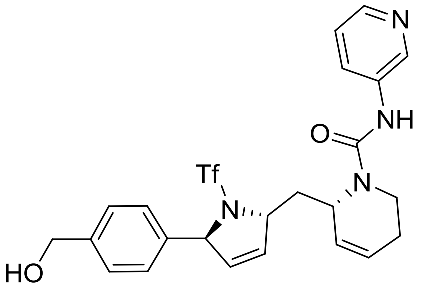

By general method **E2**, the fluorous-tagged amine **50** (0.041 g, 0.04 mmol) gave a crude product, which was purified by F–SPE and subjected to general method **D** to give a crude product, which was purified by flash chromatography (gradient elution: 02:98 → 10:90, MeOH–$CHCl_3$) to furnish the urea **64c** (0.009 g, 43%), R_f 0.56 (20:80, MeOH:$CHCl_3$); δ_H (500 MHz; MeOD) 8.61 (1H, d, *J* 2.5, pyridyl), 8.19 (1H, dd, *J* 4.7, 1.2, pyridyl), 7.92 (1H, ddd, *J* 8.3, 2.5, 1.2, pyridyl), 7.37 (1H, ddd, *J* 8.3, 4.7, 1.2, pyridyl), 7.31 (2H, d, *J* 8.3,

Ar), 7.24 (2H, d, *J* 8.3, Ar), 6.34 (1H, d, *J* 6.6, 4'-H), 5.91 (1H, dd, *J* 10.0, 6.0, 5-H), 5.73 (3H, m, 3', 4, 5'-H), 4.93 (1H, t, *J* 10, 6-H), 4.58 (2H, s, benzyl), 4.07 (1H, dd, *J* 14.5, 5.7, 2-H_A), 3.30 (2H, m, 2-H_B, 2'-H), 2.67 (1H, dt, *J* 11., 2.5, 7-H_A), 2.30 (1H, dq, *J* 17.7, 11.3, 2.5, 3-H_A), 2.07 (1H, dt, *J* 17.7, 4.2, 3-H_B), 1.92 (1H, m, 7-H_B); δ_C (100 MHz; MeOD) 157.3 (carbonyl), 144.0 (pyridyl), 142.9 (pyridyl), 138.7 (Ar), 130.2 (Ar), 128.2 (C-4'), 127.8 (C-3'), 126.9 (Ar), 125.0 (Ar), 122.8 (C-5), 119.6 (C-4), 73.5 (C-5'), 68.5 (C-6), 64.7 (benzyl), 49.9 (C-2'), 39.1 (C-7), 38.8 (C-2), 26.0 (C-3); m/z (ES) [M+H] 523.2 (100%, M+H); HRMS Found: 523.1626, $C_{24}H_{26}F_3N_4O_4S_1$ requires 523.1611.

((*S*)-2-(((2*R*,5*S*)-5-(4-(Hydroxymethyl)phenyl)-1-(trifluoromethylsulfonyl)-2,5-dihydro-1*H*-pyrrol-2-yl)methyl)-5,6-dihydropyridin-1(2*H*)-yl)(morpholino)methanone (64d)

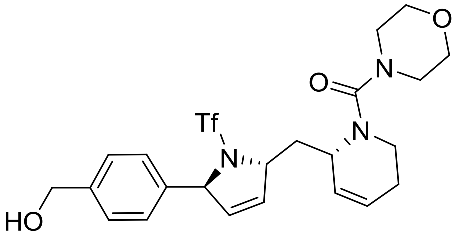

By general method **E3**, the fluorous-tagged amine **50** (0.041 g, 0.04 mmol) gave a crude product, which was purified by F–SPE and subjected to general method **D** to give a crude product, which was purified by flash chromatography (gradient elution: 2:98 → 10:90, MeOH–$CHCl_3$) to furnish the urea **64d** (0.007 g, 34%), R_f 0.31 (10:90, MeOH:$CHCl_3$); δ_H (500 MHz; CD_3OD) 7.34 (2H, d, *J* 8.3, Ar), 7.26 (2H, d, *J* 8.3, Ar), 6.19 (1H, dt, *J* 8.3, 1.8, 4'-H), 5.87 (1H, m, 4-H), 5.76 (1H, dt, *J* 8.3, 3'-H), 5.72 (2H, m, 5, 5'-H), 4.88 (1H, m, 6-H), 4.68 (1H, d, *J* 10.3, 2'-H), 4.61 (2H, s, benzyl), 3.71 (5H, m, 4", 6"-H, 2-H_A), 3.33-3.16 (5H, m, 3", 7"-H, 2-H_B), 2.39 (1H, t, J 12.5, 7-H_A), 2.10 (1H, m, 3-H_A), 1.91 (1H, dt, *J* 21.0, 3.6, 3-H_B), 1.82 (1H, m, 7-H_B); δ_C (100 MHz; CD_3OD) 166.0, 129.6, 127.8, 126.3, 67.7, 64.7, 41.8, 39.7, 26.0; m/z (ES) [M+H] 560.2 (100%, M+H); HRMS Found: 516.1789, $C_{23}H_{29}F_3N_3O_5S_1$ requires 516.1775.

(4-((2*S*,5*S*)-5-(((*R*)-1-(Trifluoromethylsulfonyl)-2,3,6,7-tetrahydro-1*H*-azepin-3-yl)methyl)-1,2,5,6-tetrahydropyridin-2-yl)phenyl)methanol (65a)

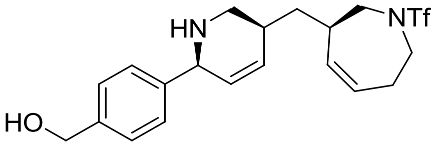

By general method **D**, the fluorous-tagged silyl ether **51** (0.050 g, 0.05 mmol) gave a crude product which was purified by flash chromatography to furnish the alcohol **65a** (0.015 g, 70%), R_f 0.56 (20:80, MeOH:$CHCl_3$); δ_H (500 MHz; MeOD) 7.31 (4H, s, Ar), 5.84 (1H, d, *J* 9.8, 5'-H), 5.80 (1H, dd, *J* 11.1, 5.5, 5-H), 5.71 (2H, m, 4, 4'-H), 4.56 (1H, s, 6'-H), 4.50 (2H, s, benzyl-CH_2), 3.64 (2H, d, *J* 13.5, 7-CH_2), 3.50 (1H, m, 2-CH_2-H_A), 3.41 (1H, dd, *J* 13.5, 9.0, 2-CH_2-H_B), 3.21 (1H, dd, *J* 12.0, 5.0, 2'-CH_2-H_A), 2.65 (2H, m, 2'-CH_2-H_B, 3'-H), 2.44 (2H, m, 3-CH_2), 2.38 (1H, m, 6-H), 1.46 (2H, m, 8-CH_2); δ_C (75 MHz; MeOD) 143.4, 140.6, 132.4, 130.5, 129.5, 129.3, 128.7, 124.1, 65.0, 59.5, 54.5, 50.9, 32.6, 31.2; m/z (ES) [M+H] 431.2 (100%, M+H); HRMS Found: 431.1616, $C_{20}H_{26}F_3N_2O_3S_1$ requires 431.1611.

((2*S*,5*S*)-2-(4-(Hydroxymethyl)phenyl)-5-(((*R*)-1-(trifluoromethylsulfonyl)-2,3,6,7-tetrahydro-1*H*-azepin-3-yl)methyl)-5,6-dihydropyridin-1(2*H*)-yl)(isoxazol-5-yl)methanone (65b)

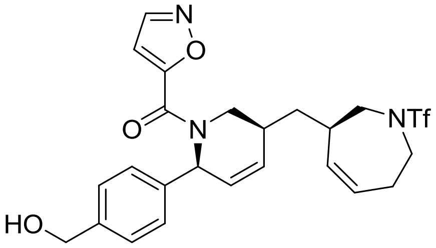

By general method **E1**, the fluorous-tagged amine **51** (0.060 g, 0.06 mmol) gave a crude product, which was purified by F–SPE to afford a crude product. This was subjected to general method **D** to give a crude product, which was purified by flash chromatography to furnish the amide **65b** (0.013 g, 40%), R_f 0.42 (10:90, MeOH:CHCl$_3$); δ_H (500 MHz; CD$_3$OD) 8.40 (1H, s, isoxazole), 7.36 (2H, d, *J* 8.3, Ar), 7.26 (2H, d, *J* 8.3, Ar), 6.71 (1H, s, isoxazole), 6.04 (1H, s,), 5.87 (1H, s,), 5.72 (1H, s,), 5.57 (1H, s,), 4.50 (2H, s, benzyl-CH$_2$), 3.75 (1H, d, *J* 14.1), 3.44 (1H, d, *J* 14.1), 3.52 (4H, m,), 2.31 (4H, bs,), 1.43 (2H, bs,); δ_C (75 MHz; CD$_3$OD) 160.32, 152.2, 139.6, 130.5, 129.5, 128.6, 127.9, 127.5, 108.3, 65.1, 55.4, 54.3, 45.1, 34.4, 31.0; ν_{max}/cm^{-1} (film) 3420, 2927, 1634, 1579, 1511, 1478; m/z (ES) [M+H] 526.1 (20%, M+H), 543.2 (50%, M+NH$_4$), 548.1 (30%, M+Na); HRMS Found: 543.1873, $C_{24}H_{30}F_3N_4O_5S_1$ requires 543.1884.

(2*S*,5*S*)-2-(4-(Hydroxymethyl)phenyl)-*N*-(pyridin-3-yl)-5-(((*R*)-1-(trifluoromethylsulfonyl)-2,3,6,7-tetrahydro-1*H*-azepin-3-yl)methyl)-5,6-dihydropyridine-1(2*H*)-carboxamide (65c)

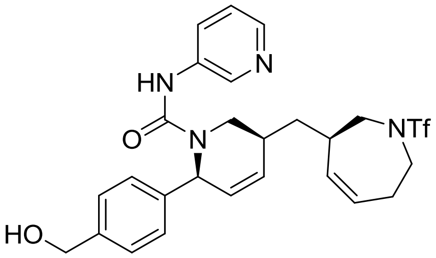

By general method **E2**, the fluorous-tagged amine **51** (0.041 g, 0.04 mmol) gave a crude product, which was purified by F–SPE and subjected to general method **D** to give another crude product, which was purified by flash chromatography to furnish the urea **65c** (0.025 g, 82%), R_f 0.76 (20:80, MeOH:CHCl$_3$); δ_H (500 MHz; CD$_3$OD) 8.49 (1H, s, pyridyl), 8.09 (1H, s, pyridyl), 7.81 (1H, d, *J* 8.3, pyridyl), 7.29 (1H, m, pyridyl), 7.26 (2H, d, *J* 8.3, Ar), 7.23 (2H, d, *J* 8.3, Ar), 6.01 (1H, m), 5.88 (1H, dd, *J* 10.2, 4.2), 5.74 (1H, m), 5.71 (1H, m), 5.64 (1H, bs), 4.47 (2H, s, benzyl-CH$_2$), 3.99 (1H, d, *J* 14.1), 3.49 (4H, m), 3.15 (1H, dd, *J* 14.1, 4.2), 2.59 (1H, s), 2.29 (3H, m), 1.47 (2H, s); δ_C (75 MHz; CD$_3$OD) 157.8, 143.4, 142.5, 142.1, 140.3, 131.2, 130.3, 128.6, 128.5, 125.6, 124.0, 65.1, 55.9, 54.6, 42.7, 34.3, 31.2; ν_{max}/cm^{-1} (film) 3321, 2925, 1708, 1660, 1644, 1532; m/z (ES) [M+H] 551.2 (100%, M+H); HRMS Found: 551.1944, $C_{26}H_{30}F_3N_4O_4S_1$ requires 551.1934.

((2*S*,5*S*)-2-(4-(Hydroxymethyl)phenyl)-5-(((*R*)-1-(trifluoromethylsulfonyl)-2,3,6,7-tetrahydro-1*H*-azepin-3-yl)methyl)-5,6-dihydropyridin-1(2*H*)-yl)(morpholino)methanone (65d)

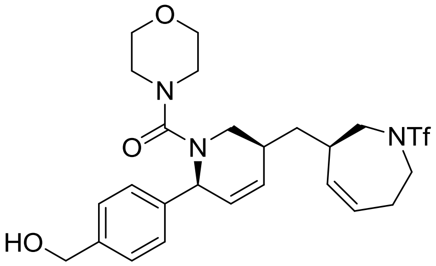

By general method **E3**, the fluorous-tagged amine **51** (0.060 g, 0.06 mmol) gave a crude product, which was purified by F–SPE and subjected to general method **D**. This give a crude product, which was purified by flash chromatography to furnish the urea **65d** (0.024 g, 74%), R_f 0.49 (10:90, MeOH:CHCl$_3$); δ_H (500 MHz; CD$_3$OD) 7.33 (2H, d, *J* 8.3, Ar), 7.25 (2H, d, *J* 8.3, Ar), 6.03 (1H, dd, *J* 9.4, 5.5), 5.83 (1H, dd, *J* 10.2, 4.2), 5.75 (2H, m), 5.65 (1H, s), 5.56 (1H, s), 4.49 (2H, s, benzyl-CH$_2$), 3.99 (1H, d, *J* 11.1), 3.75 (1H, d *J* 13.2,), 3.56 (4H, m,), 3.48 (1H, m), 3.39 (2H, m), 3.31 (2H, dd, *J* 14.1, 3.4), 2.31 (3H, m), 1.48 (1H, m,), 1.18 (1H, m); δ_C (75 MHz; CD$_3$OD) 152.2, 142.6, 139.2, 131.3, 129.8, 128.9, 128.4, 128.2, 127.2, 67.3, 64.7, 57.8, 56.7, 54.2, 50.6, 46.7, 45.5, 33.7, 30.9; ν_{max}/cm^{-1} (film) 3417, 2923, 2862, 1755, 1634; m/z (ES) [M+H] 544.2 (100%, M+H); HRMS Found: 544.2096, $C_{25}H_{33}F_3N_3O_5S_1$ requires 544.2088.

(1*R*,9*S*,12*S*,*E*)-9-(4-(Hydroxymethyl)phenyl)-3,10-diazabicyclo[10.2.1]pentadeca-7,13-dien-4-one (66a)

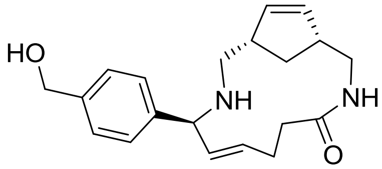

By general method **D**, the fluorous-tagged silyl ether **53** (0.042 g, 0.047 mmol) gave a crude product, which was purified by flash chromatography (gradient elution: 2:98 → 10:90, MeOH–CHCl$_3$) to furnish the alcohol **66a** (0.008 g, 52%), R_f 0.25 (20:80, MeOH:CHCl$_3$); δ_H (500 MHz; MeOD) 7.33 (4H, s, Ar), 5.80 (1H, dt, *J* 5.5, 2.1, 11-H), 5.73 (2H, m, 12, 5-H), 5.50 (1H, dd, *J* 15.8, 7.5, 6-H), 4.58 (2H, s, benzyl), 4.33 (1H, d, *J* 7.5, 7-H), 3.42 (1H, dd, *J* 13.9, 4.2, 9-H$_A$), 3.22 (1H, dd, *J* 13.9, 4.2, 9-H$_B$), 2.99 (1H, d, *J* 7.2, 10-H), 2.83 (1H, t, *J* 7.2, 13-H), 2.75 (2H, dq, *J* 11.3, 7.2, 14-H), 2.38 (2H, dq, *J* 12.6, 7.2, 3-H), 2.33 (2H, dd, *J* 12.6, 7.2, 4-H), 2.20 (1H, dt, *J* 14.3, 9.6, 15-H$_A$), 1.48 (1H, dt, *J* 14.3, 2.5, 15-H$_B$); δ_C (75 MHz; MeOD) 176.3 (carbonyl), 142.7 (Ar), 141.7 (Ar), 135.7 (C-5), 134.9 (C-12), 134.6 (C-11), 132.2 (C-6), 129.0 (Ar), 128.6 (Ar), 65.3 (benzyl), 64.9 (C-2), 52.5 (C-14), 47.9 (C-10), 46.3 (C-13), 44.0 (C-9), 38.4 (C-3), 30.9 (C-15), 30.0 (C-4); m/z (ES) [M+H] 327.2 (100%, M+H); HRMS Found: 327.2069, $C_{20}H_{27}N_2O_2$ requires 327.2067.

(*R*)-(1'-(Trifluoromethylsulfonyl)-1,1',2,2',5,5',6,6'-octahydro-3,3'-bipyridin-6-yl)methanol (67a)

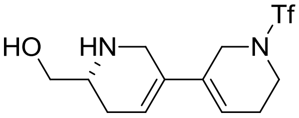

By general method **D**, the fluorous-tagged silyl ether **54** (0.212 g, 0.23 mmol) gave a crude product which was purified by flash chromatography (gradient elution: 02:98 → 10:90, MeOH–$CHCl_3$) to furnish the alcohol **67a** (0.070 g, 91%), R_f 0.20 (20:80, MeOH:$CHCl_3$); δ_H (500 MHz; MeOD) 5.99 (1H, s, 4-H), 5.95 (1H, s, 4'-H), 4.64 (2H, s, 6-CH_2), 3.90 (2H, t, *J* 16.2, 2-CH_2), 3.88 (1H, dd, *J* 12.4, 3.8, 7'-CH_2-H_A), 3.64 (1H, dd, *J* 12.0, 6.4, 7'-CH_2-H_B), 3.63 (1H, bs, 2'-CH_2-H_A), 3.43 (1H, m, 6'-H), 3.33 (1H, s, 2'-CH_2-H_B), 2.47 (2H, m, 5-CH_2), 2.43 (2H, s, 3-CH_2); δ_C (75 MHz; MeOD) 131.2 (C-5), 129.0 (C-3'), 123.6 (C-4), 121.4 (C-4'), 62.1 (C-7'), 55.4 (C-6'), 46.4 (C-6), 44.5 (C-2), 43.5 (C-2'), 26.6 (C-3), 25.5 (C-5'); ν_{max}/cm^{-1} (film) 3400, 1634, 1432, 1385, 1319; m/z (ES) [M+H] 327.1 (100%, M+H); HRMS Found: 327.0996, $C_{12}H_{18}F_3N_2O_3S_1$ requires 327.0985.

(*R*)-(6-(Hydroxymethyl)-1'-(trifluoromethylsulfonyl)-1',2',5,5',6,6'-hexahydro-3,3'-bipyridine-1(2*H*)-yl)(isoxazol-5-yl)methanone (67b)

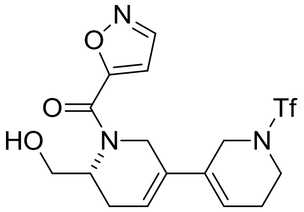

By general method **E4**, the fluorous-tagged amine **54** (0.125 g, 0.15 mmol) gave a crude product, which was purified by F–SPE and subjected to general method **D** to give a crude product, which was purified by flash chromatography to furnish the amide **67b** (0.060 g, 77%), R_f 0.40 (10:90, MeOH:$CHCl_3$); δ_H (500 MHz; MeOD) 8.52 (1H, s, isoxazole), 6.86 (1H, s, isoxazole), 6.07 (1H, s, 4-H), 5.85 (1H, s, 4'-H), 4.88 (1H, d, *J* 12, 2'-CH_2-H_A), 4.32 (1H, m, 6'-H), 4.25 (2H, s, 6-CH_2), 3.84 (1H, d, *J* 18.8, 2'-CH_2-H_B), 3.66 (3H, m, 2-CH_2, 7'-CH_2-H_A), 3.48 (1H, dd, *J* 12.0, 5.3, 7'-CH_2-H_B), 2.68 (1H, d, *J* 18.8, 5'-CH_2-H_A), 2.45 (2H, s, 3-CH_2), 2.24 (1H, dd, *J* 18.8, 5.3, 5'-CH_2-H_B); δ_C (75 MHz; MeOD) 164.6 (carbonyl), 152.0 (isoxazole), 131.6, 130.8, 122.2 (C-4), 120.2 (C-4'), 61.8 (C-7'), 55.6 (C-6'), 46.5 (C-6), 45.1 (C-2), 40.4 (C-2'), 27.4 (C-5'), 26.7 (C-3); ν_{max}/cm^{-1} (film) 3401, 2907, 2152, 1625, 1576, 1426; m/z (ES) [M+H] 422.1 (100%, M+H); HRMS Found: 422.0997, $C_{16}H_{19}F_3N_3O_5S_1$ requires 422.0992.

(*R*)-6-(Hydroxymethyl)-*N*-(pyridin-3-yl)-1'-(trifluoromethylsulfonyl)- 1',2',5,5',6,6'-hexahydro-3,3'-bipyridine-1(2*H*)-carboxamide (67c)

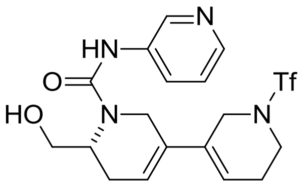

By using general procedure **E2**, the fluorous-tagged amine **54** (0.077 g, 0.086 mmol) gave a crude product, which was purified by F–SPE and subjected to general method **D** to give another crude product, which was purified by flash chromatography (gradient elution: 2:98 → 10:90, MeOH–$CHCl_3$) to furnish the urea **67c** (0.029 g, 83%), R_f 0.63 (20:80, MeOH:$CHCl_3$); δ_H (500 MHz; MeOD) 8.62 (1H, s, pyridyl), 8.20 (1H, d, *J*

4.7, pyridyl), 7.94 (1H, d, *J* 8.3, pyridyl), 7.38 (1H, dd, *J* 8.3, 4.7, pyridyl), 6.08 (1H, t, *J* 4.2, 4-H), 5.85 (1H, d, *J* 4.7, 4'-H), 4.56 (1H, dd, *J* 14.5, 4.5, 6'-H), 4.53 (1H, d, *J* 16.6, 2'-CH_2-H_A), 4.24 (2H, s, 6-CH_2), 3.89 (1H, d, *J* 16.6, 2'-CH_2-H_B), 3.66 (2H, m, 2-CH_2), 3.62 (1H, dd, *J* 11.4, 8.3, 7-CH_2-H_A), 3.54 (1H, dd, *J* 10.9, 6.2, 7-CH_2-H_B), 2.58 (1H, d, *J* 18.2, 5'-CH_2-H_A), 2.44 (2H, s, 3-CH_2), 2.31 (1H, dd, *J* 18.7, 6.2, 5'-CH_2-H_B); δ_C (75 MHz; MeOD) 158.4 (carbonyl), 144.2 (pyridyl), 143.0 (pyridyl), 131.9, 131.6, 130.3, 125.3, 121.7 (C-4), 120.4 (C-4'), 62.2 (C-7'), 51.7 (C-6'), 48.4 (C-6), 44.6 (C-2), 41.7 (C-2'), 26.8 (C-5'), 26.7 (C-3); ν_{max}/cm^{-1} (film) 3390, 2767, 1693, 1543, 1465, 1385; m/z (ES) [M+H] 447.1 (100%, M+H); HRMS Found: 447.1299, $C_{18}H_{22}F_3N_4O_4S_1$ requires 447.1308.

(*R*)-(6-(Hydroxymethyl)-1'-(trifluoromethylsulfonyl)-1',2',5,5',6,6'-hexahydro-3,3'-bipyridine-1(2*H*)-yl)(morpholino)methanone (67d)

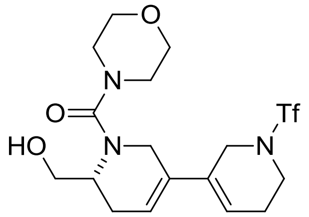

By general method **E3**, the fluorous-tagged amine **54** (0.080 g, 0.07 mmol) gave a crude product, which was purified by F–SPE and subjected to general method **D** to give another crude product, which was purified by flash chromatography (gradient elution: 2:98 → 10:90, MeOH–$CHCl_3$) to furnish the urea **67d** (0.018 g, 42%), R_f 0.21 (10:90, MeOH:$CHCl_3$); δ_H (500 MHz; MeOD) 5.97 (1H, s, 4-H), 5.77 (1H, d, *J* 5.1, 4'-H), 4.20 (2H, s, 6-CH_2), 4.07 (2H, m, 2'-CH_2-H_A, 6'-H), 3.86 (1H, d, *J* 17.1, 2'-CH_2-H_B), 3.65 (7H, m, CH_2-morpholine, 2-CH_2, 7'-CH_2-H_A), 3.50 (1H, dd, *J* 11.5, 6.4, 7'-CH_2-H_B), 3.38 (2H, ddd, *J* 13.2, 6.2, 2.9, CH_2-morpholine), 3.24 (2H, ddd, *J* 13.2, 6.2, 2.9, CH_2-morpholine), 2.59 (1H, d, *J* 18.5, 5'-CH_2-H_A), 2.42 (2H, s, 3-CH_2), 2.15 (1H, dd, *J* 18.5, 5.1, 5'-CH_2-H_B); δ_C (75 MHz; MeOD) 166.6 (carbonyl), 132.1, 132.0, 121.4 (C-4), 120.9 (C-4'), 67.9 (morpholine), 62.0 (C-7'), 54.5 (C-6'), 48.9 (morpholine), 46.5 (C-6), 44.6 (C-2), 43.7 (C-2'), 26.6 (C-3), 26.5 (C-5'); ν_{max}/cm^{-1} (film) 3419, 2900, 2895, 1750, 1621, 1462; m/z (ES) [M+H] 440.1 (M+H, 100%); HRMS Found: 440.1465, $C_{17}H_{25}F_3N_3O_5S_1$ requires 440.1462.

(*R*)-6-(6-(Hydroxymethyl)-1,2,5,6-tetrahydropyridin-3-yl)-3,4-dihydro-1*H*-azepin-2(7*H*)-one (68a)

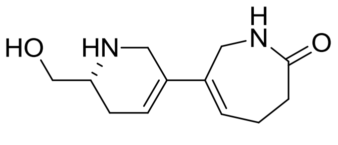

By general method **D**, the fluorous-tagged silyl ether **55** (0.056 g, 0.071 mmol) gave a crude product which was purified by flash chromatography to furnish the alcohol **68a** (0.015 g, 94%), R_f 0.05 (30:70, MeOH:$CHCl_3$); δ_H (500 MHz; MeOD) 5.96 (1H, s, 4'-H), 5.77 (1H, t, *J* 4.7, 5-H), 4.01 (2H, s, 7-CH_2), 3.87 (3H, s, 2'-CH_2, 7'-CH_2-H_A), 3.63 (1H, dd, *J* 11.7, 6.0, 7'-CH_2-H_B), 3.38 (1H, s, 6'-H), 2.69 (2H, dd, *J* 11.7, 6.8, 3-CH_2), 2.51 (2H, m, 4-CH_2), 2.43 (2H, s, 5'-CH_2); δ_C (75 MHz; MeOD) 179.7 (carbonyl), 136.4 (C-3'), 132.1 (C-6), 128.4 (C-5), 121.4 (C-4'), 62.1 (C-7'), 55.3 (C-6'), 44.4 (C-2'), 41.0 (C-7), 33.5 (C-3), 25.9 (C-

4), 25.7 (C-5'); ν_{max}/cm^{-1} (film) 3369, 1639, 1461, 1432, 1349; m/z (ES) [M+H] 222.3 (100%, M+H); HRMS Found: 223.1440, $C_{12}H_{19}N_2O_2$ requires 223.1447.

(*R*)-6-(Hydroxymethyl)-3-(7-oxo-2,5,6,7-tetrahydro-1*H*-azepin-3-yl)-*N*-(pyridin-3-yl)-5,6-dihydropyridine-1(2*H*)-carboxamide (68c)

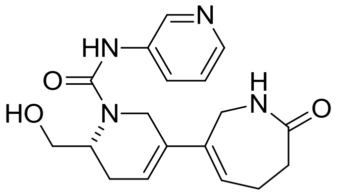

By general method **E2**, the fluorous-tagged amine **55** (0.034 g, 0.043 mmol) gave a crude product, which was purified by F–SPE and subjected to general method **D** to give another crude product which was purified by flash chromatography (gradient elution: 2:98 → 10:90, MeOH–$CHCl_3$) to furnish the urea **68c** (0.009 g, 67%), R_f 0.64 (20:80, MeOH:$CHCl_3$); δ_H (500 MHz; MeOD) 8.27 (1H, s, pyridyl), 8.11 (1H, s, pyridyl), 7.55 (1H, d, *J* 7.5, pyridyl), 7.30 (1H, m, pyridyl), 5.95 (1H, d, *J* 5.5, 4'-H), 5.89 (1H, t, *J* 4.2, 5-H), 4.66 (1H, t, *J* 8.1, 2'-CH_2-H_A), 4.42 (1H, d, *J* 17.1, 2'-CH_2-H_B), 4.16 (1H, dd, *J* 8.1, 6.8, 7'-CH_2-H_A), 4.07 (2H, s, 7-CH_2), 3.87 (2H, m, 6'-H, 7'-CH_2-H_B), 2.73 (2H, dd, *J* 7.7, 5.5, 3-CH_2), 2.56 (2H, m, 4-CH_2), 2.51 (1H, m, 5'-CH_2-H_A), 2.31 (1H, dd, *J* 17.1, 8.1, 5'-CH_2-H_B); δ_C (75 MHz; MeOD) 179.8 (carbonyl amide), 156.8 (carbonyl urea), 146.1 (pyridyl), 143.4 (pyridyl), 136.9 (C-3'), 135.3 (C-6), 133.0 (pyridyl), 127.4 (C-5), 120.7 (C-4'), 73.4 (C-7'), 53.1 (C-6'), 43.8 (C-2'), 41.3 (C-7), 33.5 (C-3), 30.5 (C-5'), 25.9 (C-4'); m/z (ES) [M-OH] 325.2 (100%, M-OH); HRMS Found: 325.1667, $C_{18}H_{21}N_4O_2$ requires 325.1659.

(*R*)-6-(6-(Hydroxymethyl)-1-(morpholine-4-carbonyl)-1,2,5,6-tetrahydropyridin-3-yl)-3,4-dihydro-1*H*-azepin-2(7*H*)-one (68d)

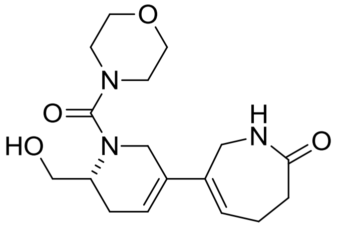

By general method **E3**, the fluorous-tagged amine **55** (0.036 g, 0.046 mmol) gave a crude product, which was purified by F–SPE and subjected to general method **D** to give another crude product, which was purified by flash chromatography (gradient elution: 2:98 → 10:90, MeOH–$CHCl_3$) to furnish the urea **68d** (0.006 g, 40%), R_f 0.27 (1:9, MeOH:$CHCl_3$); δ_H (500 MHz; MeOD) 5.81 (1H, d, *J* 4.7, 4'-H), 5.78 (1H, t, *J* 4.4, 5-H), 4.05 (1H, m, 2'-CH_2-H_A), 4.01 (2H, s, 7-CH_2), 3.84 (2H, m, 2'-CH_2-H_B, 6'-H), 3.70 (4H, m, morpholine), 3.62 (1H, dd, *J* 11.1, 8.9, 7'-CH_2-H_A), 3.49 (1H, dd, *J* 11.1, 6.0, 7'-CH_2-H_B), 3.36 (2H, m, morpholine), 3.23 (2H, m, morpholine), 2.70 (2H, dd, *J* 11.1, 6.6, 3-CH_2), 2.58 (1H, m, 5'-CH_2-H_A), 2.54 (2H, m, 4-CH_2), 2.12 (1H, dd, *J* 18.6, 4.4, 5'-CH_2-H_B); δ_C (75 MHz; MeOD) 179.8 (carbonyl amide), 166.6 (carbonyl morpholine), 137.3 (C-3'), 135.1 (C-6), 126.5 (C-5), 120.7 (C-4'), 67.9 (morpholine), 62.0 (morpholine), 54.4 (C-6'), 49.0

(morpholine), 44.5 (C-2'), 41.2 (C-7'), 33.6 (C-3), 26.6 (C-5'), 25.8 (C-4); m/z (ES) [M+H] 336.2 (100%, M+H); HRMS Found: 336.1921, $C_{17}H_{26}N_3O_4$ requires 336.1918.

(*R*)-(5-((1-(Trifluoromethylsulfonyl)-1,2,5,6-tetrahydropyridin-3-yl)ethynyl)-1,2,3,6-tetrahydropyridin-2-yl)methanol (69a)

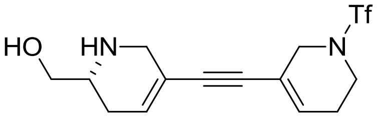

By general method **D**, the fluorous-tagged silyl ether **56** (0.065 g, 0.071 mmol) gave a crude product which was purified by flash chromatography to give the alcohol **69a** (0.022 g, 91%), R_f 0.49 (20:80, MeOH:$CHCl_3$); δ_H (500 MHz; MeOD) 6.25 (1H, quin, *J* 2.1, 4-H), 6.22 (1H, d, *J* 1.7, 4'-H), 4.02 (2H, s, 6-CH_2), 3.65 (2H, s, 2'-CH_2), 3.61 (1H, dd, *J* 10.7, 4.7, 7'-CH_2-H_A), 3.49 (1H, dd, *J* 10.7, 6.8, 7'-CH_2-H_B), 3.45 (1H, dd, *J* 16.6, 3.4, 2'-CH_2-H_A), 3.39 (1H, d, *J* 16.6, 2'-CH_2-H_B), 2.84 (1H, m, 6'-H), 2.39 (2H, s, 3-CH_2), 2.19 (1H, dd, *J* 18.8, 4.7, 5'-CH_2-H_A), 2.02 (1H, m, 5'-CH_2-H_B); δ_C (125 MHz; MeOD) 134.3 (C-4'), 133.2 (C-4), 121.2 (C-5), 118.9 (C-3'), 89.7 (C-8), 86.9 (C-7), 65.9 (C-7'), 54.7 (C-6'), 48.6 (C-6), 48.5 (C-2'), 44.1 (C-2), 28.9 (C-5'), 26.7 (C-3'); ν_{max}/cm^{-1} (film) 3308, 2894, 2833, 1455, 1435; m/z (ES) [M+H] 351.1 (100%, M+H); HRMS Found: 351.0996, $C_{14}H_{18}F_3N_2O_3S_1$ requires 351.0985.

(*R*)-(6-(Hydroxymethyl)-3-((1-(trifluoromethylsulfonyl)-1,2,5,6-tetrahydropyridin-3-yl)ethynyl)-5,6-dihydropyridin-1(2*H*)-yl)(isoxazol-5-yl)methanone (69b)

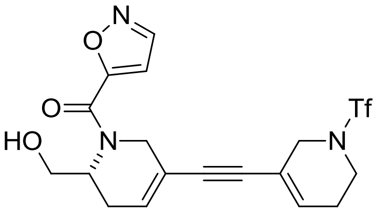

By general method **E4**, the fluorous-tagged amine **56** (0.080 g, 0.087 mmol) gave a crude product, which was purified by F–SPE and subjected to general procedure **D** to give another crude product, which was purified by flash chromatography to furnish the amide **69b** (0.023 g, 67%), R_f 0.40 (80:20, EtOAc:petrol); δ_H (500 MHz; CD_3OD) 8.39 (1H, d, *J* 1.6, isoxazole), 6.74 (1H, d, *J* 1.6, isoxazole), 6.20 (1H, s, 4-H), 6.11 (1H, d, *J* 6.4, 4'-H), 4.52 (1H, d, *J* 18.7, 2'-CH_2-H_A), 4.21 (1H, m, 6'-H), 3.93 (3H, m, 6-CH_2, 2'-CH_2-H_B), 3.59 (1H, dd, *J* 18.6, 11.4, 7'-CH_2-H_A), 3.54 (2H, m, 2-CH_2), 3.37 (1H, dd, *J* 11.4, 5.2, 7'-CH_2-H_B), 2.54 (1H, dq, *J* 18.6, 3.1, 5'-CH_2-H_A), 2.29 (2H, s, 3-CH_2), 2.11 (1H, dd, *J* 18.6, 4.7, 5'-CH_2-H_B); δ_C (75 MHz; CD_3OD) 164.4 (carbonyl), 151.8 (isoxazole), 134.0 (C-4), 132.4 (C-4'), 118.6 (C-3'), 118.0 (C-5), 107.9 (isoxazole), 88.4 (C-7), 87.3 (C-8), 61.8 (C-7'), 55.3 (C-6'), 48.4 (C-6), 44.1 (C-2), 42.6 (C-2'), 27.6 (C-5'), 26.8 (C-3); ν_{max}/cm^{-1} (film) 3434, 1638, 1426, 1389; m/z (ES) [M+H] 446.1 (100%, M+H); HRMS Found: 446.0996, $C_{18}H_{19}F_3N_3O_5S_1$ requires 446.0992.

(*R*)-6-(Hydroxymethyl)-*N*-(pyridin-3-yl)-3-((1-(trifluoromethylsulfonyl)-1,2,5,6-tetrahydropyridin-3-yl)ethynyl)-5,6-dihydropyridine-1(2*H*)-carboxamide (69c)

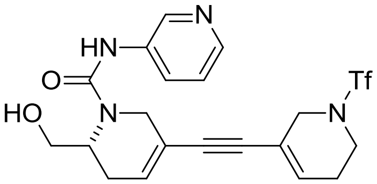

By general method **E2**, the fluorous-tagged amine **56** (0.080 g, 0.087 mmol) gave a crude product, which was purified by F–SPE and subjected to general procedure **D** to give another crude product, which was purified by flash chromatography (gradient elution: 2:98 → 10:90, MeOH–$CHCl_3$) to furnish the urea **69c** (0.032 g, 67%), R_f 0.32 (20:80, MeOH:$CHCl_3$); δ_H (500 MHz; MeOD) 8.49 (1H, s, pyridyl), 8.07 (1H, d, *J* 4.7, pyridyl), 7.81 (1H, dq, *J* 8.1, 1.2, pyridyl), 7.24 (1H, dd, *J* 8.1, 4.7, pyridyl), 6.17 (1H, septate, *J* 2.1, 4-H), 6.12 (1H, dd, *J* 5.9, 2.1, 4'-H), 4.44 (1H, dd, *J* 14.1, 6.8, 6'-H), 4.23 (1H, d, *J* 17.5, 2'-CH_2-H_A), 3.93 (2H, s, 6-CH_2), 3.68 (1H, d, *J* 17.5, 2'-CH_2-H_B), 3.52 (2H, m, 2-CH_2), 3.51 (1H, dd, *J* 11.1, 8.5, 7'-CH_2-H_A), 3.42 (1H, dd, *J* 11.1, 6.4, 7'-CH_2-H_B), 2.43 (1H, dq, *J* 18.3, 2.9, 5'-H_A), 2.28 (2H, s, 3-CH_2), 2.17 (1H, dd, *J* 18.3, 5.9, 5'-H_B); δ_C (75 MHz; MeOD) 158.0 (carbonyl), 144.2 (pyridyl), 142.3 (pyridyl), 133.7 (C-4), 132.6 (C-4'), 130.2 (pyridyl), 125.3 (pyridyl), 118.7 (C-5, 3'), 88.9 (C-8), 87.1 (C-7), 62.2 (C-7'), 51.3 (C-6'), 44.1 (C-2', 2), 27.0 (C-3), 26.8 (C-5'); ν_{max}/cm^{-1} (film) 3315, 2933, 1643, 1600, 1538; m/z (ES) [M+H] 471.1 (100%, M+H); HRMS Found: 471.1312, $C_{20}H_{22}F_3N_4O_4S_1$ requires 471.1308.

(*R*)-6-((6-(Hydroxymethyl)-1,2,5,6-tetrahydropyridin-3-yl)ethynyl)-3,4-dihydro-1*H*-azepin-2(7*H*)-one (70a)

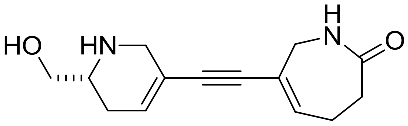

By general method **D**, the fluorous-tagged silyl ether **57** (0.021 g, 0.026 mmol) gave a crude product which was purified by flash chromatography to furnish the alcohol **70a** (0.003 g, 47%), R_f 0.83 (20:80, MeOH:$CHCl_3$); δ_H (500 MHz; CD_3OD) 6.05 (1H, t, *J* 4.2, 5-H), 5.99 (1H, d, *J* 3.8, 4'-H), 4.29 (1H, d, *J* 18.8, 2'-H_A), 3.79 (2H, s, 7-CH_2), 3.76 (1H, d, *J* 6.4, 7'-CH_2-H_A), 3.48 (1H, t, *J* 10.2, 7'-CH_2-H_B), 3.39 (1H, dd, *J* 11.1, 5.7, 5'-CH_2-H_B), 3.13 (1H, m, 6'-H), 2.65 (2H, t, *J* 6.8, 3-CH2), 2.38 (3H, m, 4-CH_2, 5'-CH_2-H_A), 2.08 (1H, dd, *J* 19.2, 8.5, 5'-CH_2-H_B); δ_C (125 MHz; CD_3OD) 179.2 (carbonyl), 164.4, 138.4, 131.3, 121.6, 227.8, 103.9, 95.1, 87.2, 61.6, 59.5, 54.8, 44.5, 40.5, 32.9, 27.3, 26.1, 24.8, 20.7.

(*R*)-6-(Hydroxymethyl)-3-((7-oxo-2,5,6,7-tetrahydro-1*H*-azepin-3-yl)ethynyl)-*N*-(pyridin-3-yl)-5,6-dihydropyridine-1(2*H*)-carboxamide (70c)

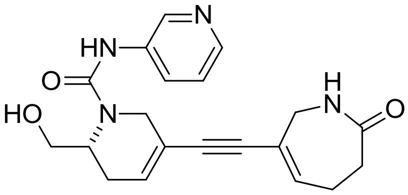

By general method **E2**, the fluorous-tagged amine **57** (0.028 g, 0.034 mmol) gave a crude product, which was purified by F–SPE and subjected to general method **D** to give another crude product which was purified by flash chromatography (gradient elution: 2:98 → 10:90, MeOH–$CHCl_3$) to furnish the urea **70c** (0.004 g, 59%), R_f 0.56 (20:80, MeOH:$CHCl_3$); δ_H (500 MHz; CD_3OD) 8.49 (1H, s, pyridyl), 8.07 (1H, d, *J* 2.1, pyridyl), 7.81 (1H, d, *J* 8.5, pyridyl), 7.25 (1H, dd, *J* 8.5, 5.1, pyridyl), 6.04 (2H, t, *J* 3.8, 5, 4'-H), 4.43 (1H, dd, *J* 14.9, 6.8, 6'-H), 4.20 (1H, d, *J* 17.4, 2'-CH_2-H_A), 3.80 (2H, s, 7-CH_2), 3.65 (1H, d, *J* 17.4, 2'-CH_2-H_B), 3.50 (1H, dd, *J* 11.1, 8.5, 7'-CH_2-H_A), 3.42 (1H, dd, *J* 11.1, 6.4, 7'-CH_2-H_B), 2.60 (2H, t, *J* 6.8, 3-CH_2), 2.40 (3H, m, 4-CH_2, 5'-H_A), 2.15 (1H, dd, *J* 18.8, 5.1, 5'-H_B); δ_C (125 MHz; CD_3OD) 177.9 (carbonyl amide), 156.5 (carbonyl urea), 142.6, 141.3, 136.7, 129.8, 128.5, 120.4, 117.4, 89.0, 86.1, 60.6, 50.2, 43.1, 42.9, 31.9, 25.2, 23.8; m/z (ES) [M+H] 367.2 (100%, M+H); HRMS Found: 367.1777, $C_{20}H_{23}N_4O_3$ requires 367.1765.

((2*R*,6*R*)-6-(((*S*)-1-(Trifluoromethylsulfonyl)-1,2,5,6-tetrahydropyridin-2-yl)methyl)-1,2,3,6-tetrahydropyridin-2-yl)methanol (71a)

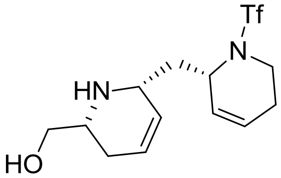

By general method **D**, the fluorous-tagged silyl ether **58** (0.070 g, 0.078 mmol) gave a crude product which was purified by flash chromatography to give the alcohol **71a** (0.024 g, 91%), R_f 0.17 (10:90, MeOH:$CHCl_3$); δ_H (500 MHz; CD_3OD) 5.85 (1H, dd, *J* 10.2, 5.1, 5-H), 5.79 (1H, dq, *J* 10.2, 2.1, 3'-H), 5.72 (1H, td, *J* 10.2, 2.1, 4-H), 5.69 (1H, d, *J* 11, 4'-H), 4.39 (1H, s, 6-H), 3.85 (1H, dd, *J* 14.9, 6.8, 2-CH_2-H_A), 3.66 (1H, m, 2'-H), 3.60 (1H, dd, *J* 11.3, 3.8, 7'-CH_2-H_A), 3.45 (1H, m, 2-CH_2-H_B), 3.42 (1H, dd, *J* 11.5, 7.2, 7'-CH_2-H_B), 3.05 (1H, m, 6'-H), 2.27 (1H, m, 3-CH_2-H_A), 1.96 (4H, m, 3-CH_2-H_B, 5-CH_2, 7-CH_2-H_A), 1.72 (1H, ddd, *J* 14.1, 8.9, 4.7, 7-CH_2-H_B); δ_C (75 MHz; MeOD) 128.2 (C-3'), 128.0 (C-4), 127.0 (C-5), 126.9 (C-3'), 64.9 (C-7'), 57.0 (C-6'), 53.8 (C-6), 53.1 (C-2'), 40.8 (C-2), 40.0 (C-7), 27.7 (C-5'), 25.0 (C-3); m/z (ES) [M+H] 341.1 (100%, M+H); HRMS Found: 341.1150, $C_{13}H_{20}F_3N_2O_3S_1$ requires 341.1141.

((2*R*,6*R*)-6-(Hydroxymethyl)-2-(((*S*)-1-(trifluoromethylsulfonyl)-1,2,5,6-tetrahydro pyridin-2-yl)methyl)-5,6-dihydropyridin-1(2*H*)-yl)(isoxazol-5-yl)methanone (71b)

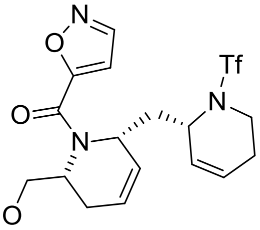

By general method **E4**, the fluorous-tagged amine **58** (0.075 g, 0.083 mmol) was purified by F–SPE and a portion was subjected to general method **D** to give a crude product, which was purified by flash chromatography (gradient elution: 20:80→ 50:50 → 80:20, EtOAc–petrol) to furnish the amide **71b**

(0.006 g, 64%), R_f 0.30 (30:70, EtOAc:petrol); δ_H (500 MHz; CD_3OD) 8.39 (1H, d, *J* 1.5, isoxazole), 6.71 (1H, d, *J* 1.5, isoxazole), 5.87 (1H, d, *J* 10.4, 4'-H), 5.80 (1H, dd, *J* 10.4, 4.5, 3'-H), 5.74 (2H, m, 4, 5-H), 4.76 (1H, dt, *J* 10.4, 2.9, 2'-H), 4.32 (1H, m, 6-H), 4.09 (1H, dd, *J* 14.3, 7.2, 6'-H), 3.88 (1H, dd, *J* 14.1, 6.4, 2-CH_2-H_A), 3.61 (1H, dt, *J* 15.6, 4.0, 2-CH_2-H_B), 3.56 (1H, dd, *J* 10.6, 8.3, 7'-CH_2-H_A), 3.49 (1H, dd, *J* 10.6, 7.2, 7'-CH_2-H_B), 2.31 (2H, m, 5'-CH_2-H_A, 3-CH_2-H_A), 2.11 (2H, m, 5'-CH_2-H_B, 7-CH_2-H_A), 1.99 (1H, dt, *J* 18.5, 5.5, 3-CH_2-H_B), 1.74 (1H, ddd, *J* 14.1, 10.2, 3.8, 7-CH_2-H_B); δ_C (75 MHz; CD_3OD) 161.6 (carbonyl), 151.7 (isoxazole), 128.5, 126.7, 126.1, 123.2, 107.2 (isoxazole), 63.4 (benzyl-CH2), 55.5, 54.2, 49.3, 40.7, 40.3, 26.5, 25.0; m/z (ES) [M+H] 436.1 (100%, M+H); HRMS Found: 436.1135, $C_{17}H_{21}F_3N_3O_5S_1$ requires 436.1149.

(2*R*,6*R*)-6-(Hydroxymethyl)-*N*-(pyridin-3-yl)-2-(((S)-1-(trifluoromethylsulfonyl)-1,2,5,6-tetrahydropyridin-2-yl)methyl)-5,6-dihydropyridine-1(2*H*)-carboxamide (71c)

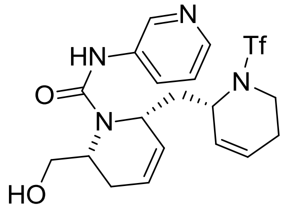

By general method **E2**, the fluorous-tagged amine **58** (0.070 g, 0.078 mmol) gave a crude product, which was purified by F–SPE and subjected to general method **D** to give another crude product, which was purified by flash chromatography (gradient elution: 2:98 → 10:90, MeOH–$CHCl_3$) to furnish the urea **71c** (0.015 g, 53%), R_f 0.35 (10:90, MeOH:$CHCl_3$); δ_H (500 MHz; CD_3OD) 8.41 (1H, d, *J* 2.3, pyridyl), 8.06 (1H, d, *J* 4.2, pyridyl), 7.75 (1H, dq, *J* 8.3, 1.5, pyridyl), 7.24 (1H, dd, J 8.3, 4.2, pyridyl), 5.87 (1H, d, *J* 10.4, 4'-H), 5.75 (3H, m, 4, 5, 3'-H), 4.62 (1H, dd, *J* 6.4, 2.6, 2'-H), 4.30 (1H, m, 6-H), 4.20 (1H, q, *J* 7, 6'-H), 3.81 (1H, dd, *J* 14.3, 6.8, 7'-CH_2-H_A), 3.56 (2H, dd, *J* 7.2, 1.5, 2-CH_2), 3.51 (1H, m, 7'-CH_2-H_B), 2.27 (2H, m, 5'-CH_2-H_A, 3-CH_2-H_A), 2.11 (1H, dd, *J* 18.1, 5.1, 5'-CH_2-H_B), 1.98 (2H, m, 3-CH_2-H_B, 7'-CH_2-H_A), 1.78 (1H, ddd, *J* 14.3, 9.8, 4.7, 7'-CH_2-H_B); δ_C (75 MHz; CD_3OD) 158.4 (carbonyl), 144.0 (pyridyl), 142.7 (pyridyl), 139.1 (pyridyl), 130.0, 128.6, 126.5, 125.4, 127.0 (pyridyl), 123.0 (pyridyl), 64.8 (C-7'), 54.3 (C-6'), 52.3 (C-6), 48.9 (C-2'), 41.8 (C-7), 26.5 (C-5'), 25.0 (C-3); ν_{max}/cm^{-1} (film) 3390, 2927, 1650, 1533, 1422; m/z (ES) [M+H] 461.1 (100%, M+H); HRMS Found: 461.1476, $C_{19}H_{24}F_3N_4O_4S_1$ requires 461.1465.

((2*R*,6*R*)-6-(Hydroxymethyl)-2-(((*S*)-1-(trifluoromethylsulfonyl)-1,2,5,6-tetrahydropyridin-2-yl)methyl)-5,6-dihydropyridin-1(2*H*)-yl)(morpholino)methanone (71d)

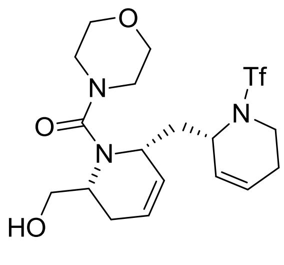

By general method **E3**, the fluorous-tagged amine **58** (0.075 g, 0.083 mmol) gave a crude product, which was purified by F–SPE and subjected to general method **D** to give another crude product, which was purified by flash chromatography (gradient elution: 2:98 → 5:95, MeOH–$CHCl_3$) to furnish the urea **71d** (0.007 g, 29%), R_f 0.26 (5:95, MeOH:$CHCl_3$); δ_H (500 MHz; CD_3OD) 5.76 (3H, m), 5.62 (1H, m), 4.37 (2H, m), 4.28 (1H, m), 4.21 (1H, dd, *J* 14.1, 6.7, 6'-H), 4.06 (1H, dd, *J* 10.9, 3.6, 7'-CH_2-H_A), 3.98 (1H, dd, *J* 10.9, 6.7, 7'-CH_2-H_B), 3.82 (2H, m), 3.50 (4H, m), 2.27 (3H, m), 2.07 (5H, m), 1.69 (1H, m); δ_C (75 MHz; CD_3OD) 150.1, 126.7, 126.5, 124.9, 124.5, 123.9, 66.1, 65.2, 62.0, 61.9, 52.9, 52.4, 51.8, 50.5, 48.945.6, 44.3, 39.2, 24.3, 23.9; m/z (ES) [M+H] 454.2 (100%, M+H); HRMS Found: 454.1619, $C_{18}H_{27}F_3N_3O_5S_1$ requires 454.1618.

7 Chiral HPLC analysis of the amide S5

Racemate

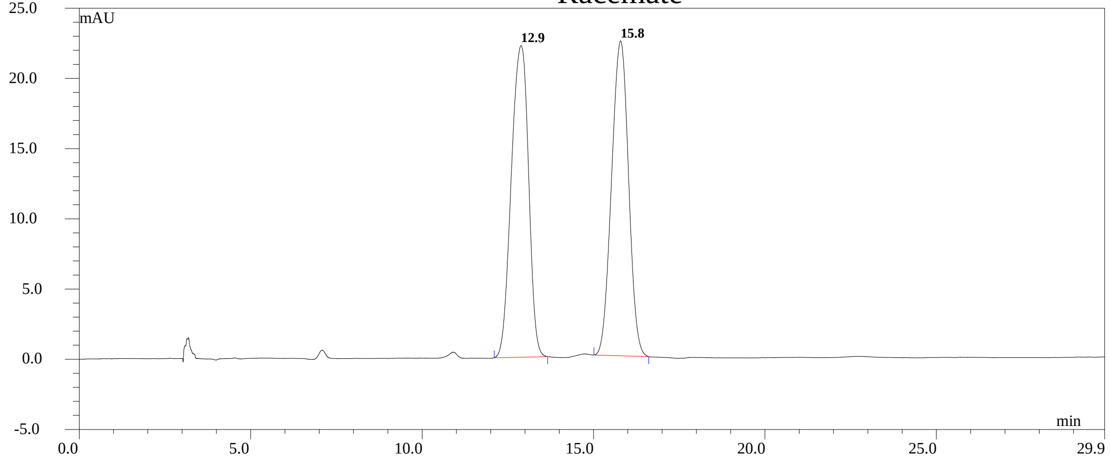

(*R*)-S5 (prepared from (*R*)-2-amino-4-pentenoic acid)

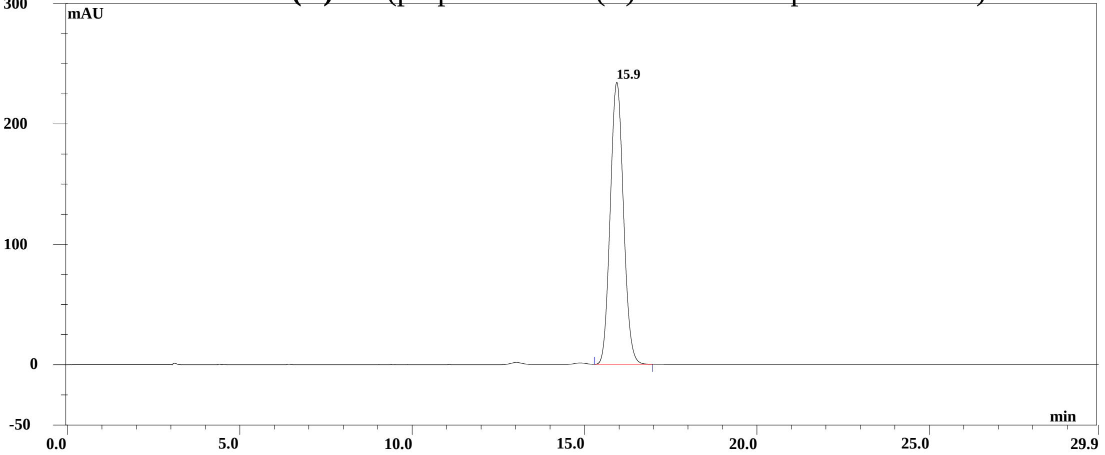

(*R*)-S5 (prepared from **22**)

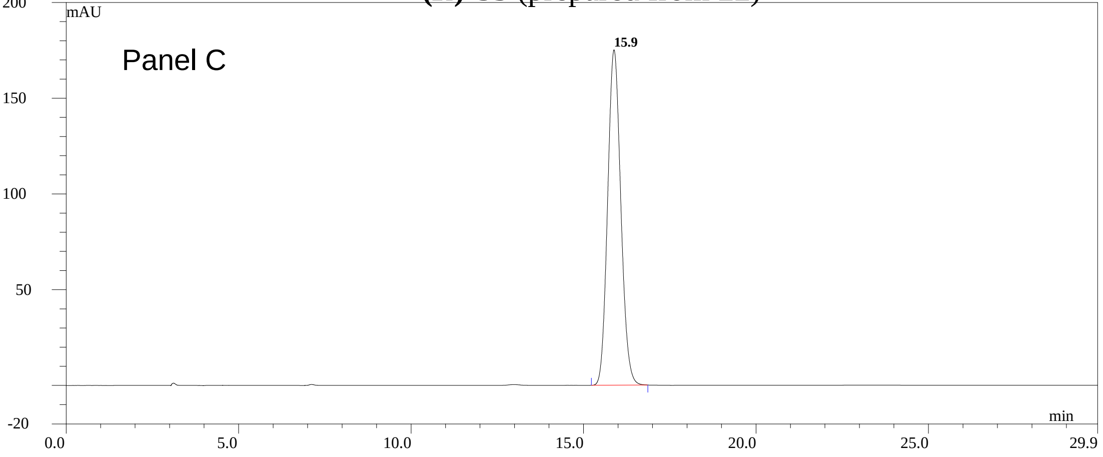

8 References

S1. Morton, D.; Leach, S.; Cordier, C.; Warriner, S.; Nelson, A. *Angew. Chem. Int. Ed.* **2008**, *47*, 104–109.

S2. Mak,C. C; Bampos, N.; Darling, S. L; Montalti, M; Prodi, L; Sanders, J. K. M. *J. Org. Chem.* **2001,** *66*, 4476–4486.

S3. Motoshima, K.; Ishikawa, M.; Hashimoto, Y.; Sugita, K. *Bioorg. Med. Chem.* 2011, 19, 3156–3172.

S4. Rech, J. C.; Yato, M.; Duckett, D.; Ember, B.; LoGrosso, P. V.; Bergman, R. G.; Ellman, J. A. *J. Am. Chem. Soc.* **2007**, *129*, 490–491.
